# Synthesis, solution dynamics and chemical vapour deposition of heteroleptic zinc complexes *via* ethyl and amide zinc thioureides†

**DOI:** 10.1039/d1sc01846a

**Published:** 2021-05-24

**Authors:** Malavika A. Bhide, Kristian L. Mears, Claire J. Carmalt, Caroline E. Knapp

**Affiliations:** Materials Chemistry Centre, Department of Chemistry, University College London 20 Gordon Street London WC1H 0AJ UK caroline.knapp@ucl.ac.uk

## Abstract

Ethyl and amide zinc thioureides [L^1^ZnEt]_2_ (**1**), [L^1^*ZnEt]_2_ (**2**) and [L^1^Zn(N(SiMe_3_)_2_)]_2_ (**3**) have been synthesised from the equimolar reaction of thiourea ligands (HL^1^ = ^i^PrN(H)CSNMe_2_ and HL^1^* = PhN(H)CSNMe_2_) with diethyl zinc and zinc bis[bis(trimethylsilyl)amide] respectively. New routes towards heteroleptic complexes have been investigated through reactions of **1**, **2** and **3** with β-ketoiminates (HL^2^ = [(Me)CN(H){^i^Pr}–CHC(Me)=O]), bulky aryl substituted β-diiminates (HL^3^ = [(Me)CN(H){Dipp}–CHC(Me)=N{Dipp}] (Dipp = diisopropylphenyl) and HL^3^* = [(Me)CN(H){Dep}–CHC(Me)=N{Dep}] (Dep = diethylphenyl)) and donor-functionalised alcohols (HL^4^ = Et_2_N(CH_2_)_3_OH and HL^4^* = Me_2_N(CH_2_)_3_OH) and have led to the formation of the heteroleptic complexes [L^1^*ZnL^3^*] (**5**), [L^1^ZnL^4^]_2_ (**6**), [L^1^ZnL^4^*]_2_ (**7**), [L^1^*ZnL^4^] (**8**) and [L^1^*ZnL^4^*] (**9**). All complexes have been characterised by ^1^H and ^13^C NMR, elemental analysis, and the X-ray structures of HL^1^*, **1**, **2**, **6** and **7** have been determined *via* single crystal X-ray diffraction. Variable temperature ^1^H, COSY and NOESY NMR experiments investigating the dynamic behaviour of **5**, **6** and **7** have shown these molecules to be fluxional. On the basis of solution state fluxionality and thermogravimetric analysis (TGA), alkoxyzinc thioureides **6** and **7** were investigated as single-source precursors for the deposition of the ternary material zinc oxysulfide, Zn(O,S), a buffer layer used in thin film photovoltaic devices. The aerosol-assisted chemical vapour deposition (AACVD) reaction of **7** at 400 °C led to the deposition of the heterodichalcogenide material Zn(O,S), which was confirmed by X-ray diffraction (XRD), X-ray photoelectron spectroscopy (XPS) and energy dispersive X-ray analysis (EDX), with optical properties investigated using UV/vis spectroscopy, and surface morphology and film thickness examined using scanning electron microscopy (SEM).

## Abstract 2

This work investigates the synthesis and solution dynamics of heteroleptic alkoxyzinc thioureides for the chemical vapour deposition of the heterochalcogenide material Zn(O,S).

## Graphical abstract

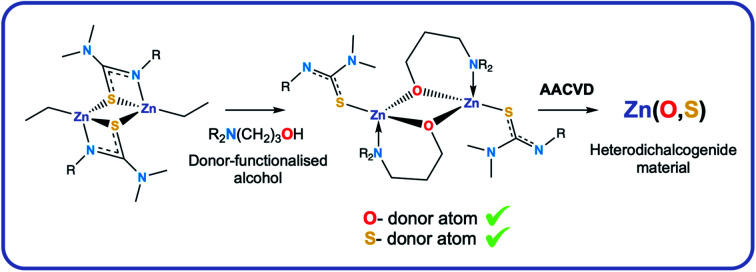

## Introduction

Reports of the deposition of the ternary material zinc oxysulfide, Zn(O,S), are far fewer as compared to semiconductors such as zinc oxide, zinc sulfide and tin oxide.^1–3^ This is in part due to Zn(O,S) being an emerging alternative for use as a buffer layer in photovoltaic (PV) devices.^4,5^ It is more so due to the lack of reports of single source precursors (SSPs) towards Zn(O,S), especially *via* chemical vapour deposition (CVD) methods, as use of SSPs is mostly limited to binary materials. In the CVD literature, high purity materials are formed using dual source vapour deposition processes, including doped transparent conducting oxides (TCOs) such as F:ZnO,^6^ Ga:ZnO^7–9^ and Al:ZnO,^10^ and ternary and quaternary materials such as Ga_*x*_In_2−*x*_O_3_,^11^ CZTS^12^ and MAPbI_3_.^13^ This poses the question – why would SSPs be advantageous for the deposition of ternary materials? SSPs reduce the number of synthetic steps and also avoid the use of toxic gaseous reagents such as H_2_S, which is often used as a sulfur source in both the CVD and atomic layer deposition (ALD) of metal sulfides.^14^ Additionally, in dual source processes, as two or more different precursors are mixed in a one-pot reaction, the precursors have differing decomposition profiles, solubility, volatility, vapour pressure, and thus may not always be compatible. Furthermore, film growth is highly dependent on the local environment, with preferential growth in certain planes due to influence from co-dopants.^15^ SSPs also inhibit premature reaction of co-reagents and produce films with less stoichiometric variation. In fact, combinatorial CVD studies using a dual source approach have shown the extent of variation in film stoichiometry.^16,17^ Because SSPs are singular molecules, these factors become obsolete, the deposition process is dependent on fewer variables, and so is more robust and repeatable.

SSPs towards ternary materials need to be heteroleptic molecules, to incorporate bonds between all the desired elements required in the resultant film. Heterobimetallic complexes have been synthesised and characterised in the literature for their use as SSPs towards ternary materials.^18–20^ Thermal decomposition studies show that these precursors decompose to the desired ternary or quaternary materials, however, they tend to be large cluster complexes, not ideally suited to CVD. Monomeric precursors however, are favoured as they can be easily vaporised and upon decomposition, carbon or halogen contamination in the resultant deposit will be lower. Overcoming contamination is crucial since it is well known that carbon or chlorine contamination has detrimental effects on optoelectronic properties of certain TCO materials, such as a change in conductivity or charge carrier concentration. There are few examples of monomeric heteroleptic complexes that have been used as precursors towards ternary materials. Carbonitride materials have been deposited from a range of precursors: a tungsten imido precursor was used for the CVD of tungsten carbonitride, WC_*x*_N_*y*_,^21^ titanium and zirconium carbonitride films were deposited from titanium(iv) and zirconium guanidinate precursors respectively *via* CVD,^22,23^ and silicon carbonitride was deposited *via* plasma-enhanced (PE)CVD from a carbon rich silazane precursor.^24^ Though these precursors do deposit the desired carbonitride materials, imido and guanidinate precursors do not contain direct metal–carbon bonds.

It has been shown in the literature that the CVD of zinc complexes with a chelating ligand containing both an O- and S-donor atom such as thiobiuret ligands or thioacetate ligands serve as precursors towards the metal sulfide only, with no oxygen incorporation unless an external oxygen source is used.^25,26^ Recently, nitrogen-doped molybdenum disulfide (N-MoS_2_) was deposited *via* CVD from the monomeric heteroleptic imido-thiolato precursor Mo(N^*t*^Bu)_2_(S^*t*^Bu)_2_, which contains two different ligands bound to the molybdenum centre, one being a nitrogen source and the other being a sulfur source.^27^ The film had optoelectronic properties comparable to those of N-MoS_2_ films grown *via* dual source methods.^28^

**Scheme 1:**
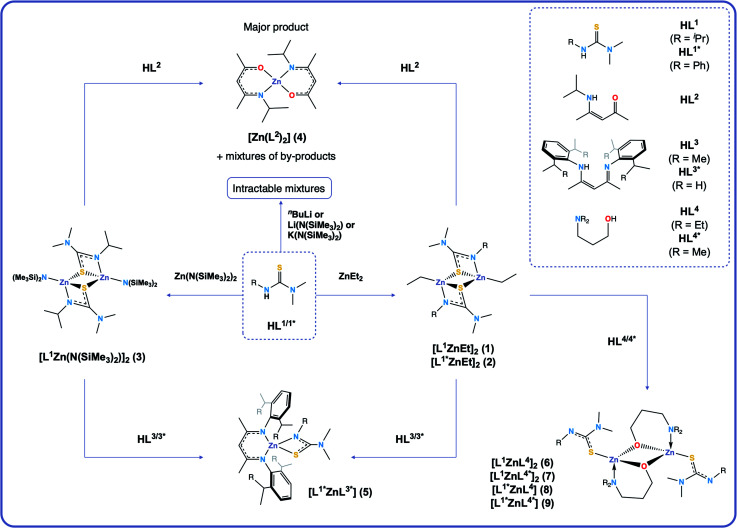
Synthetic routes towards complexes **1–9**.

In this work we show that the CVD of heteroleptic zinc complexes with one ligand containing an O-donor atom and another containing an S-donor atom leads to deposition of Zn(O,S). We have investigated the structural and dynamic properties of heteroleptic zinc complexes formed *via* ethyl and amide zinc thioureides as intermediates, by systematically changing the oxygen donor ligands (Scheme 1), with a view to utilising them as SSPs to the ternary material zinc oxysulfide, Zn(O,S). Bulky aryl substituted β-diiminate (BDI) ligands have been used to investigate whether it was possible to form heteroleptic complexes with ligands that form a stable 6-membered rings upon coordination to the zinc centre. Formation of heteroleptic species is possible with careful ligand choice.^29^ Donor-functionalised alcohols have been used to synthesise heteroleptic dimeric alkoxyzinc thioureide complexes, and using VT-NMR, we have shown that these molecules are fluxional in solution. We have elucidated a link between this fluxionality and the thermal properties (*via* TGA) of the precursors, and have demonstrated their use as SSPs towards Zn(O,S) *via* AACVD.

## Results and discussion

### Synthesis

#### Synthesis of ethyl zinc and amide zinc thioureides

Taking inspiration from Sullivan *et al.*,^30^ thiourea ligands were chosen as the sulfur source due to their facile, high yielding synthesis which circumvents issues with usage of H_2_S gas and other malodourous sulfur containing compounds. Their work also showed that zinc thioureides were effective SSPs towards phase pure zinc sulfide. The odourless, colourless, air stable powders [^i^PrN(H)CSNMe_2_] (HL^1^) and [PhN(H)CSNMe_2_] (HL^1^*) were synthesised by reaction of isopropyl isothiocyanate and phenyl isothiocyanate respectively with a solution of dimethylamine in hexane. After precipitation and filtration, HL^1^ and HL^1^* were obtained as white powders in high yield and were both recrystallised from toluene solutions. Their formation was confirmed by ^1^H and ^13^C NMR. Recrystallisation of HL^1^* yielded colourless crystals suitable for analysis by single crystal XRD which further confirmed its structure (ESI†).

**Fig. 1:**
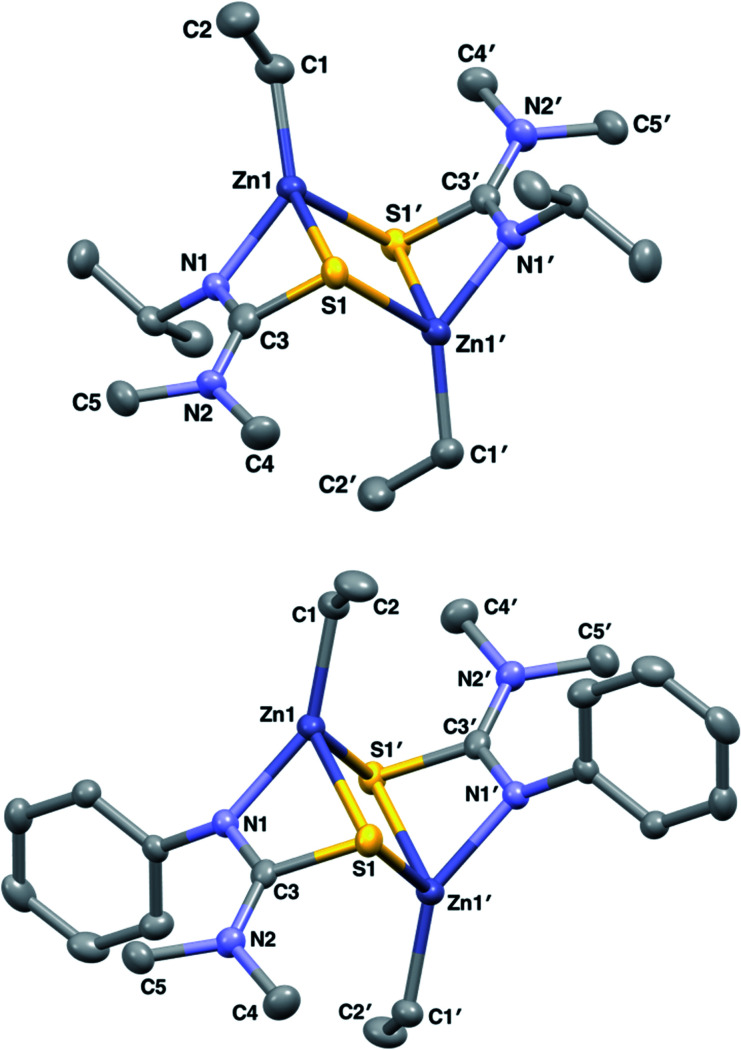
Solid state structures of **1** and **2** with thermal ellipsoids drawn at 50% probability and hydrogen atoms omitted for clarity.

Initially, both salt metathesis and acid–base routes towards heteroleptic compounds were considered. Reaction of lithium or potassium salts of HL^1/1^* with ZnCl_2_ would yield the chlorozinc thioureide, and it was hypothesised that upon further reaction with a lithium or potassium salt of a second ligand, the desired heteroleptic complex could be obtained. However, attempts were made to synthesise lithium and potassium salts of HL^1^ and HL^1^* but reactions of the ligands with ^*n*^BuLi, Li(N(SiMe_3_)_2_) and K(N(SiMe_3_)_2_) led to intractable mixtures making the salt metathesis route synthetically unfeasible. Acid–base reactions using diethyl zinc and free ligand were then employed. The ethyl zinc thioureide complexes [L^1^ZnEt]_2_ (**1**) and [L^1^*ZnEt]_2_ (**2**) were synthesised by the equimolar reaction of diethylzinc with HL^1^ and HL^1^* respectively in toluene, with the liberation of ethane gas. Removal of the solvent *in vacuo* afforded the complexes as white powders in high yield. The formation of **1** and **2** was confirmed by ^1^H and ^13^C NMR spectroscopy, as well as single crystal XRD, which confirmed the structures as dimeric species in the solid state (Fig. 1). Peaks observed in the ^1^H NMR spectrum of **1** at 1.04, 2.59 and 3.51 ppm corresponding to the CH(C*H*_3_)_2_, N(C*H*_3_)_2_ and C*H* protons of the thioureide ligand respectively appeared in a ratio of 6 : 6 : 1, with two peaks at 0.82 and 1.77 ppm assigned to the bound ethyl group protons appearing in a ratio of 2 : 3 respectively. The characteristic triplet–quartet peaks assigned to the bound ethyl group appeared as sharp signals. Peaks observed in the ^1^H NMR spectrum of **2** at 2.35 and between 6.76 and 7.02 ppm corresponding to the N(C*H*_3_)_2_ and aryl protons of the thioureide ligand respectively appeared in a ratio of 6 : 5, with similar triplet–quartet peaks as in **1** assigned to the bound ethyl group. **1** crystallised out of a toluene/hexane solution as a dimer in the monoclinic space group *I*2/*a* whilst **2** crystallised out of a C_6_D_6_ solution as a dimer in the monoclinic space group *P*2_1_/*n*, with the four coordinate zinc centres in both complexes adopting highly distorted tetrahedral geometries (
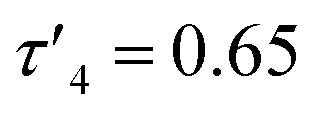
 (**1**) and 0.61 (**2**)). Both structures are analogous to each other and also to reported methyl zinc thioureides, with dimerization occurring through S atoms and formation of a Zn–S–Zn–S ring structure.^30^ The C–S bond in **2** was significantly lengthened as compared to the free ligand HL^1^* (1.7678(16) Å and 1.6852(15) Å respectively) whilst the C–N(Ph) bond was shortened (1.323(2) Å and 1.3715(18) Å) upon coordination to the zinc centre. The C–N(Me_2_) bond in HL^1^* was also significantly shorter than in **2**. Upon coordination to the zinc centre, the S–C–N(Ph) bond angle in **2** was narrowed as expected, with the N–C–N bond angle widened and a slight narrowing of the S–C–N(Me_2_) bond angle as compared to the free ligand HL^1^* (Table 1).

**Table 1:** Selected bond lengths (Å) and angles (°) for HL^1^* and **2**

	HL^1^*	**2**
**Bond lengths/Å**		
C–S	1.6852(15)	1.7678(16)
C–N(Ph)	1.3715(18)	1.323(2)
C–N(Me_2_)	1.3374(19)	1.341(2)

**Bond angles/°**		
S–C–N(Ph)	121.92(11)	113.45(12)
S–C–N(Me_2_)	122.79(11)	119.87(12)
N–C–N	115.29(13)	126.56(15)

As well as ethyl zinc intermediates, amide zinc thioureides were also considered. [L^1^Zn(N(SiMe_3_)_2_)]_2_ (**3**) was synthesised by the equimolar reaction of [Zn(N(SiMe_3_)_2_)_2_] with HL^1^ in toluene, with removal of the solvent *in vacuo* affording the complex as a white powder. Formation of **3** was confirmed by ^1^H NMR, which showed signals at 1.15, 2.64 and 3.57 ppm in a 6 : 6 : 1 ratio corresponding to the CH(C*H*_3_)_2_, N(C*H*_3_)_2_ and C*H* protons of the thioureide ligand respectively and the signal at 0.46 ppm corresponding to the bound amide group protons integrating to 18, as expected. In addition, analytical scale reactions showed the formation of bis(trimethylsilyl)amine in the correct stoichiometric ratio (18H), further confirming the formation of **3**. Crystals suitable for single crystal XRD were obtained after recrystallisation from a concentrated toluene solution and confirmed the structure as a dimeric species in the solid state, in agreement with previous literature.^30^

#### Reactions of ethyl zinc and amide zinc thioureides with HL^2^ and HL^3^

Initially, β-ketoiminates (BKI) ligands were chosen as the oxygen source as they have facile, high yielding syntheses and BKI metal complexes have been shown to be excellent CVD precursors towards metal oxides.^10,31–34^ Following standard synthetic routes for the preparation of BKI ligands, [(Me)CN(H){^i^Pr}–CHC(Me)=O] (HL^2^) was synthesised *via* a simple 1 : 1 condensation reaction of acetylacetone and isopropylamine and isolated as an orange oil in high purity and good yield.

Targeting heteroleptics of the form [L^1^ZnL^2^], equimolar reactions of **1**, **2** and **3** with HL^2^ were carried out. However, these led to formation of the bis-ligated zinc BKI complex [Zn(L^2^)_2_] (**4**), along with a mixture of by-products, as confirmed by ^1^H NMR (ESI†). Firstly, we theorised that the thermodynamic stabilisation when forming two six membered conjugated rings in **4** was much higher than for the formation of a heteroleptic with one BKI ligand and one thioureide ligand. Secondly, we considered the possibility of a ligand scrambling reaction occurring, as two BKI ligands are required to coordinate to the zinc centre. We proposed that L^2^ coordinates to a zinc centre after liberation of EtH, causing weakening of the second already bound thioureide ligand, causing it to dissociate. This can then become protonated by a second HL^2^ molecule, which in turn is free to coordinate to the zinc centre, affording the bis-ligated BKI complex, **4**.

To mitigate these factors, attention was turned to β-diiminate (BDI) ligands with large sterically demanding groups: [(Me)CN(H){Dipp}–CHC(Me)=N{Dipp}] (Dipp = diisopropylphenyl) (HL^3^) and [(Me)CN(H){Dep}–CHC(Me)=N{Dep}] (Dep = diethylphenyl) (HL^3^*). These ligands are electronically similar to HL^2^, which would result in two six membered conjugated rings at the Zn centre if the bis complex formed. However, the increased steric bulk could potentially facilitate formation of a heteroleptic complex before ligand scrambling reactions occurred. Therefore, HL^3^ and HL^3^* were synthesised from the acid-catalysed 1 : 2 condensation reaction of acetylacetone with diisopropylaniline and diethylaniline respectively and isolated as pale yellow crystalline solids in high purity and good yield. These ligands are ubiquitous in coordination chemistry and have been used to stabilise a wide range of low valent metal centres.^35–37^ Compound **1** was combined with HL^3^ in an equimolar ratio and heated to 100 °C for two weeks. Reaction monitoring through ^1^H NMR showed that the desired product was forming (ESI†), but that free HL^3^ still remained in the reaction mixture. After addition of excess **1** the reaction did not go to completion (ESI†). A similar situation occurred when reacting **2** and **3** with HL^3^. Only reactions with the ethyl compounds were carried forward as this route was synthetically cleaner than the amide route. Reaction of excess **2** with less bulky HL^3^* required heating at 100 °C for six days after which the reaction went to completion and no free ligand peaks were seen in the ^1^H NMR spectrum. This was attributed to the lessened steric bulk of BDI–Dep as compared to BDI–Dipp.

**Fig. 2:**
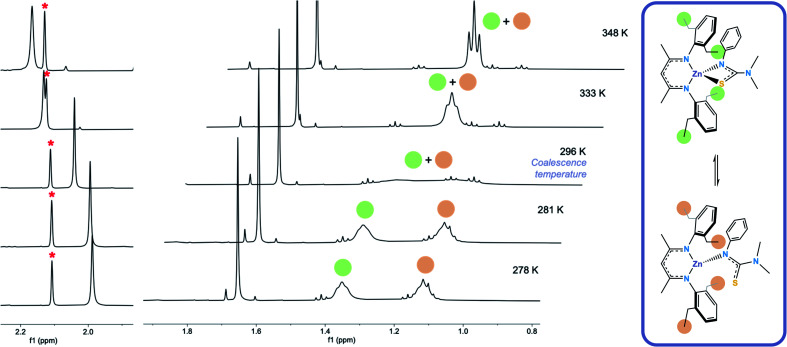
Variable temperature ^1^H NMR spectrum of **5** in C_6_D_6_, showing the upfield shift of the –N*Me*_2_ proton signal (left) and the splitting of the terminal proton signals on the ethyl arms of the BDI–Dep ligand (centre) as temperature is decreased. The hypothesised dynamic process occurring in solution in **5** (right).

Formation of [L^1^*ZnL^3^*] (**5**) was confirmed *via*^1^H and ^13^C NMR. The room-temperature ^1^H NMR spectrum of **5** showed sharp peaks at 1.66 ppm and 4.88 ppm in a ratio of 6 : 1 corresponding to the carbon backbone methyl group protons and the methine proton on the BDI–Dep ligand respectively, and at 2.04 ppm (6H) corresponding to the –N*Me*_2_ protons on the thioureide ligand. Signals in the aryl region were also sharp and integrated to the expected 11 protons from the aryl protons on both ligands. The spectrum also showed a number of indistinguishable broad peaks corresponding to the remaining protons, on the ethyl arms, which integrated to ∼20 protons, as expected. This led us to believe that two distinctly different, rapidly interconverting structures existed in the solution state and variable temperature ^1^H NMR was used to investigate this dynamic behaviour (Fig. 2). Low temperature NOESY NMR experiments revealed two signals at 1.11 and 1.35 ppm corresponding to the CH_3_ protons on the ethyl arms and four distinct resonances at 2.41, 2.54, 2.85 and 3.25 ppm corresponding to the CH_2_ protons on the ethyl arms of the BDI–Dep ligand (ESI†).

We theorised that this fluxionality was due to the S atom of the thioureide ligand in **5** associating and dissociating from the Zn centre in solution. At high temperature, the association and dissociation of the S atom occurs at a faster rate than that of the NMR timescale, leading to the observed time averaged signal, the triplet at 1.21 ppm, integrating to 12 protons. The room temperature spectrum shows a broad coalesced peak between 1–1.4 ppm and at low temperature, this rapid interconversion slows down and the peak splits into two distinct resonances which are observed at 1.12 and 1.35 ppm, integrating to 6 protons each (Fig. 2). This fluxional behaviour was corroborated by the downfield shift of the –N*Me*_2_ resonance upon increasing temperature. It is hypothesised that upon dissociation of the S atom, the C–S bond lengthens, and the structure of the ligand becomes more similar to its protonated form. The –N*Me*_2_ protons become more deshielded and the resonance shifts downfield (the signal for these protons in the free ligand peak appears at 2.56 ppm, ESI†). The signal for these protons at higher temperature is also broader; because these atoms are four bonds away from the zinc centre, we would not expect them to be affected to such an extent as the ethyl arms on the BDI–Dep ligand by change in environment around the zinc centre. This is also corroborated by the BDI backbone carbon protons remaining as a single resonance, and not splitting or shifting significantly with change in temperature. This is consistent with the splitting of the ethyl arm proton resonances being due to a local change in environment as hypothesised.

These findings also supported the theory of ligand scrambling reactions in the reactions of **1**, **2** and **3** with HL^2^. As we have shown that the thioureide ligands are fluxional in **5**, it can be inferred that once a BKI ligand had coordinated to a zinc centre in **1**, **2** or **3**, the thioureide ligand would dissociate. The BKI ligand would not be sterically hindered enough to trap the thioureide ligand and keep the heteroleptic molecule intact once it had formed. In the case of the BDI ligand, there was enough steric bulk to keep the thioureide ligand bound, leading to the observed association and dissociation of the S atom, without complete dissociation of the thioureide ligand. Electronic factors cannot be ruled out, and due to its two donor oxygen atoms, the electronic stabilisation of **4** may also be greater than that of the bis-ligated BDI complex [Zn(L^3^)_2_].

Investigations with bulky BDI ligands showed that heteroleptic complexes can indeed be formed but judicious choices about the steric and electronic effects of ligands needed to be considered if these molecules are to be useful as precursors for CVD application.

#### Reactions of ethyl zinc thioureides with HL^4^ and HL^4^*

Another class of oxygen donor ligand, donor-functionalised alcohols, were then chosen as the oxygen source. Complexes bearing donor-functionalised alcohols have been reported in the literature,^38^ some of which have been used as CVD precursors towards oxide materials.^39,40^ These ligands have the same donor atoms as BKI ligands but are electronically different: there is no conjugation, and the N atom coordinates datively to the metal centre, whilst the O atom formally bonds to it. Previous work from our group has shown that these ligands exhibit hemilabile coordination to a metal centre,^40^ and because the bis-BKI complex **4** formed so readily, we theorised that the lability of donor-functionalised alcohols would allow for formation of heteroleptic complexes. Three-carbon backbone aminoalcohols were chosen to mimic the sterics of BKI and BDI ligands. Equimolar reactions of **1** and **2** with the donor-functionalised alcohols Et_2_N(CH_2_)_3_OH (HL^4^) and Me_2_N(CH_2_)_3_OH (HL^4^*) afforded a set of heteroleptic complexes: [L^1^ZnL^4^]_2_ (**6**), [L^1^ZnL^4^*]_2_ (**7**), [L^1^*ZnL^4^] (**8**) and [L^1^*ZnL^4^*] (**9**) as shown by ^1^H and ^13^C NMR, as well as single crystal XRD, which confirmed the structures of **6** and **7** as dimeric species in the solid state.

The structures of **6** and **7** are analogous as expected, with the four coordinate zinc atoms adopting distorted tetrahedral geometries in both structures 
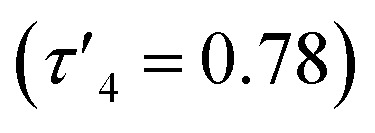
. Both structures crystallised in the monoclinic lattice system; **6** in the *P*2_1_/*n* and **7** in the *P*2_1_/*c* space groups. Bond lengths for compounds **6** and **7** are comparable, with no unusual or significant difference between them (Table 2). The bite angle of the donor functionalised alcohol ligand in **7** was smaller than in **6** (85.82(7)° *vs.* 96.95(7)°), however, the Zn–O1 and Zn–N1 bond lengths in both complexes were comparable. This indicates that the ligand in **7** may experience more ring strain than in **6** as the bite angle deviates further from the ideal 109.5° tetrahedral angle. The torsion angle N1–Zn–S–NMe_2_ in **6** was greater than in **7** due to the steric constraints of the ethyl groups on the donor functionalised alcohol preventing the –NMe_2_ group of the thioureide ligand to be proximate to the zinc centre.

**Table 2:** Selected bond lengths (Å) and angles (°) for **6** and **7**

	**6**	**7**
**Bond lengths/Å**		
Zn–O1	1.9761(16)	1.9760(13)
Zn–S1	2.2532(7)	2.2508(5)
Zn–N1	2.0882(19)	2.0791(17)
O–C	1.411(3)	1.420(2)
S–C	1.791(3)	1.7843(18)

**Bond angles/°**		
O1–Zn–N1	96.95(7)	85.82(7)
O1–Zn–O1′	98.85(7)	85.33(5)

**Torsion angles/°**		
N1–Zn–S–NMe_2_	46.67(19)	36.47(13)

**Fig. 3:**
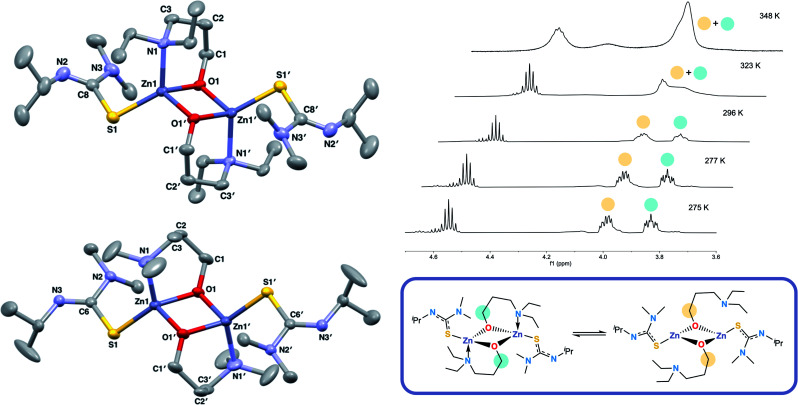
Solid state structures of **6** (top left) and **7** (bottom left) with thermal ellipsoids drawn at 50% probability and hydrogen atoms omitted for clarity. Variable temperature ^1^H NMR showing the splitting of the –OC*H*_2_ proton signals in **6** upon decrease in temperature and coalescing into a single peak at 348 K (top right) and the hypothesised dynamic process occurring in solution in **6** (bottom right).

**Fig. 4:**
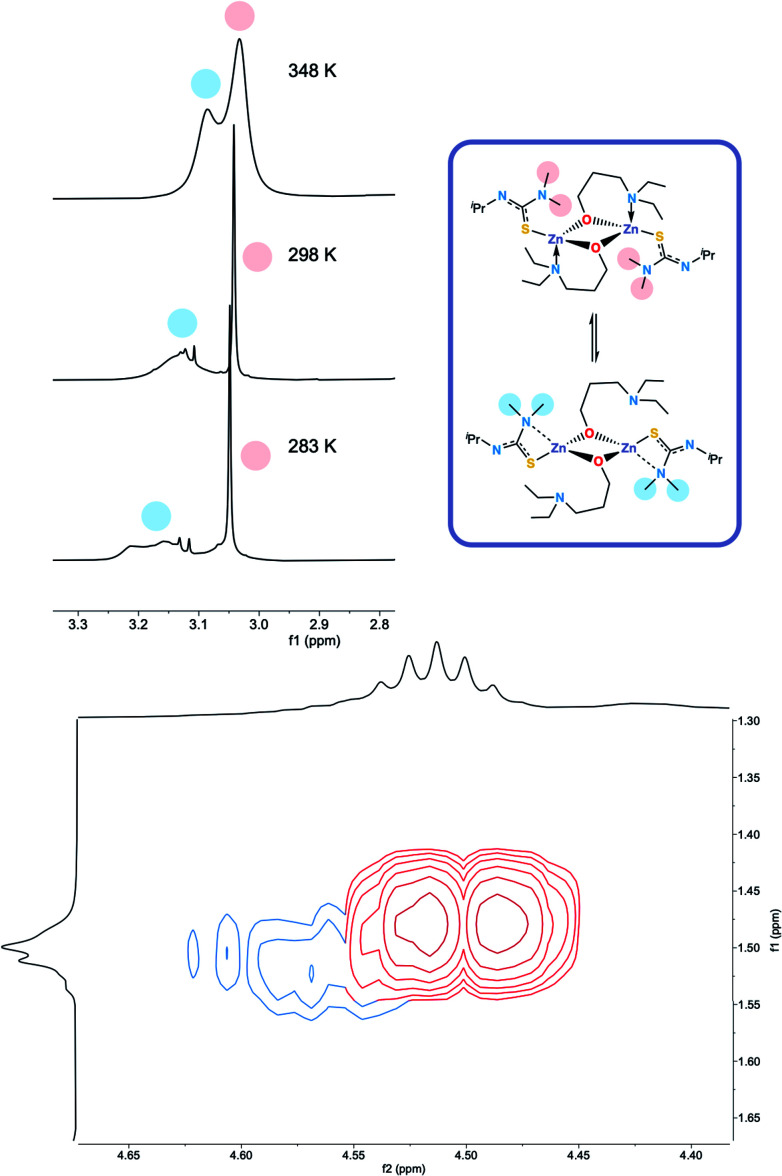
(Top left) ^1^H NMR showing the two distinct resonances for the –N*Me*_2_ protons in **7**, (top right) hypothesised dynamic process occurring in solution in **7**. (Bottom) COSY NMR spectrum of **7** showing the pair of two distinct cross peaks between the protons in the ^i^Pr group of the thioureide ligand. The red trace indicates the top structure, the blue trace indicates the bottom structure.

The ^1^H NMR spectra of **6** exhibits broad signals for the donor-functionalised alcohol protons at room temperature, as well as distinctly separate resonances for protons that are bound to the same carbon atom (as confirmed *via*^1^H–^1^H 2D NMR (ESI†)). This indicates some degree of fluxionality in solution. It is hypothesised that the structure exists in two forms: one in which the nitrogen atoms of the donor functionalised alcohol are coordinated to the zinc centres (as is seen from the solid state structure) and the second where they dissociate from the zinc atom and are no longer coordinated (Fig. 3, boxed). At room temperature and below, this interconversion is slow enough to lead to two distinct resonances being recorded, whilst at high temperature, the interconversion is faster than that of the NMR timescale, leading to the observed single resonance (Fig. 3). In contrast to **5**, the ^1^H NMR signals for the –N*Me*_2_ or any of the protons on the thioureide ligand do not shift significantly upon varying temperature of **6**. This further corroborates that the fluxionality is taking place in the donor-functionalised alcohol ligands, and that the fluxionality in **5** is taking place in the thioureide ligand. The ^1^H NMR of **7** also consists of broad indistinguishable peaks, which upon cooling do not resolve as readily, indicating a greater amount of fluxionality. The COSY NMR spectrum of **7** revealed that in addition to fluxionality of the donor functionalised alcohol, there was also fluxionality in the thioureide ligand. Pairs of peaks for all protons on the thioureide ligand were observed, with the –N*Me*_2_ proton signal pair being the most prominent, evidence of two distinctly different structures being present in solution (Fig. 4). Due to the smaller substituents on the N atom of the donor functionalised alcohol in **7**, the –N*Me*_2_ group on the thioureide ligand can manoeuvre into closer proximity of the zinc centre to have an interaction. This coordination has a clear broadening effect on the proton signals of the methyl group (Fig. 4). This coordination is corroborated by the observation of two signals for each of the proton environments on the ^i^Pr group, made distinguishable by COSY NMR (Fig. 4), and also two distinctly different ^13^C signals for the carbon atoms in the thioureide ligand (ESI†). In **6**, only one signal was observed for each of the proton environments on the thioureide ligand indicating that this additional fluxionality is limited to **7**.

In contrast to this, reactions of **2** with HL^4^ and HL^4^* resulted in the compounds [L^1^*ZnL^4^] (**8**) and [L^1^*ZnL^4^*] (**9**) respectively, whose ^1^H NMR spectra exhibited only one set of resonances for each proton environment, indicating a single structure present in solution and therefore a lack of fluxionality (ESI†). This was confirmed *via*^1^H–^1^H 2D NMR which showed only one set of cross peaks. The signals for the donor-functionalised alcohol protons in **8** appeared broad and the thioureide ligand protons signals appeared sharp, whilst the signals for all protons in **9** appeared sharp (**9** precipitated out of the reaction mixture as a white powder as opposed to the gel like products obtained when solvent was removed from the reaction mixtures of **6**, **7** and **8**). This difference in fluxional behaviour can be explained by the change in R group from ^i^Pr to Ph on the thioureide ligand; the larger Ph group may hinder the dynamics of the complexes in solution.

#### Thermal analysis of **6** and **7**

**Fig. 5:**
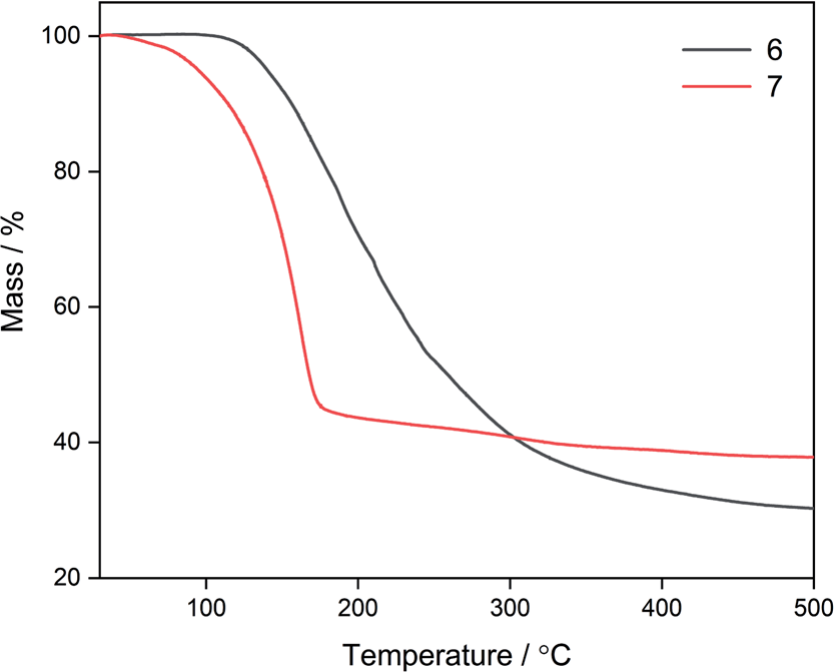
Thermal decomposition profiles for compounds **6** and **7**.

Due to the observed fluxionality in **6** and **7**, the thermal properties of these complexes were investigated *via* thermogravimetric analysis (TGA), with a view to use them as precursors in AACVD experiments. Fluxionality leads to lower decomposition temperatures as molecules are more likely to break apart during the deposition process, making them more favourable and efficient as precursors. Both complexes had single step decomposition pathways but differing decomposition profiles (Fig. 5). Compound **6** had an onset decomposition temperature of ∼120 °C, and a broad temperature window over which mass loss occurred (120–320 °C). The % residual mass (30.1%) was commensurate with the mass expected for ZnS. Compound **7** had a considerably lower onset decomposition temperature (<100 °C) than **6** and a sharp mass loss over a much narrower temperature window (100–180 °C) with no significant mass loss after ∼180 °C. The % residual mass loss (38.0%) was higher than that expected for the ternary material Zn(O,S) (ZnO_*x*_S_1−*x*_). This stark difference in the thermal profiles of the two complexes corroborated NMR data which showed the greater fluxionality present in **7**. Even though the mass loss window for the decomposition of **6** was broader, the onset decomposition temperature was still relatively low and therefore this was also considered as a precursor in AACVD experiments.

### AACVD experiments

Due to the fluxionality and low onset decomposition temperature of **6** and **7**, these molecules were investigated as AACVD precursors towards deposition of the ternary material zinc oxysulfide. Due to the excellent solubility of **6** and **7** in toluene, this was chosen as the solvent, with an optimal deposition temperature of 400 °C being chosen. Deposition conditions are shown in Table 3. Single source AACVD experiments using **6** did not lead to the target material Zn(O,S) but single source AACVD of **7** led to the deposition of the heterodichalcogenide material Zn(O,S) at 400 °C. The film was transparent, showed good coverage across the glass substrate and was well adhered; passing the Scotch tape test and was not scratched by a steel stylus.

**Table 3:** AACVD conditions of deposited films

Film	Precursor	Temperature/°C
**A**	**6**	400
**B**	**6** + excess HL^4^	400
**C**	**7**	400

**Fig. 6:**
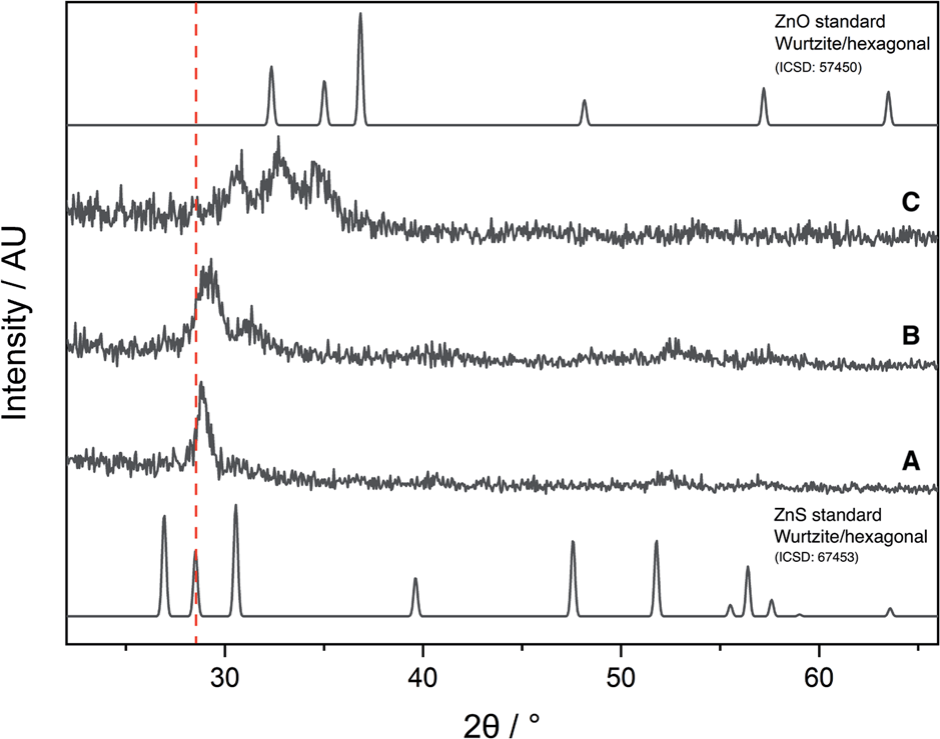
XRD patterns of films **A**, **B** and **C**, and standard ZnO and ZnS patterns.

The AACVD reaction of **6** led to deposition of film **A**, which was transparent and colourless, with an XRD pattern resembling that of cubic zinc sulfide (Fig. 6). However, the peaks were broader than would be expected for a crystalline material, indicating the amorphous nature of the film. There was also a slight shift to a higher 2*θ* angle in the peak at 29° as compared to the ZnS standard XRD pattern, due to a small amount of oxygen incorporation into the film. Due to the lack of sufficient oxygen incorporation in **A**, the AACVD reaction of **6** with excess HL^4^ (to act as an external oxygen source) was carried out. Resultant film **B** had a broader XRD pattern than **A**, with a distinct shift to a higher 2*θ* angle and broadening in the peak at ∼29° suggesting that some oxygen incorporation into the film had occurred. This was also accompanied by a slight yellow tinge to the film, further confirming the deposition of the oxysulfide material. However, these XRD patterns were not similar to those reported for zinc oxysulfide in the literature.

The AACVD reaction of **7** led to deposition of film **C**. The X-ray diffraction pattern of film **C** was markedly different to **A** and **B**, showing the characteristic broad peaks associated with Zn(O,S) at 30.9°, 32.8° and 34.5° 2*θ* indexed to the (100), (002) and (101) planes respectively, with a clear resemblance to the XRD patterns of hexagonal zinc oxide and zinc sulfide, confirming formation of the ternary material zinc oxysulfide (Fig. 5). This was consistent with reports from the literature for zinc oxysulfide films.^26,41,42^

Film **C** was visibly yellow in colour. X-ray photoelectron spectroscopy confirmed the presence of zinc, sulfur and oxygen in the film (ESI†). A single Zn environment was present in both the surface scan and the 300 s etch, suggesting that Zn(O,S) had indeed formed, and not a mixture of the binary materials ZnO and ZnS. The Zn 2p_3/2_ and 2p_1/2_ states appeared at 1044.2 and 1021.2 eV respectively and one S environment was present, with 2p_3/2_ and 2p_1/2_ states appearing at 162.8 and 161.5 eV respectively, in accordance to previous reports from the literature for Zn(O,S) films.^26^ One oxygen environment appeared at 529.5 eV for the 1s state, whilst a hydroxide state also appeared at 531.1 eV. There was however a change in binding energies for the surface scan as compared to the 300 s etch. Binding energies for both Zn and S moved to higher energies by 0.7 and 0.05 eV respectively. Furthermore, only one oxygen environment was present in the surface scan which corresponded to surface hydroxides, and the oxysulfide peak seen in the 300 s etch scan was absent. From elemental ratios, it was found that the S/Zn ratio on the surface was 1.73, indicating a sulfur rich environment as evidenced by absence of an oxygen environment, as well as a shift towards higher binding energies, whilst the S/Zn ratio for the bulk material (300 s etch) was calculated to be 0.40, in line with observed results. Previous work has shown that for zinc oxysulfide films produced *via* AACVD which have a S/Zn ratio above 0.13,^26^ Hall effect data cannot be acquired due to the high resistivities of the films. The as-deposited films presented here have a high S/Zn ratio on the surface, therefore electronic data could not be acquired for film **C**. It has been previously reported that the elongated grain structure exhibited in ZnO films is reduced upon inclusion of S,^42^ and whilst exchange of O with S in the lattice sites of Zn(O,S) alters conductivity, there is a point at which lattice sites predominantly occupied by S will result in films that are not conductive. Different deposition and annealing conditions can lead to variations of the surface morphology and therefore properties, which is why using a buffer layer of variable composition such that the interface can be easily optimised is beneficial. Therefore S/Zn ratios should be tuned relative to the materials they will be combined with, to achieve the optimal electronic properties for the relevant device.

**Fig. 7:**
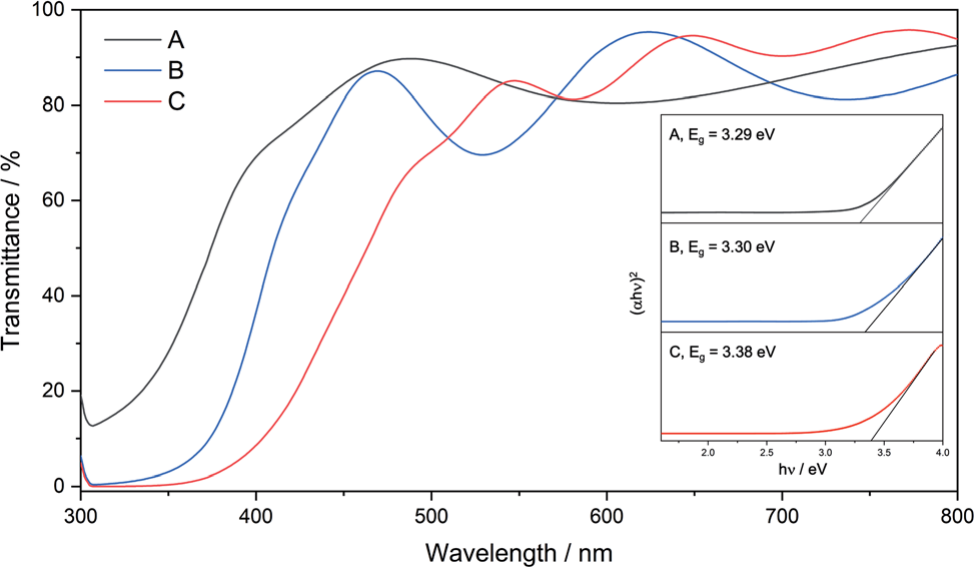
UV/vis transmittance spectra of films **A**, **B** and **C**. Inset: Tauc plots of films **A**, **B** and **C** showing their optical band gap energies.

All films were transparent and had good optical light transmission (>70%) in the range 500–800 nm (Fig. 7). Film **C** was visibly yellow in colour and as such had absorption at higher wavelengths, followed by film **B** which was slightly yellow in colour and film **A** which was colourless, Raman spectra are included in the ESI.† UV/vis spectroscopy was also used to estimate the optical band gaps of the films using Tauc plots (Fig. 7). The absorbance edge in the UV/vis spectra for **A–C** varied by 100 nm, resulting in an estimated band gap of 3.38 eV for **C**, intermediate to that of zinc sulfide (3.91 eV (hexagonal) and 3.54 eV (cubic)) and wurtzitic zinc oxide (∼3.3 eV). However, although the transmittance spectra for the three films look starkly different, the band gaps for the films were estimated to be close together. Interestingly, it would be expected that films with a higher absorbance at longer wavelengths (*e.g.*, **C**) would have a lower band gap, but this was not the case. Previous work from our group showed that zinc oxysulfide films with similar XRD patterns to that of film **C** exhibited much higher band gaps (3.74–3.93 eV). This was due to the precursor used depositing pure phase hexagonal zinc sulfide without an external oxygen source. Depositions using **6** led to deposition of zinc sulfide with a level of incorporation of oxygen and due to this, and the well-known band bending effects of alloys, it would not be surprising for the band gaps of these materials to occur at lower energies.^43^

**Fig. 8:**
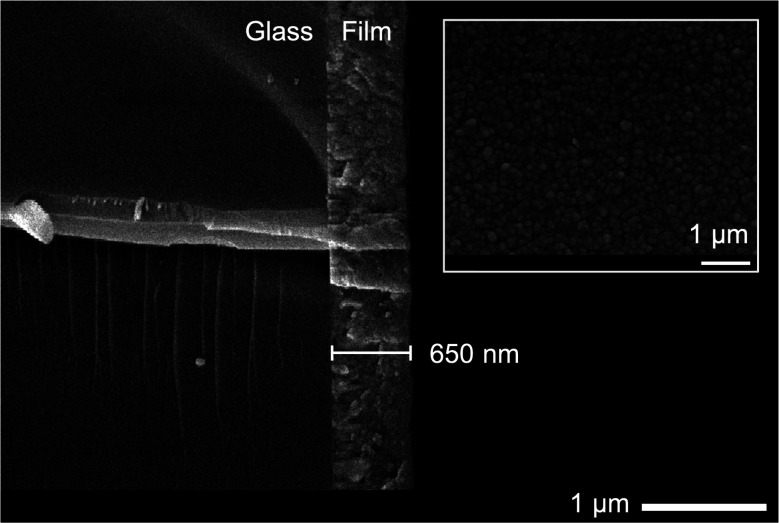
SEM of film **C**: cross section SEM at ×23 000 showing film thickness of 650 nm, inset: plane view at ×19 000.

SEM images revealed that film **C** was made up of well-connected particulates of similar size (200–300 nm), and that the film thickness was consistent across the film at around 650 nm (Fig. 8). Images of films **A** and **B** showed smaller particulates (<100 nm), consistent with the reduced peak broadening from XRD data (ESI†).

Energy dispersive X-ray (EDX) analysis was used to confirm the presence of zinc, sulfur and oxygen (where applicable) in the films. However, EDX penetrates to depths of about 2–3 μm and therefore oxygen from the glass substrate was also accounted for in the total atomic percentages, as the film thicknesses are <1 μm. Therefore, it is useful to compare the relative sulfur content in the film as we would expect films **A** and **B** to have a higher relative sulfur content as compared to film **C**. Film **C** had the lowest S/Zn ratio of 0.3 as expected, due to a larger proportion of oxygen present in the film, evidencing zinc oxysulfide. This corroborated the elemental ratio calculated from XPS, which was slightly higher (S/Zn = 0.4) due to the acquisition of data closer to the surface, where the film was sulfur rich.

## Conclusions

In this work, the formation of heteroleptic zinc complexes *via* systematic variation of ligand type and investigations of dynamic properties of the resultant precursors have culminated in the deposition of the heterodichalcogenide material Zn(O,S) *via* the single source AACVD reaction of the heteroleptic alkoxyzinc thioureide complex **7** at 400 °C, without the use of an external zinc or oxygen source. This is the first example of the deposition of Zn(O,S) *via* a single source route. We have upheld our hypothesis that using a precursor with two different ligands – one with an oxygen donor atom and the second with a sulfur donor atom, has facilitated the deposition of a ternary material.

A set of ethyl (**1**, **2**), amide (**3**), BDI (**5**) and alkoxyzinc (**6**, **7**, **8**, **9**) thioureides have been synthesised and characterised, with compounds **5**, **6** and **7** demonstrating dynamic behaviour in solution. Thorough variable temperature NMR investigations revealed a two-fold fluxionality in **7** due to contributions from both ligands which was attributed to the smaller methyl substituents present on the donor functionalised alcohol. This was firmly supported by TGA, which revealed that **7** had a significantly lower onset decomposition temperature than **6**. This was corroborated through AACVD experiments where the target heterodichalcogenide material Zn(O,S) was deposited from the single source reaction of **7**, but not from **6**.

We have shown a clear correlation between fluxionality and decomposition temperature. As stability and decomposition temperature of precursors are directly proportional, molecules that are to function as SSPs need to be designed so as to be stable enough to be isolated and be easily handled, but to be unstable enough to allow for a lower decomposition temperature. The instability of reagents such as ZnEt_2_, AlMe_3_ and GaMe_3_ results from the presence of metal carbon bonds which makes these compounds pyrophoric and therefore difficult to handle. The instability resulting from molecules that exhibit solution state fluxionality does not make these compounds highly reactive, and so molecules that exhibit dynamic behaviour could serve as safer alternatives to pyrophoric species in solution state deposition methods.

## Author contributions

CEK and MAB proposed the study. KLM conducted preliminary ligand studies, all further synthesis and analysis were conducted by MAB. CEK and CJC supervised the work. MAB and CEK wrote the manuscript. All authors discussed and commented on the manuscript.

## Conflicts of interest

There are no conflicts to declare.

## Supplementary Material

SC-012-D1SC01846A-s001

SC-012-D1SC01846A-s002
