# Supplementary material for: Synthesis, solution dynamics and chemical vapour deposition of heteroleptic zinc complexes via ethyl and amide zinc thioureides
Source: Chem Sci. 2021 May 24;12(25):8822–31. doi: 10.1039/d1sc01846a (PMC8246097; doi:10.1039/d1sc01846a)
Supplement: SC-012-D1SC01846A-s001 [file SC-012-D1SC01846A-s001.pdf]

Electronic Supplementary Material (ESI) for Chemical Science.
This journal is © The Royal Society of Chemistry 2021

Electronic Supporting information for:

Synthesis, solution dynamics and chemical vapour deposition of heteroleptic zinc complexes via ethyl and amide zinc thioureides

*Malavika A. Bhide, Kristian L. Mears, Claire J. Carmalt and Caroline E. Knapp**

Materials Chemistry Centre, Department of Chemistry, University College London, 20 Gordon Street, London WC1H 0AJ, UK.

Corresponding author email address: caroline.knapp@ucl.ac.uk

Table of contents

A. Synthetic procedures 2
B. NMR spectral data 7
C. Crystallographic data 26
D. SEM images 38
E. XPS spectra 39
F. Raman 40

A. Synthetic procedures

General procedures

All preparations were performed under an inert argon atmosphere using standard Schlenk techniques or using an MBraun nitrogen-filled glovebox. All chemicals were obtained from commercial sources. All solvents were obtained from a solvent purification system and stored over molecular sieves. C_6D_6, HL^4 and HL^{4*} were dried using freeze-pump-thaw cycles and stored over molecular sieves. Zinc bis[bis(trimethylsilyl)amide] was prepared according to literature procedures,[44] purified *via* vacuum distillation and stored in a glovebox. Syntheses of HL^2, HL^3 and HL^{3*} are detailed in the ESI.

Single crystal X-ray diffraction (XRD) data were collected using a SuperNova Atlas (Dual) diffractometer using Cu K_α radiation of wavelength 1.54184 Å. Suitable crystals were selected and mounted on a nylon loop and the crystal was kept at 150 K during data collection. Nuclear magnetic resonance (NMR) data were recorded in C_6D_6 solutions using a Bruker Advance III 300 or 500 MHz instrument at ambient temperature unless stated otherwise. ^1H and $^{13}C\{^1H\}$ NMR assignments were confirmed by ^1H–^1H (COSY and NOESY) and ^1H–^{13}C (HSQC and HMBC) experiments where necessary. Thermogravimetric analysis (TGA) measurements were made using a PerkinElmer STA6000 TGA instrument, with a sensitivity of 0.1 mg and used N_2 as the shield gas. The samples were heated from 30 °C to 500 °C, at a heating rate of 10 °C min^{-1} under flow of shield gas.

Synthesis

Synthesis of HL^2. Isopropylamine (2.9642 g, 0.05 mol) and acetylacetone (5.0076 g, 0.05 mol) were combined in toluene (20 cm^3) and the mixture was stirred and heated to reflux overnight, resulting in a transparent orange liquid. The solvent and water were removed *in vacuo* to yield **HL^2** as an orange oil (yield = 5.8564 g, 81%). ^1H NMR δ/ppm (C_6D_6, 300 MHz): 0.79 (6H, d, $CH(CH_3)_2$), 1.45 (3H, s, $HNCCH_3$), 2.03 (3H, s, $COCH_3$), 3.10 (1H, m, NHC*H*), 4.85 (1H, s, COC*H*), 11.18 (1H, s, N*H*).

Synthesis of HL^3. 2,6-diisopropylaniline (18.8 cm^3, 0.1 mol), acetylacetone (5.0024 g, 0.05 mol) and concentrated HCl (37%, 12 M, 4.16 cm^3, 0.05 mol) were combined in ethanol (100 cm^3) and the mixture was heated to reflux for 3 days. Ethanol and water were removed in vacuo from the reaction mixture yielding a light brown solid. Saturated Na_2CO_3 solution and DCM were added and the product was extracted into the organic phase. The aqueous layers were further washed with DCM. The combined organic layers were dried over sodium sulfate, filtered and the solvent was removed in vacuo to yield a brown oil. Hot methanol was added, and the solution was kept at room temperature overnight for crystallization before being filtered to yield pale yellow crystals of **HL^3**. The mother liquor was subject to further recrystallisation. The reported yields are the combined yields from the recrystallisation process (yield = 8.58 g).

^{1}H NMR δ/ppm (C_6D_6, 300 MHz): 1.17 (12H, d, Ar-C(CH_3)$_2$), 1.22 (12H, d, Ar-C(CH_3)$_2$), 1.67 (6H, s, NCCH_3), 3.32 (4H, m, Ar-C*H*), 4.89 (1H, s, NCC*H*), 7.10–7.22 (6H, Ar-*H*) 12.47 (1H, s, N*H*).

Synthesis of HL^{3*}. HL^{3*} was synthesised in an analogous method to **HL^3**, with the following reagent amounts: 2,6-diethylaniline (14.9 g, 0.1 mol), acetylacetone (5.0167 g, 0.05 mol) and concentrated HCl (37%, 12 M, 4.16 cm^3, 0.05 mol). ^{1}H NMR δ/ppm (C_6D_6, 300 MHz): 1.16 (12H, t, Ar-(CH_2CH_3)$_2$), 1.60 (6H, s, NCCH_3), 2.52 (4H, m, Ar-(C$H_2$$CH_3$)), 2.65 (4H, m, Ar-(C$H_2$$CH_3$)), 4.84 (1H, s, NCC*H*), 7.05–7.10 (6H, Ar-*H*), 12.34 (1H, s, N*H*).

Synthesis of ^{i}PrN(H)CSNMe$_2$ (HL^1). Isopropyl isothiocyanate (4.2327 g, 30 mmol) was added to a solution of dimethylamine (2 M in THF) (15 cm^3, 30 mmol) in hexane (20 cm^3) and stirred for 30 min. The solvent was removed *in vacuo* to yield **HL^1** as a white powder. **HL^1** was recrystallised from toluene as white needles (yield = 3.29 g, 75%). ^{1}H NMR δ/ppm (C_6D_6, 500 MHz): 1.04 (6H, d, J = 6.5 Hz, CH(CH_3)$_2$), 2.55 (6H, s, N(CH_3)$_2$), 4.46 (1H, s, N*H*), 4.87 (1H, m, C*H*). ^{13}C{^{1}H} NMR δ/ppm (C_6D_6, 500 MHz): 22.9 (CH(*C*H_3)$_2$), 39.4 (N*Me*$_2$), 47.3 (*C*H(CH_3)$_2$), 182.1 (*C*=S). Elemental anal. calc.%: C: 49.30, H: 9.70, N: 19.20, found%: C: 49.32, H: 9.69, N: 18.84.

Synthesis of PhN(H)CSNMe$_2$ (HL^{1*}). Phenyl isothiocyanate (2.7028 g, 20 mmol) was added to a solution of dimethylamine (2 M in THF) (10 cm^3, 20 mmol) in hexane (20 cm^3). A white precipitate formed immediately, and the mixture was filtered to yield **HL^{1*}** as a white microcrystalline solid. **HL^{1*}** was recrystallised from a toluene/THF mixture as white needles (yield = 2.36 g, 74%), with crystals of a suitable quality for analysis by single crystal XRD. ^{1}H NMR δ/ppm (C_6D_6, 500 MHz): 2.53 (6H, s, N(CH_3)$_2$), 6.40 (1H, s, N*H*), 6.92 (1H, tt, J = 7.3, 1.1 Hz, *para*-C*H*), 7.09 (2H, t, J = 8.0 Hz, *meta*-C*H*), 7.17 (2H, d, J = 15.0 Hz, *ortho*-C*H*). ^{13}C{^{1}H} NMR δ/ppm (C_6D_6, 500 MHz): 40.7 (N*Me*$_2$), 124.3 (Ar-*C*), 124.8 (Ar-*C*), 128.8 (Ar-*C*), 140.9 (Ar-*C*), 183.2 (*C*=S). Elemental anal. calc.%: C: 60.00, H: 6.70, N: 15.50, found%: C: 60.16, H: 6.74, N: 15.36.

Synthesis of [L^{1}ZnEt]$_2$ (1). A solution of **HL^1** (1.022 g, 7 mmol) in toluene (10 cm^3) was added to a cooled solution of diethylzinc (1.1 M in toluene) (6.3 cm^3, 7 mmol) in toluene (10 cm^3) and stirred overnight. Visible production of ethane gas was observed. The solvent was removed *in vacuo* to yield the white powder **1** (yield = 1.27 g, 76%). **1** was redissolved in a mixture of toluene and hexane and left in the freezer for 3 days after which colourless crystals suitable for analysis via single crystal XRD had formed. ^{1}H NMR δ/ppm (C_6D_6, 500 MHz): 0.82 (2H, q, J = 8.1 Hz, Zn-CH_2), 1.04 (6H, d, J = 6.2 Hz, CH(CH_3)$_2$), 1.77 (3H, t, J = 8.1 Hz, Zn-CH_2CH_3), 2.59 (6H, s, N(CH_3)$_2$), 3.51 (1H, hept, J = 6.1 Hz, NC*H*). ^{13}C{^{1}H} NMR δ/ppm (C_6D_6,

500 MHz): 2.4 (Zn-*C*H_2), 13.4 (Zn-CH_2*C*H_3), 25.1 (CH(C*H_3*)$_2$), 42.9 (N*Me_2*), 49.6 (N*C*H), 172.5 (N*C*S).

Synthesis of [L[1*]ZnEt]$_2$ (2). A solution of **HL[1*]** (1.08 g, 6 mmol) in toluene (10 cm^3) was added to a cooled solution of diethylzinc (1.1 M in toluene) (5.4 cm^3, 6 mmol) in toluene (10 cm^3) and stirred overnight. Visible production of ethane gas was observed. The solvent was removed *in vacuo* to yield the white powder **2** (yield = 1.10 g, 67%). Slow evaporation of **2** from a C_6D_6 solution afforded colourless crystals suitable for analysis via single crystal XRD. ^{1}H NMR *δ*/ppm (C_6D_6, 500 MHz): 0.93 (2H, q, *J* = 8.1 Hz, Zn-C*H_2*), 1.77 (3H, t, *J* = 8.1 Hz, Zn-C*H_2*C*H_3*), 2.35 (6H, s, N(C*H_3*)$_2$), 6.77 (1H, t, *J* = 7.4 Hz, *para*-C*H*), 6.88 (2H, d, *J* = 7.7 Hz, *ortho*-C*H*), 7.00 (2H, t, *J* = 7.7 Hz, *meta*-C*H*). ^{13}C{^{1}H} NMR δ/ppm (C_6D_6, 500 MHz): 1.7 (Zn-*C*H_2), 13.6 (Zn-CH_2*C*H_3), 42.9 (N*Me_2*), 122.7 (*ortho-C*H), 123.1 (*para-C*H), 129.2 (*meta-C*H), 148.2 (N*C*), 173.2 (N*C*S). Elemental anal. calc.%: C: 48.30, H: 5.90, N: 10.20, found%: C: 47.71, H: 5.80, N: 9.98.

Synthesis of [L[1]Zn(N(SiMe$_3$)$_2$)]$_2$ (3). A solution of **HL[1]** (0.1816 g, 1.25 mmol) in toluene was added to a cooled solution of [Zn(N(SiMe$_3$)$_2$)$_2$] (0.4802 g, 1.25 mmol) in toluene and stirred overnight. The solvent was removed *in vacuo* to yield a white powder (yield = 0.27 g, 58 %). Slow evaporation from a C_6D_6 solution afforded colourless crystals of **3** suitable for analysis via single crystal XRD. ^{1}H NMR *δ*/ppm (C_6D_6, 500 MHz): 0.46 (18H, s, N(Si(C*H_3*)$_3$)$_2$), 1.16 (6H, d, *J* = 6.5 Hz, CH(C*H_3*)$_2$), 2.64 (6H, s, N(C*H_3*)$_2$), 3.57 (1H, sept, *J* = 5.0 Hz, NC*H*). ^{13}C{^{1}H} NMR δ/ppm (C_6D_6, 500 MHz): 6.6 (N(Si*Me_3*)$_2$), 25.1 (N(CH*Me_2*)), 43.5 (N*Me_2*), 50.3 (N(*C*HMe_2)). Elemental anal. calc.%: C: 38.80, H: 8.40, N: 11.30, found%: C: 38.43, H: 8.02, N: 11.12.

Synthesis of [L[1*]ZnL[3*]] (5). To a cooled solution of 2 (0.2653 g, 0.88 mmol) in toluene was added a solution of **HL[3*]** (0.1813 g, 0.5 mmol) in toluene. The pale yellow solution was heated to 100 °C for 6 days after which a fine precipitate had formed. The solution was filtered, and the solvent was removed to yield the viscous dark yellow oil, **5**. Recrystallisation attempts were unsuccessful. ^{1}H NMR δ/ppm (C_6D_6, 500 MHz): 1.12 (6H, m, CH_2C*H_3*), 1.35 (6H, m, CH_2C*H_3*), 1.65 (6H, s, NCC*H_3*), 1.99 (6H, s, N*Me_2*), 2.41 (2H, m, C*H_2*CH_3), 2.54 (2H, m, C*H_2*CH_3), 2.83 (2H, m, C*H_2*CH_3), 3.23 (2H, m, C*H_2*CH_3), 4.88 (1H, s, NCC*H*), 6.40 – 7.15 (11H, Ar-*H*). ^{13}C{^{1}H} NMR δ/ppm (C_6D_6, 500 MHz): 13.8 (CH_2*C*H_3), 23.2 (NC*C*H_3), 24.4 (*C*$H_2$$CH_3$), 42.7 (N*$Me_2$*), 94.1 (NC*C*H), 122–138 (aryl-*C*), 146. 9 (N*C*CH_3), 168.2 (N*C*S).

Synthesis of [L[1]ZnL[4]]$_2$ (6). To a cooled solution of **1** (0.1204 g, 0.5 mmol) in toluene, was added dropwise a solution of **HL[4]** (0.0692 g, 0.5 mmol) in toluene. The resultant pale yellow solution was stirred overnight. The solution was concentrated to *ca.* 10 cm^3 and left in the freezer

for 1 month after which colourless crystals of **6** suitable for analysis via single crystal XRD had formed. ^{1}H NMR δ/ppm (C_6D_6, 500 MHz, 277 K): 0.67 (3H, t, J = 7.1 Hz, $N(CH_2CH_3)_2$), 0.76 (3H, t, J = 7.2 Hz, $N(CH_2CH_3)_2$), 1.23 (1H, m, OCH_2CH_2), 1.53 (6H, dd, J = 6.3, 3.9 Hz, $NCH(CH_3)_2$), 1.72 (1H, m, OCH_2CH_2), 2.33 (1H, m, $N(CH_2CH_3)_2$), 2.37 (2H, m, NCH_2), 2.50 (1H, m, $N(CH_2CH_3)_2$), 2.74 (1H, m, $N(CH_2)CH_3$), 3.00 (1H, m, $N(CH_2)CH_3$), 3.07 (6H, s, $N(CH_3)_2$), 3.83 (1H, m, OCH_2), 3.98 (1H, m, OCH_2), 4.54 (1H, hept, J = 6.3 Hz, NC*H*). ^{13}C{^{1}H} NMR δ/ppm (C_6D_6, 500 MHz): 7.27 ($N(CH_2)CH_3$), 8.50 ($N(CH_2)CH_3$), 25.25 ($NCH(CH_3)_2$), 29.31 (OCH_2CH_2), 41.15 ($N(CH_3)_2$), 44.75 ($N(CH_2)CH_3$), 45.86 ($N(CH_2)CH_3$), 53.60 (N*C*H), 54.92 (NCH_2), 66.94 (OCH_2), 158.64 (N*C*S). Elemental anal. calc.%: C: 45.81, H: 8.58 N: 12.33, found%: C: 45.80, H: 8.56, N: 11.94.

Synthesis of $[L^{1}ZnL^{4*}]_2$ (7). To a cooled solution of **1** (0.69 g, 3 mmol) in toluene, was added dropwise a solution of **HL^{4*}** (0.42 cm^3, 3 mmol) in toluene. The resultant pale yellow solution was stirred overnight. The solution was concentrated to *ca.* 10 cm^3 and left in the freezer for 1 week after which colourless crystals of **7** suitable for analysis via single crystal XRD had formed. ^{1}H NMR δ/ppm (C_6D_6, 500 MHz, 283 K): 1.10 (2H, m, OCH_2CH_2), 1.46–1.57 (12H, m, $NCH(CH_3)_2$), 1.75 (2H, m, OCH_2CH_2), 1.91 (2H, s, NCH2), 1.98 (1H, m, NCH_2), 2.03–2.34 (12H, m, Zn-N*Me*$_2$), 2.73 (1H, m, NCH_2), 3.04 (6H, s, N*Me*$_2$), 3.15 (6H, br, N*Me*$_2$), 3.80–4.14 (4H, m, OCH_2), 4.54–4.60 (2H, m, NC*H*). ^{13}C{^{1}H} NMR δ /ppm (C_6D_6, 500 MHz): 25.2 ($NCH(CH_3)_2$), 25.6 ($NCH(CH_3)_2$), 29.8 (OCH_2CH_2), 30.3 (OCH_2CH_2), 41.2 (NMe2), 41.2 (NMe2), 44.3–49.0 (Zn-N*Me*$_2$), 53.4 (N*C*H), 53.7 (N*C*H), 61.7-62.1 (NCH_2), 67.3–68.7 (OCH_2), 158.9 (N*C*S). Elemental anal. calc.%: C: 42.24, H: 8.06, N: 13.43, found%: C: 41.05, H: 7.65, N: 13.02.

Synthesis of $[L^{1*}ZnL^{4}]$ (8). To a cooled solution of 2 (1.08 g, 4.5 mmol) in toluene was added dropwise a solution of **HL^4** (0.53 cm^3, 4.5 mmol) in toluene. The solution was stirred overnight, and the solvent was removed to yield the viscous pale yellow substance, **8**. Recrystallisation attempts were unsuccessful. ^{1}H NMR δ/ppm (C_6D_6, 500 MHz): 0.87 (6H, t, J = 7.2 Hz, $N(CH_2CH_3)_2$), 1.52 (2H, br, OCH_2CH_2), 2.39 (2H, br, NCH_2), 2.74 (4H, br, $N(CH_2CH_3)_2$), 2.90 (6H, s, N*Me*$_2$), 4.13 (1H, br, OCH_2), 6.88–7.27 (5H, m, Ar-*H*). ^{13}C{^{1}H} NMR δ/ppm (C_6D_6, 500 MHz): 8.8 ($N(CH_2CH_3)_2$), 29.9 (OCH_2CH_2), 42.6 (N*Me*$_2$), 47.4 ($N(CH_2CH_3)_2$), 55.1 (NCH_2), 66.3 (OCH_2), 121.0 (Ar-*C*), 123.5 (Ar-*C*), 124.0 (Ar-*C*), 128.7 (Ar-*C*), 151.7 (N*C*S). Elemental anal. calc.%: C: 51.27, H: 7.26, N: 11.21, found%: C: 51.56, H: 7.23, N: 10.81.

Synthesis of $[L^{1*}ZnL^{4*}]$ (9). To a cooled solution of **2** (1.44 g, 5.2 mmol) in toluene, was added dropwise a solution of **HL^{4*}** (0.62 cm^3, 5.2 mmol) in toluene. After warming to room temperature, a fine white precipitate formed which was heated back into solution. Repeated attempts to recrystallise the solid resulted in the formation of a microcrystalline powder. The

solvent was removed to yield the white solid **9**. ^{1}H NMR δ/ppm (C_6D_6, 500 MHz): 1.39 (2H, p, J = 5.1 Hz, (OCH_2CH_2), 2.23 (6H, s, Zn-N(CH_3)$_2$), 2.25 (2H, m, NCH_2), 2.81 (6H, s, N(CH_3)$_2$), 4.21 (2H, t, J = 5.3 Hz, OCH_2), 6.86 (1H, tt, J = 7.2, 1.3 Hz, *para*-C*H*), 7.15 (2H, d, J = 7.3 Hz, *ortho*-C*H*), 7.22 (2H, m, *meta*-C*H*). ^{13}C{^{1}H} NMR δ/ppm (C_6D_6, 500 MHz): 30.7 (OCH_2CH_2), 43.2 (N(CH_3)$_2$), 47.1 (Zn-N(CH_3)$_2$), 62.3 (NCH_2), 66.8 (OCH_2), 120.9 (*para*-*C*H), 123.2 (*meta*-*C*H), 128.9 (*ortho*-*C*H), 151.0 (N*C*), 161.2 (N*C*S). Elemental anal. calc.%: C: 48.49, H: 6.68, N: 12.12, found%: C: 47.88, H: 6.60, N: 11.65.

AACVD

Depositions were carried out under argon (99.99% from BOC). Precursor solutions were placed in an AACVD glass bubbler and generation of the aerosol mist was achieved using an ultrasonic humidifier containing a piezoelectric device. The aerosol was transported in a flow of argon gas, through a brass baffle to a horizontal bed, cold-wall reactor fitted with a graphite block containing a Whatman cartridge heater, controlled using a Pt–Rh thermocouple (as described previously).[45] A top plate was suspended 0.5 cm above the substrate to ensure a laminar flow. The glass substrates used (15 cm ´ 5 cm ´ 0.3 cm) were cut from SiO_2, pre-coated (ca. 50 nm thick SiO_2 barrier layer) standard float glass (Pilkington NSG). Before starting depositions, the reactor and substrate were heated to the required temperature under a flow of argon gas and were left to equilibrate for ca. 10 min. After the depositions were complete, the substrate was allowed to cool to room temperature under a flow of argon gas to maintain an inert atmosphere. Films were handled and stored in air.

AACVD. Solutions of **6** (3.2 mmol) and **7** (2.5 mmol) in toluene were prepared as detailed above and transferred to an AACVD bubbler. AACVD reactions were carried out at 400 °C at a flow rate of 0.7 L min^{-1}. For deposition of film **B**, HL^4 (1.0 cm^3, 10 mmol) was added to the solution of **6** and stirred for 30 min before starting the deposition.

Physical measurements

Analysis of thin films were carried out as deposited. Grazing incident (GI)XRD patterns were recorded using a Bruker D8 Discover diffractometer. X-ray photoelectron spectroscopy (XPS) measurements were carried out on a Thermo Scientific K_α photoelectron spectrometer with a monochromatic Al K_α source. Data were calibrated against C(1s) adventitious carbon (284.6 eV) for charge correction and peaks were modelled using CasaXPS software. UV/vis transmittance spectra were produced using a Shimadzu UV-2600 spectrophotometer using an air background and recorded between 300–800 nm. Energy dispersive X-ray (EDX) analysis and scanning electron microscope (SEM) images were obtained using a JEOL JSM-7600F field emission SEM instrument on gold coated samples with an accelerating voltage of either 5 or 10 kV.

B. NMR spectral data

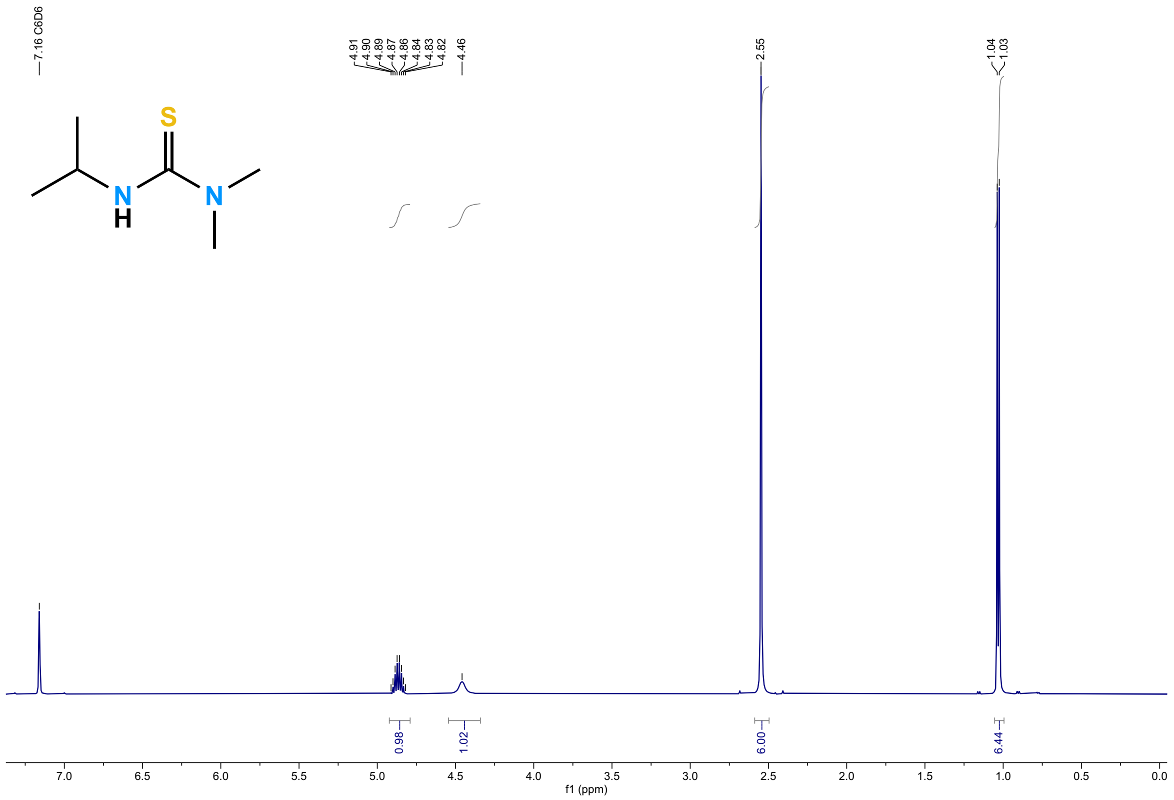

Figure S1: ^{1}H NMR spectrum of HL^1 in C_6D_6.

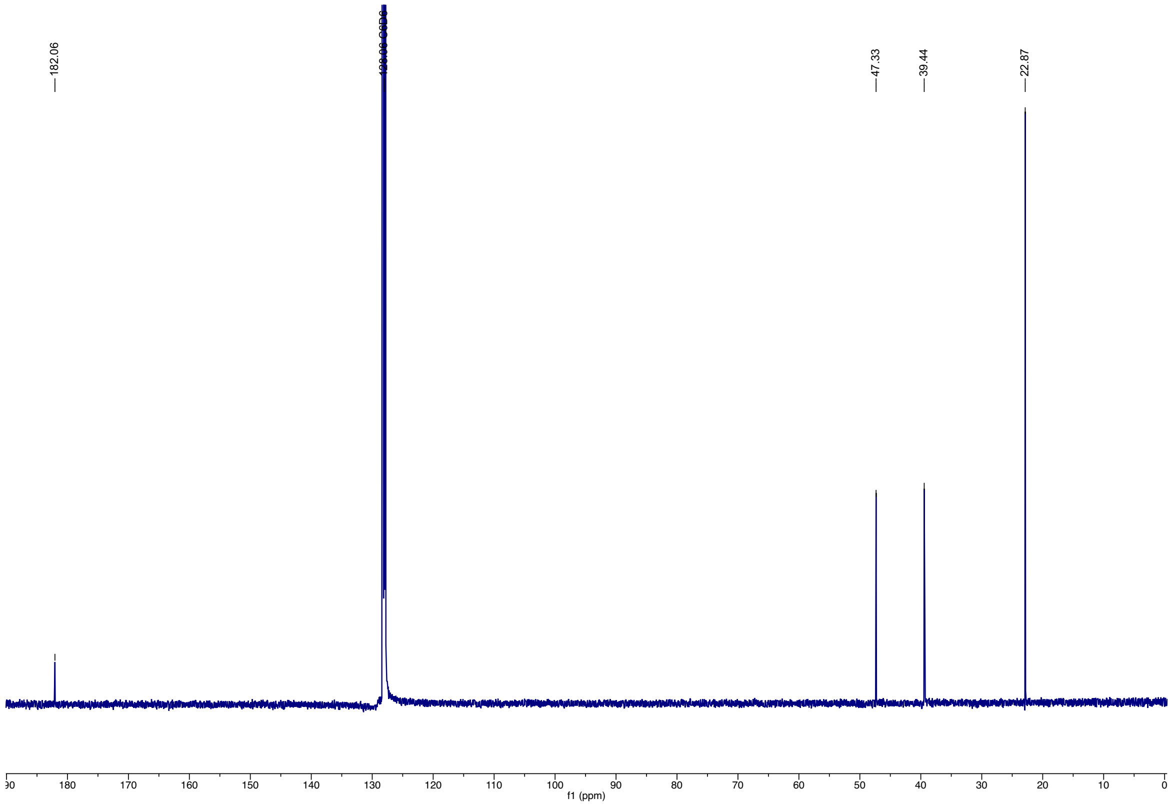

Figure S2: ^{13}C NMR spectrum of HL^1 in C_6D_6.

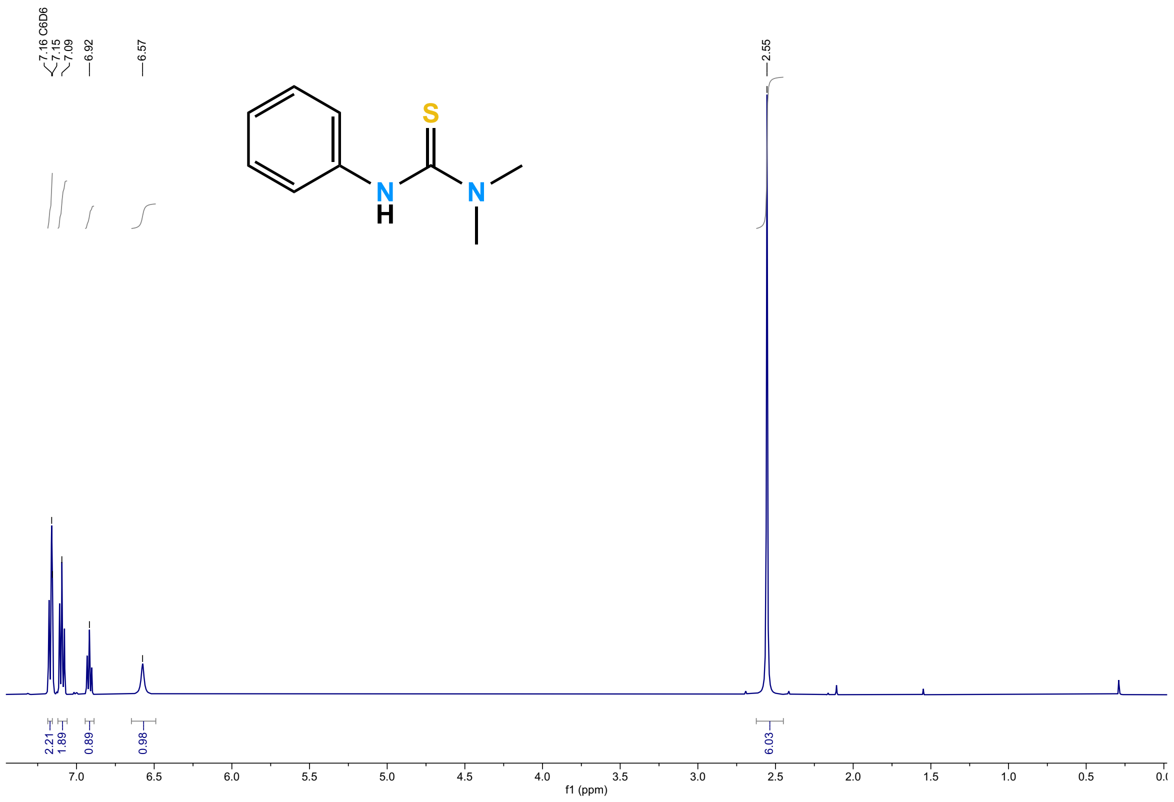

Figure S3: ^{1}H NMR spectrum of HL^{1*} in C_6D_6.

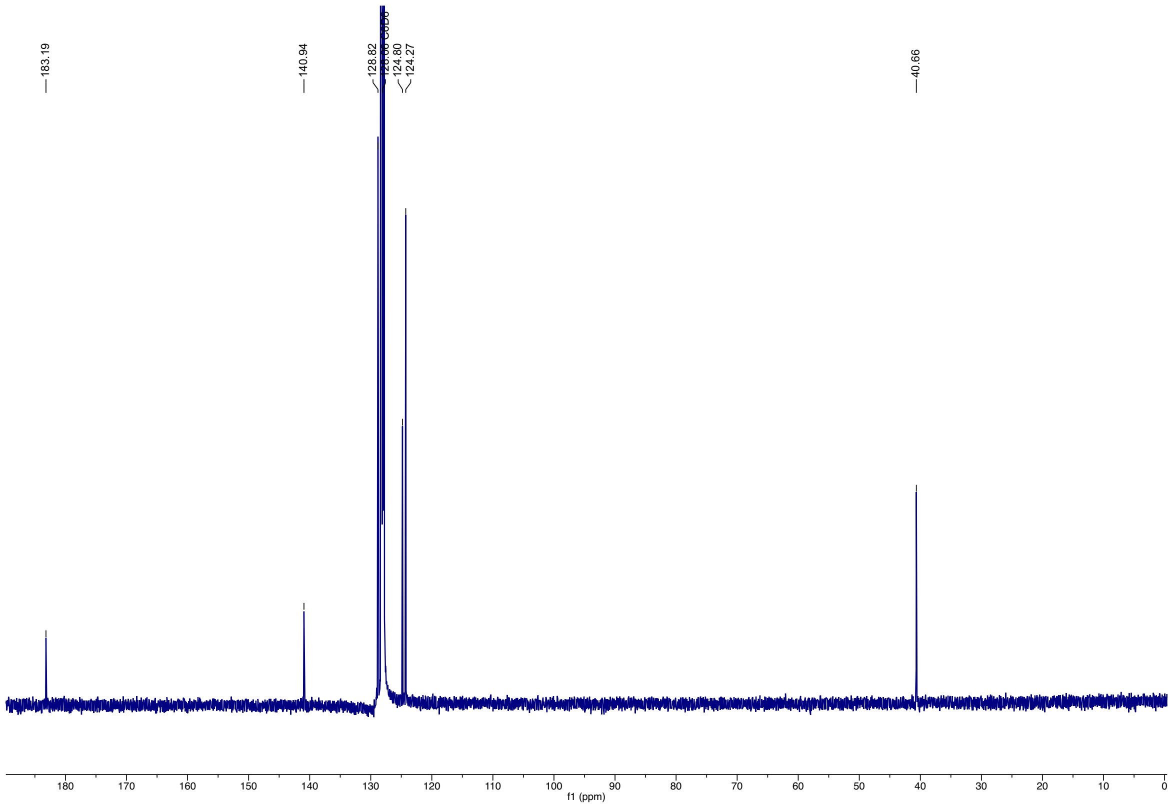

Figure S4: ^{13}C NMR spectrum of HL^{1*} in C_6D_6.

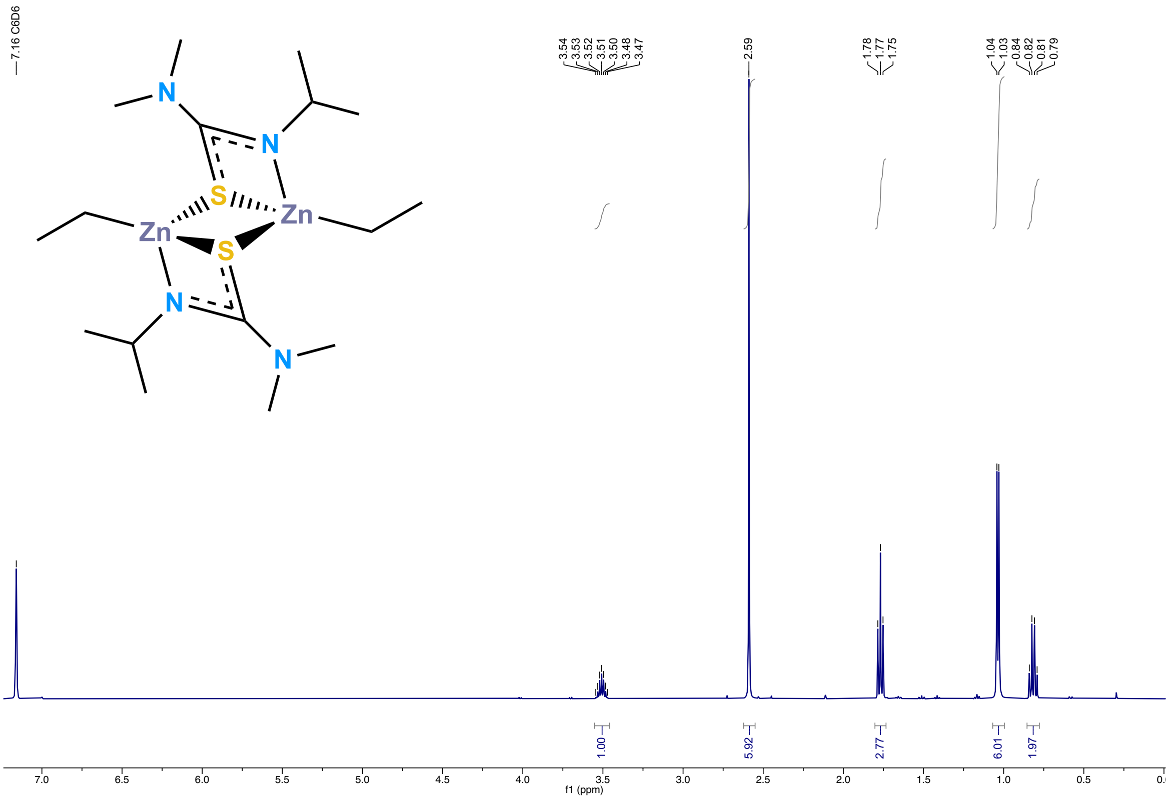

Figure S5: ^{1}H NMR spectrum of **1** in C_6D_6.

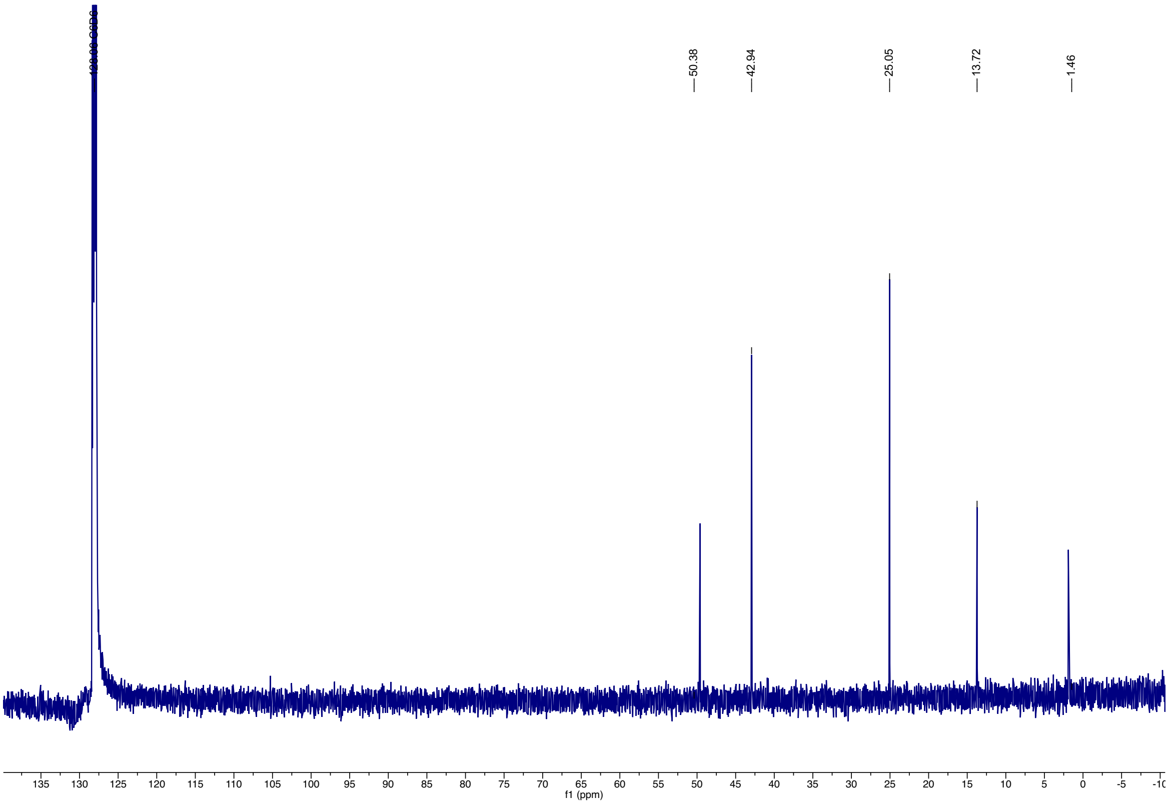

Figure S6: ^{13}C NMR spectrum of **1** in C_6D_6.

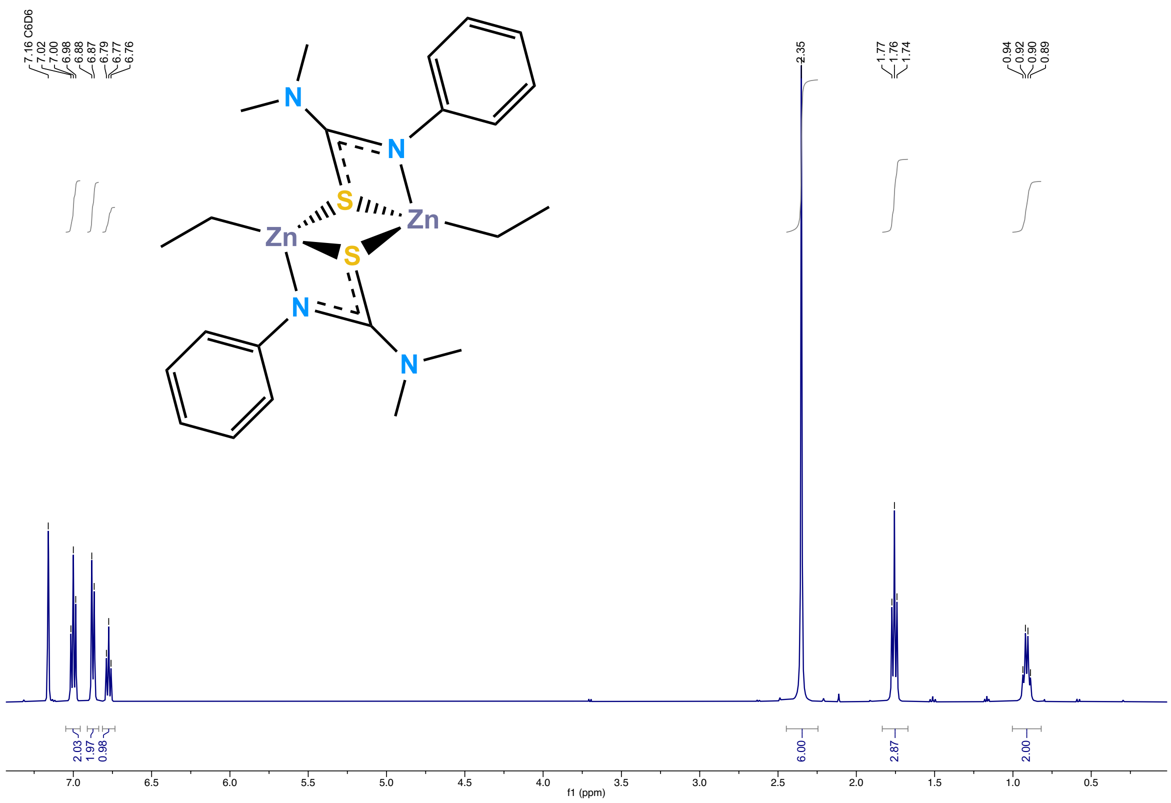

Figure S7: ^{1}H NMR spectrum of **2** in C_6D_6.

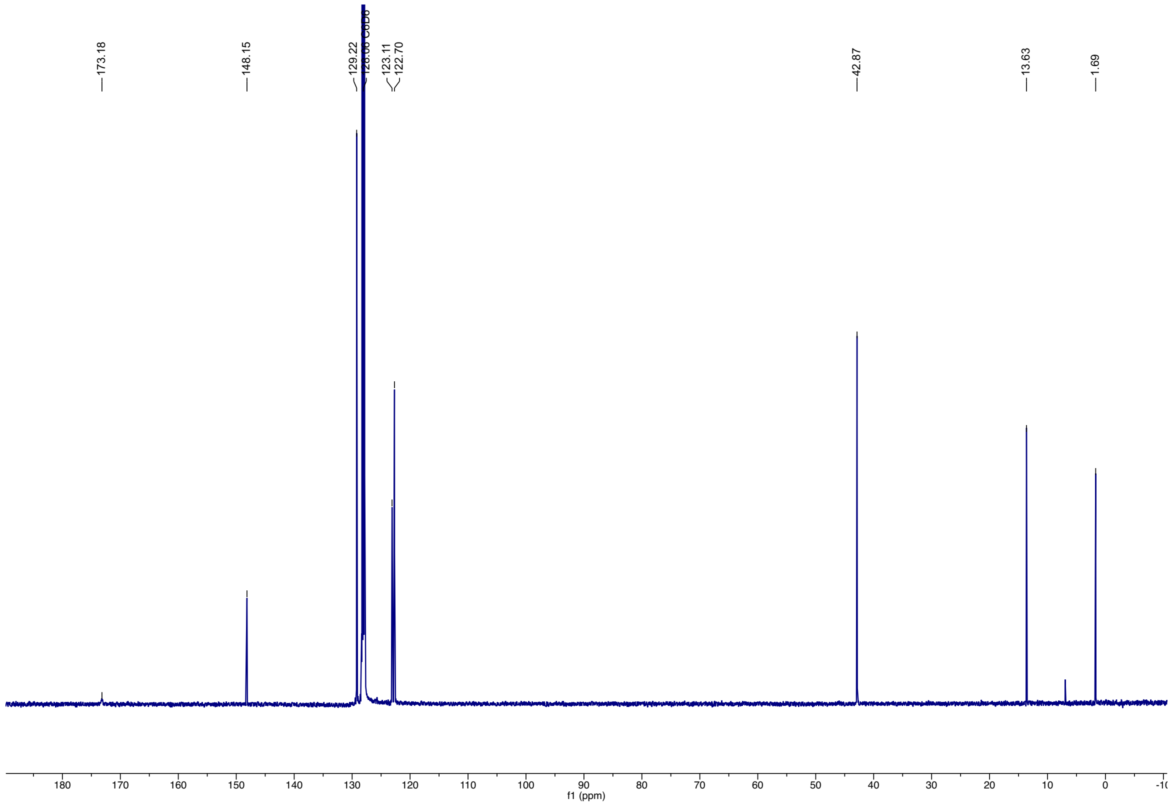

Figure S8: ^{13}C NMR spectrum of **2** in C_6D_6.

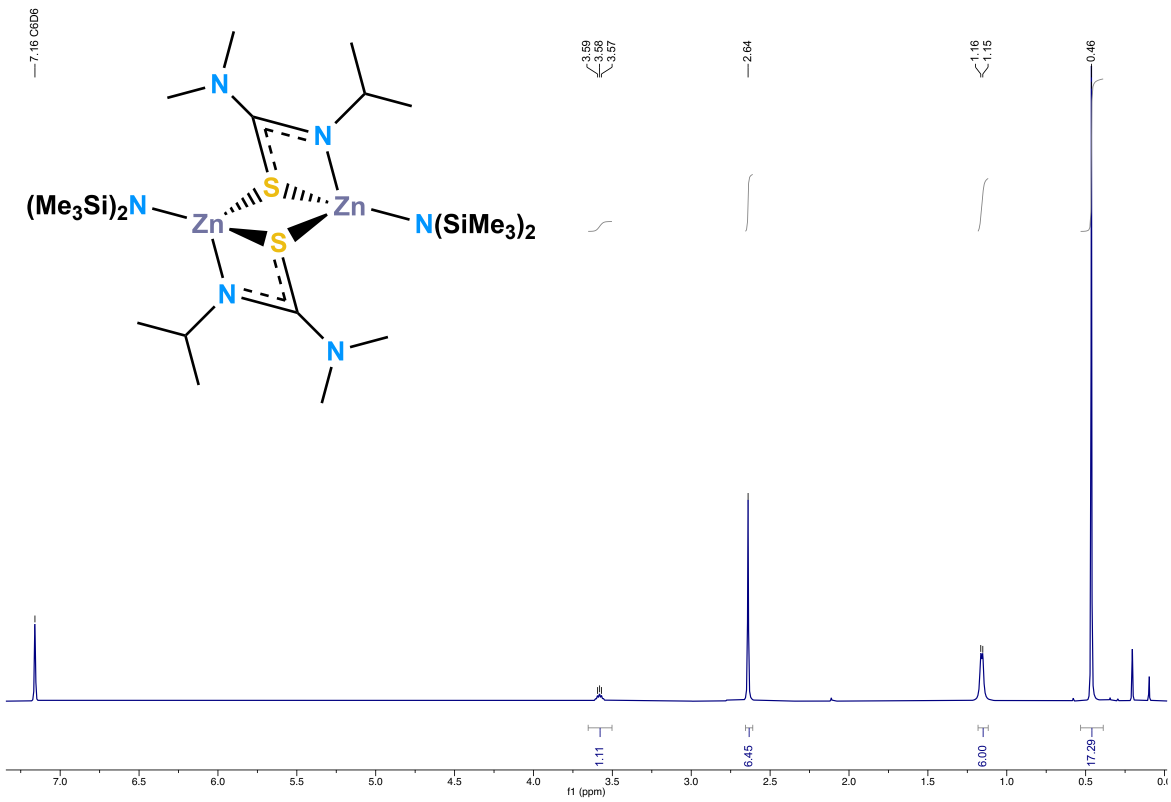

Figure S9: ^{1}H NMR spectrum of **3** in C_6D_6.

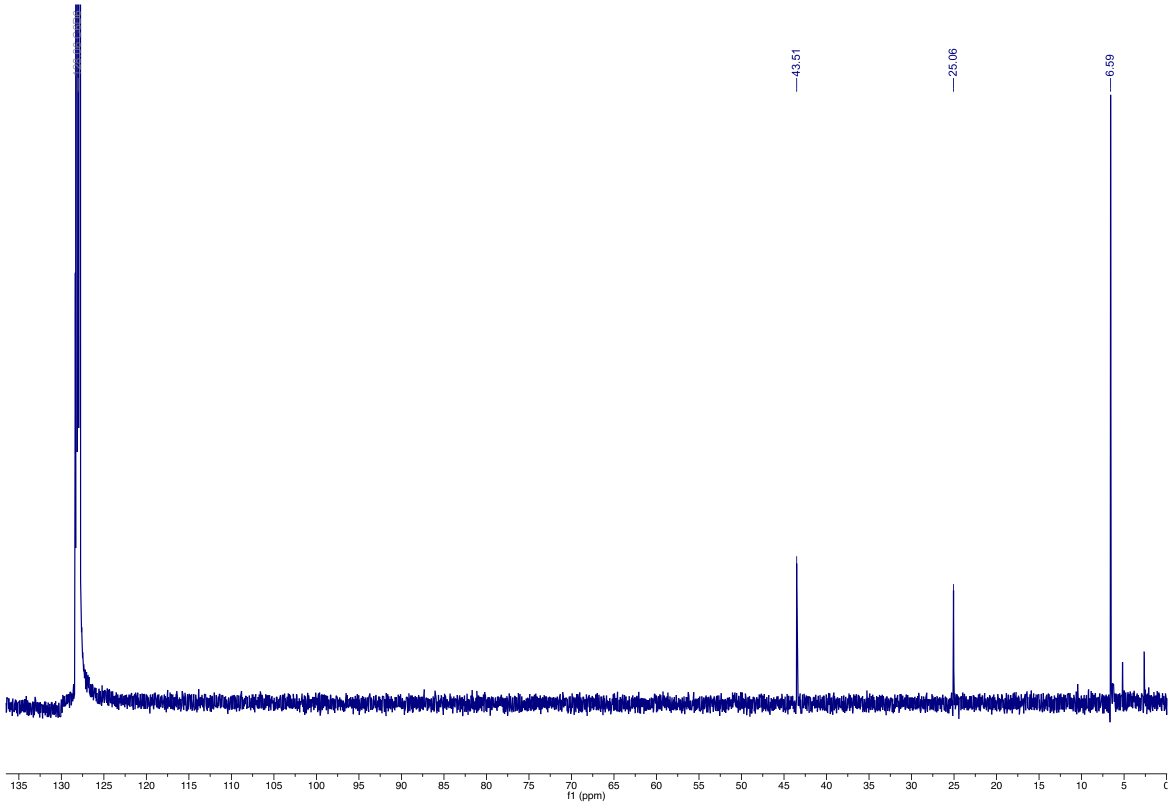

Figure S10: ^{13}C NMR spectrum of **3** in C_6D_6.

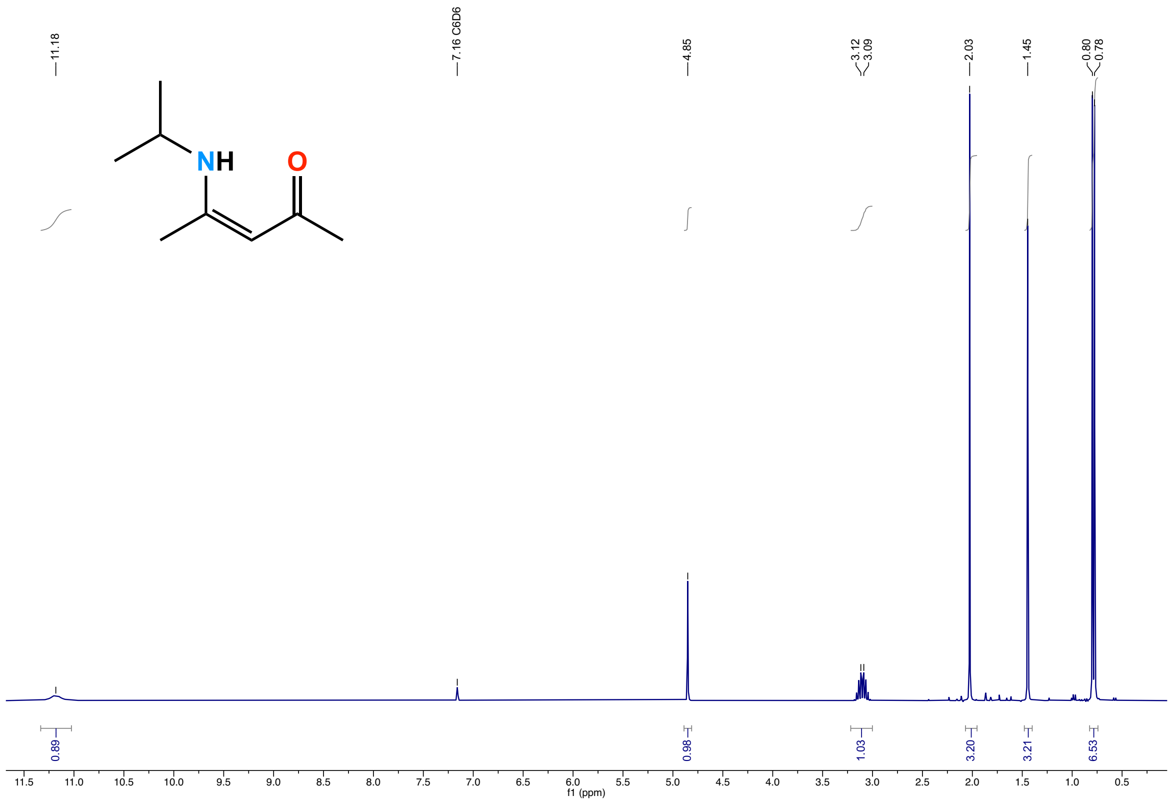

Figure S11: ^{1}H NMR spectrum of HL^{2} in C_6D_6.

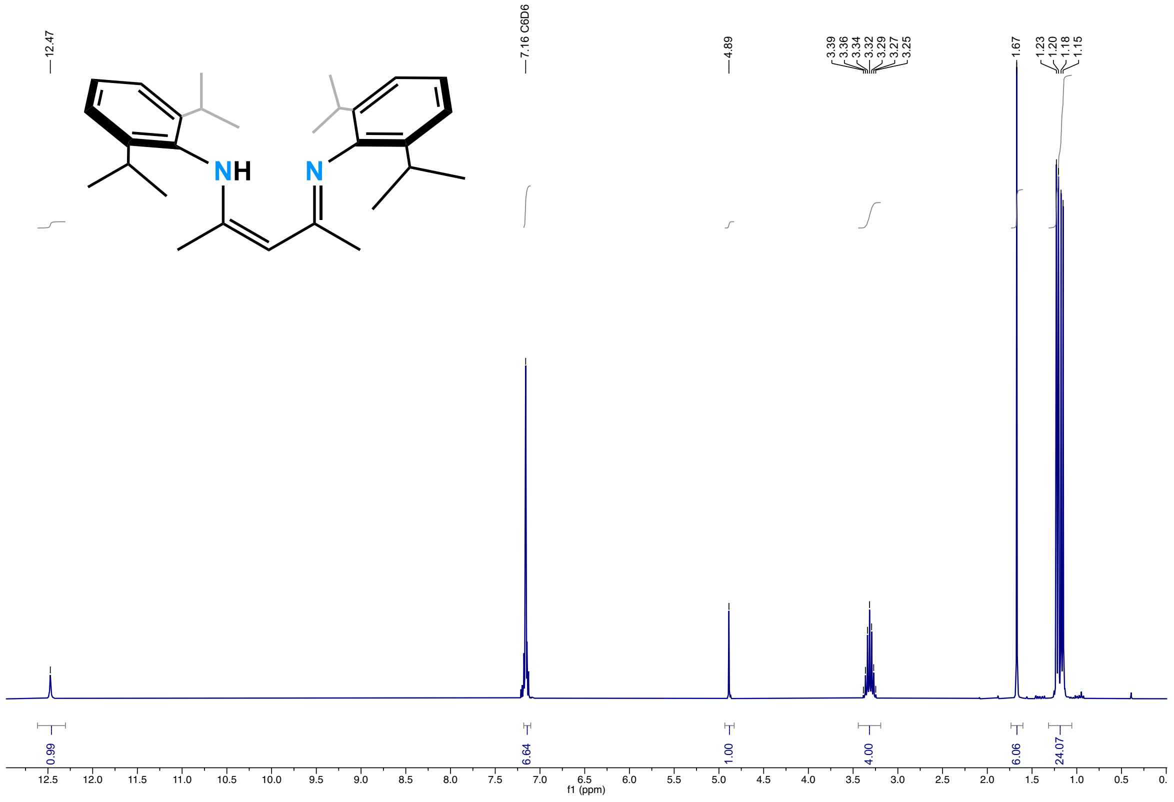

Figure S12: ^{1}H NMR spectrum of HL^{3} in C_6D_6.

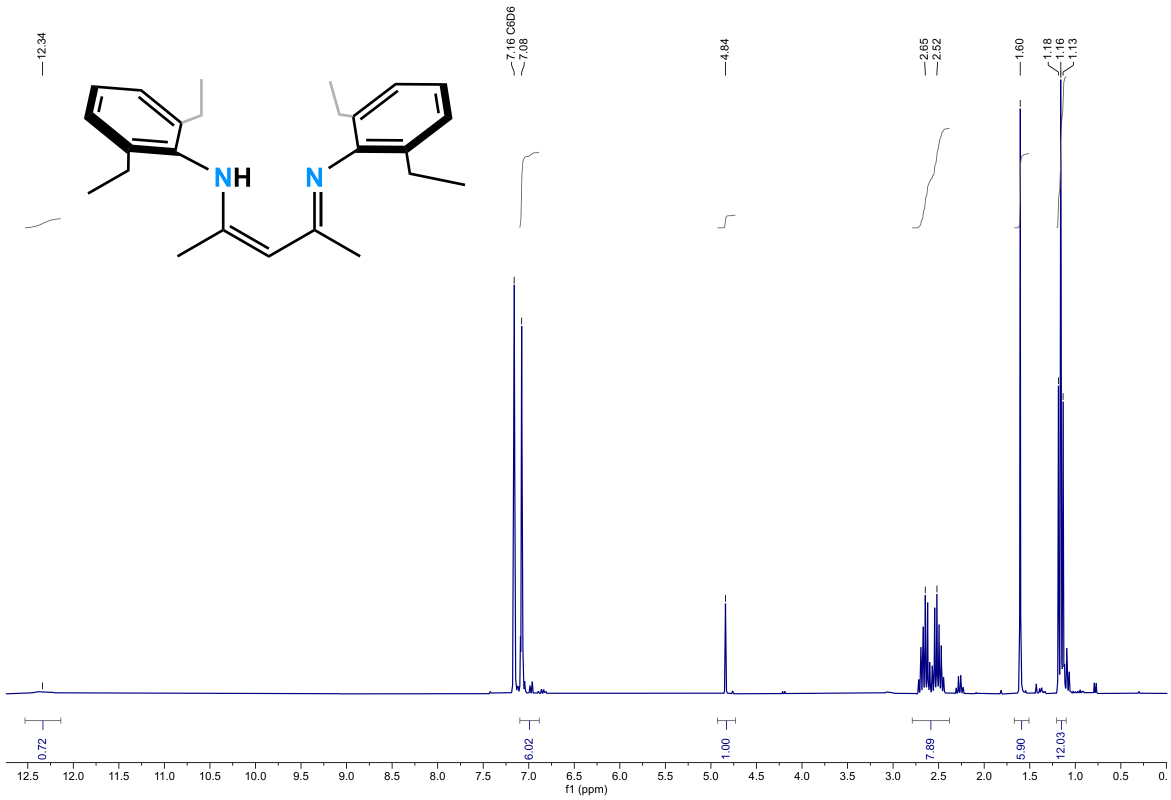

Figure S13: ^{1}H NMR spectrum of HL^{3*} in C_6D_6.

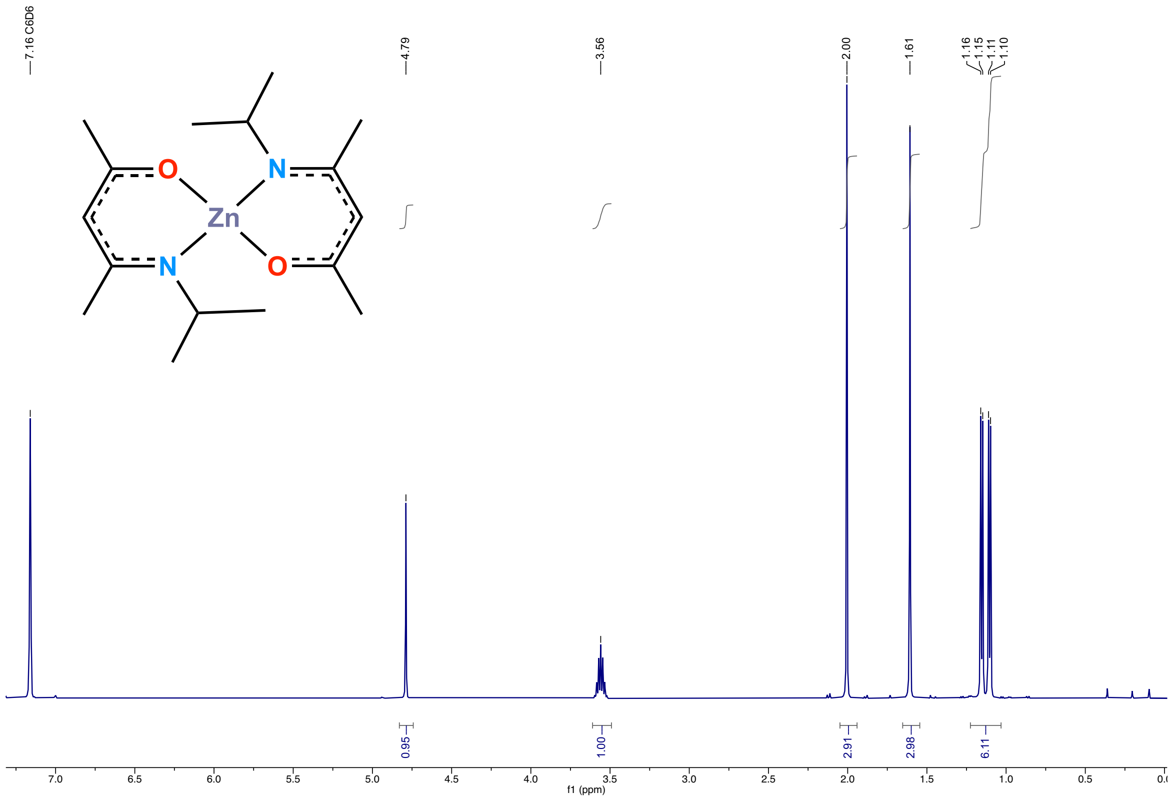

Figure S14: ^{1}H NMR spectrum of **4** in C_6D_6.

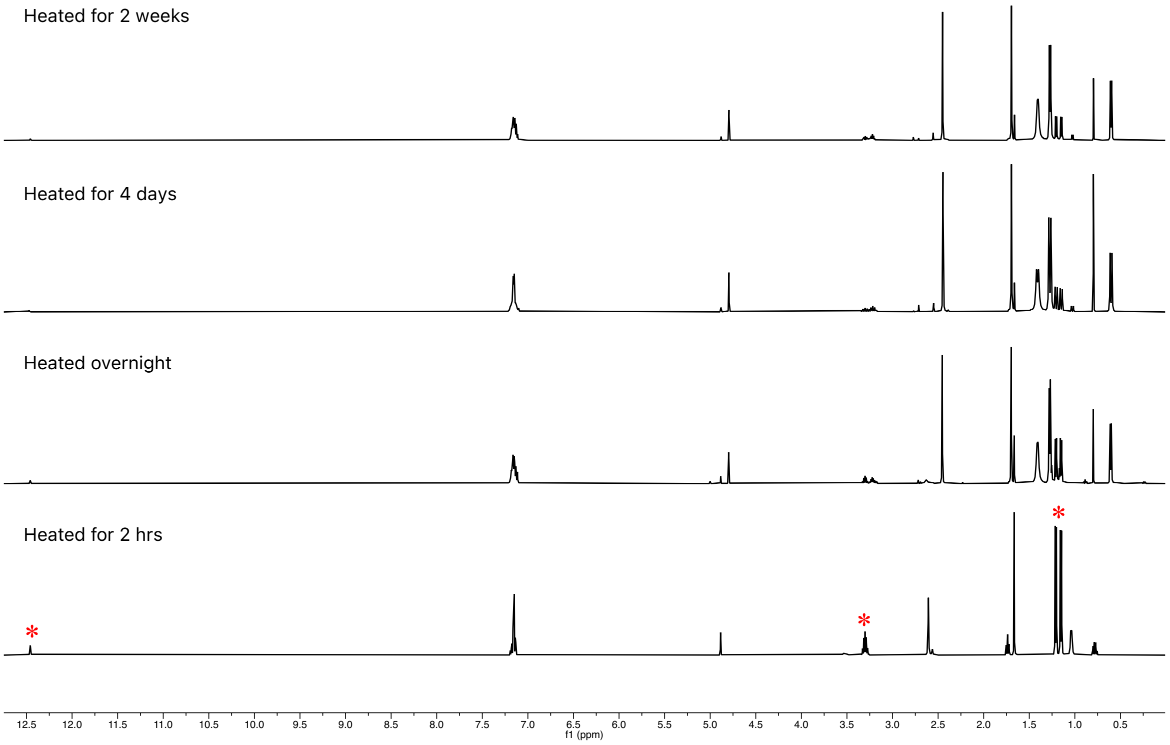

Figure S15: ^{1}H NMR spectra of the reaction of excess **1** with HL^3 in C_6D_6. Signals corresponding to BDI-Dipp (red asterisks) are still seen after 2 weeks of heating the reaction mixture.

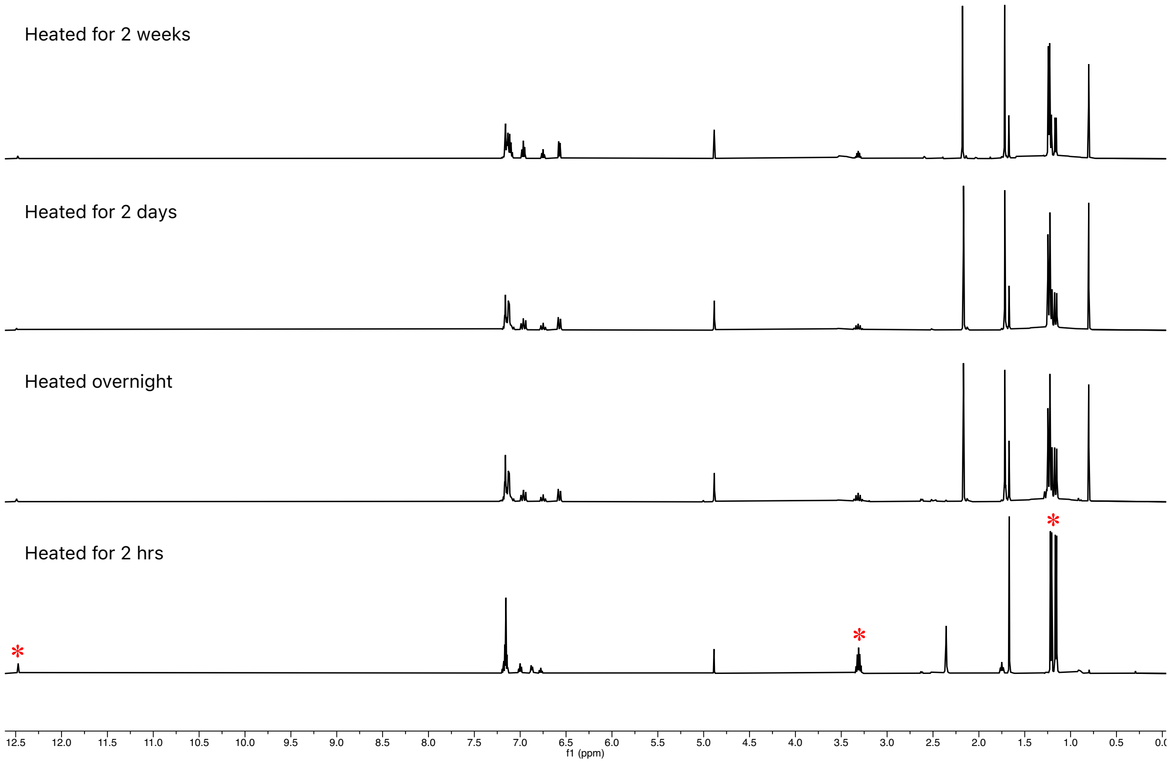

Figure S16: ^{1}H NMR spectra of the reaction of excess **2** with HL^3 in C_6D_6. Signals corresponding to BDI-Dipp (red asterisks) are still seen after 2 weeks of heating the reaction mixture.

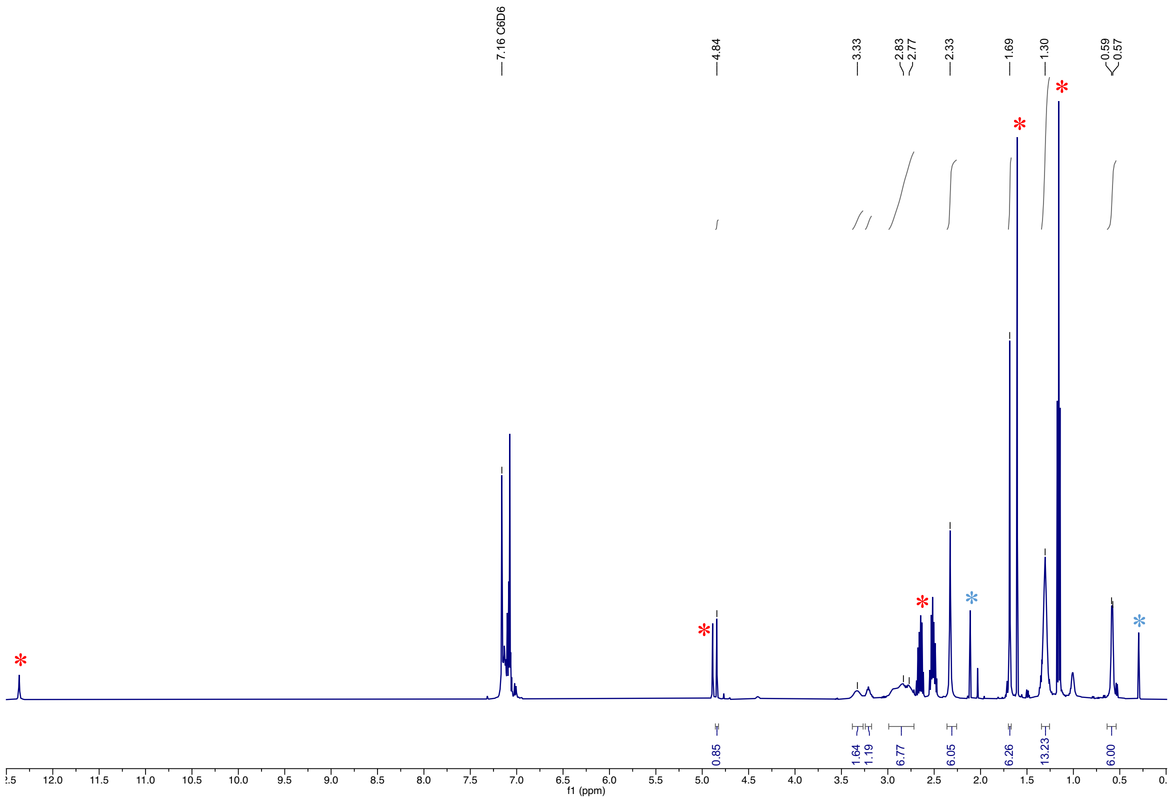

Figure S17: ^{1}H NMR spectrum of the reaction of excess **1** with HL^{3*} in C_6D_6 after heating for 2 weeks. Signals corresponding to BDI-Dep (red asterisks) are still seen after 2 weeks of heating the reaction mixture. Blue asterisks indicate toluene and grease.

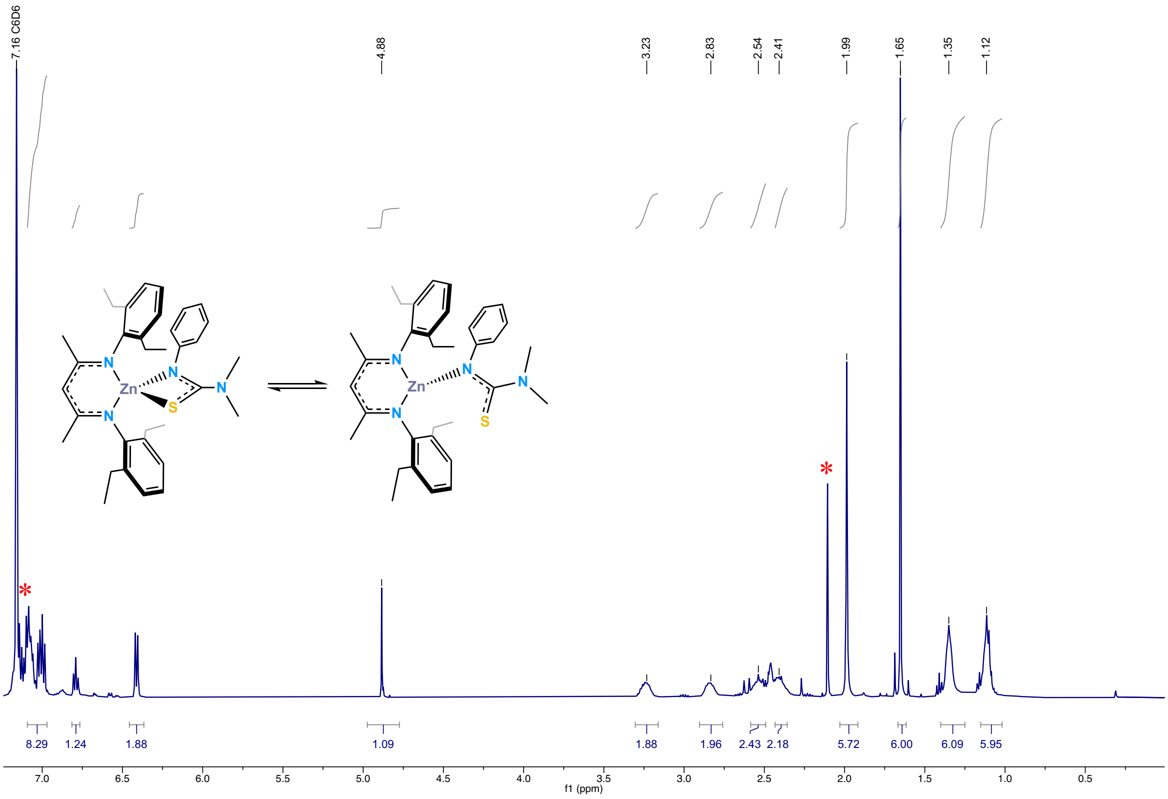

Figure S18: ^{1}H NMR of **5** in C_6D_6 at 278 K. Red asterisks denote residual toluene.

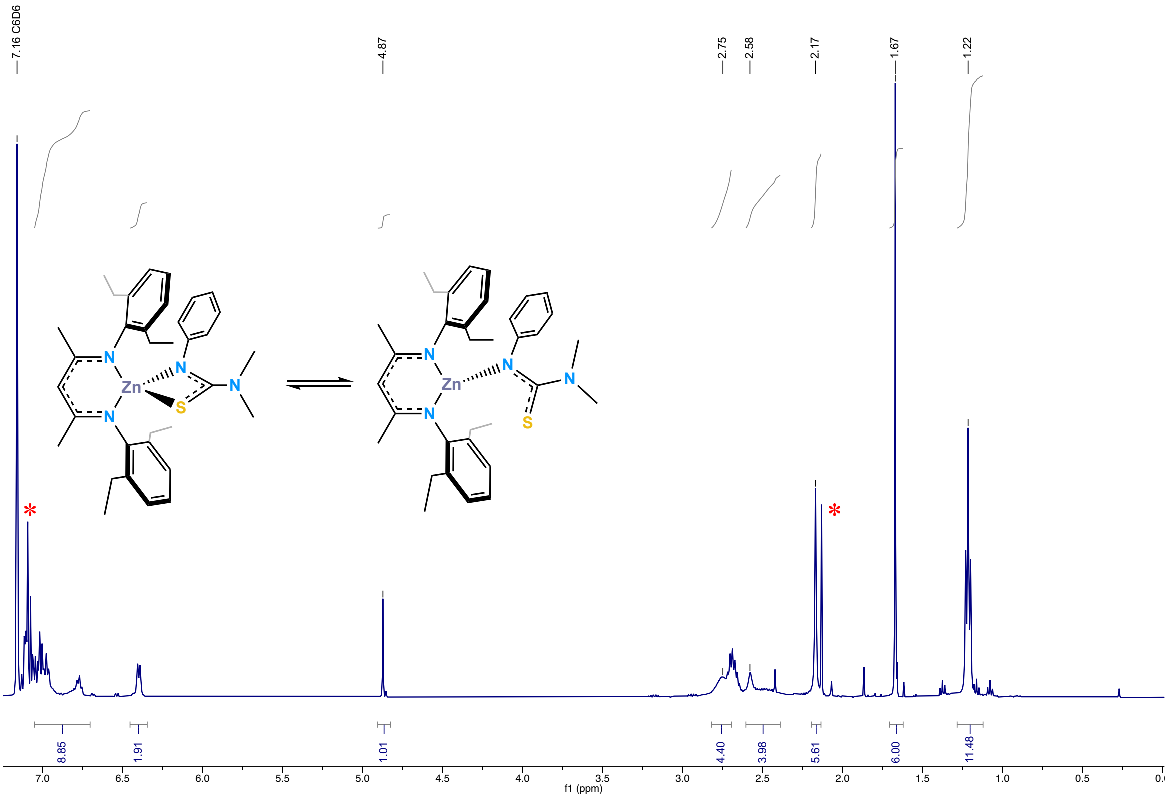

Figure S19: ^{1}H NMR of **5** in C_6D_6 at 348 K. Red asterisks denote residual toluene.

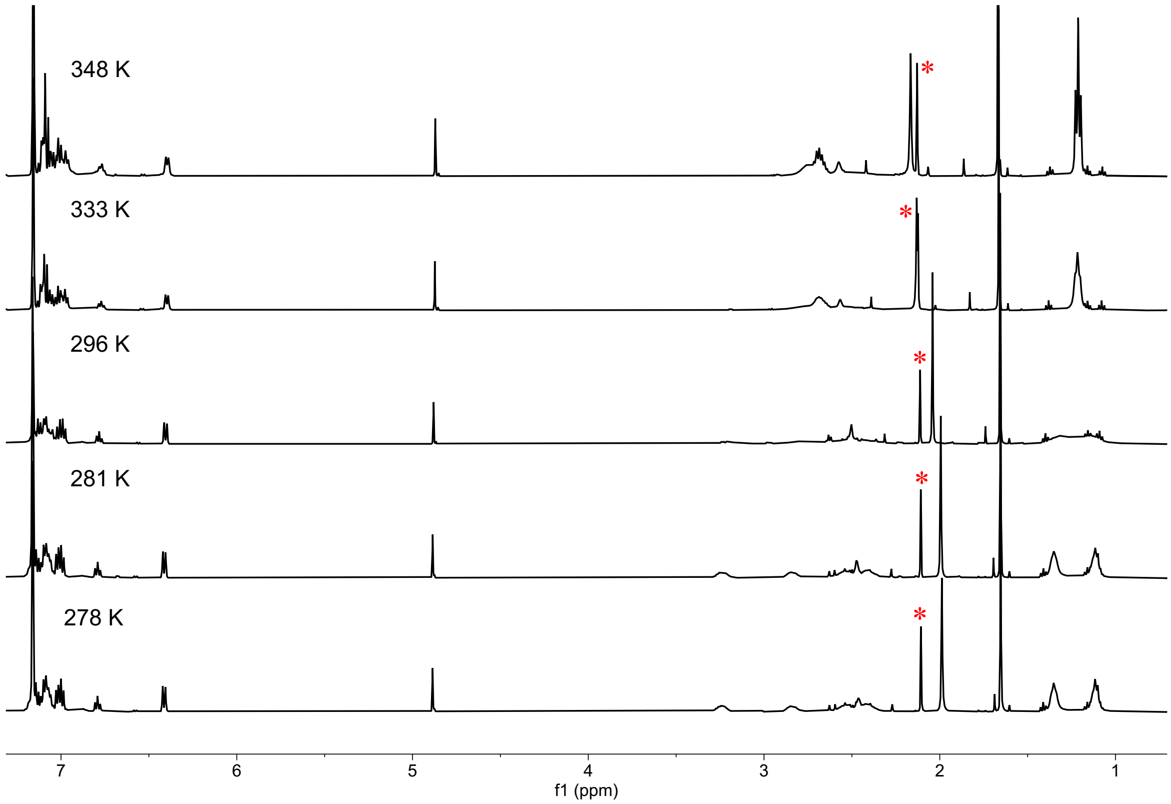

Figure S20: Stacked variable temperature ^{1}H NMR spectra of **5** in C_6D_6. The red asterisks indicate residual toluene.

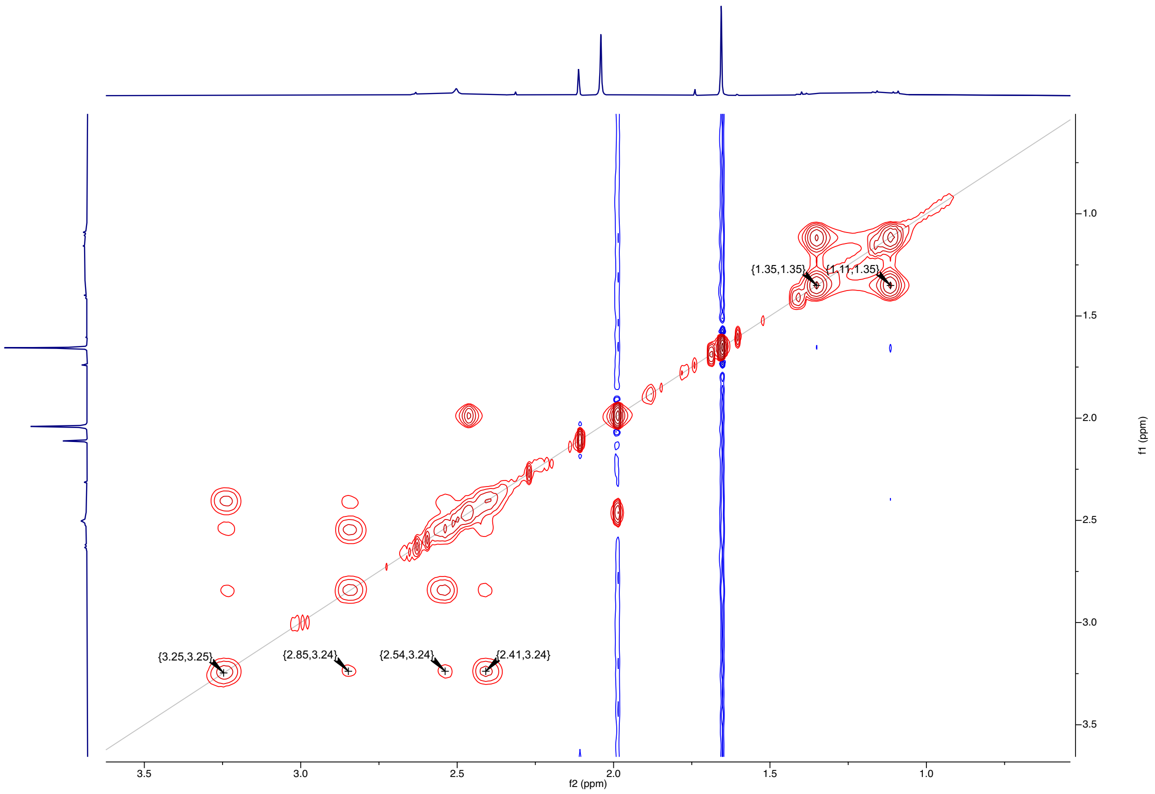

Figure S21: NOESY spectrum of **5** in C_6D_6 at 278 K, showing the region between 0 and 3.5 ppm.

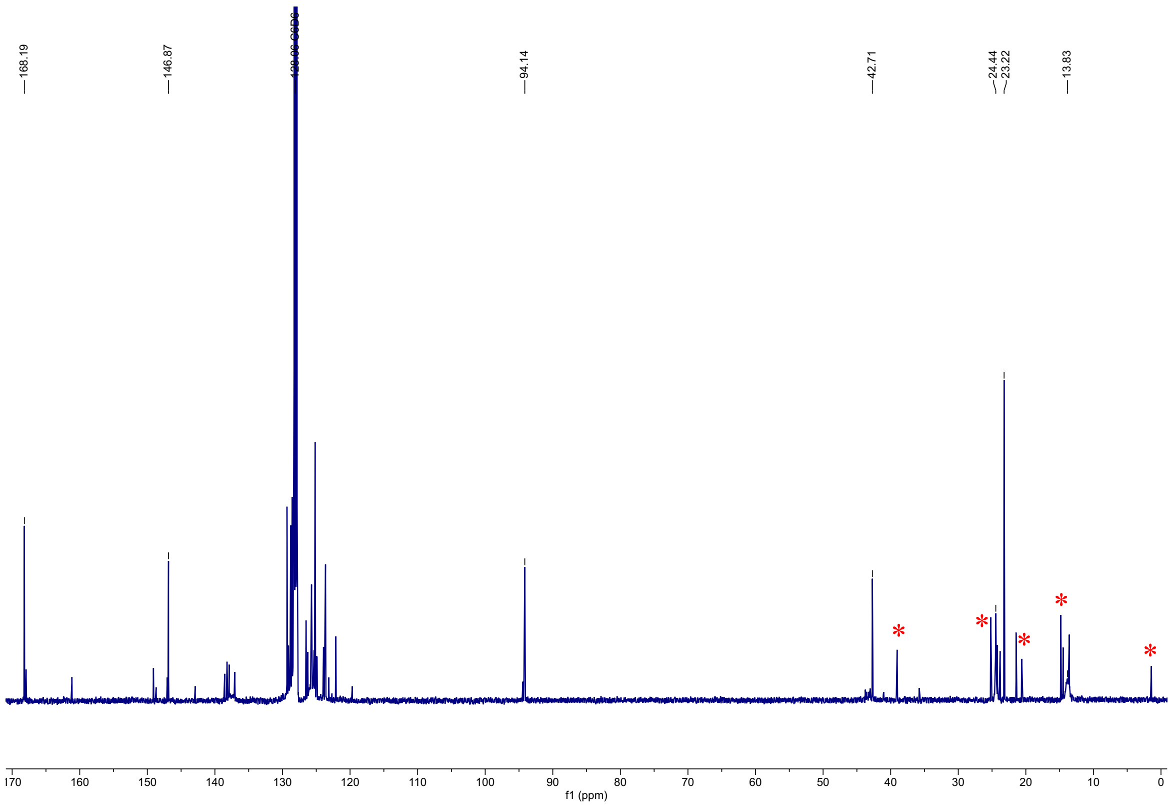

Figure S22: ^{13}C NMR of **5** in C_6D_6 at room temperature. Red asterisks represent Si grease, toluene, excess **2** and impurities.

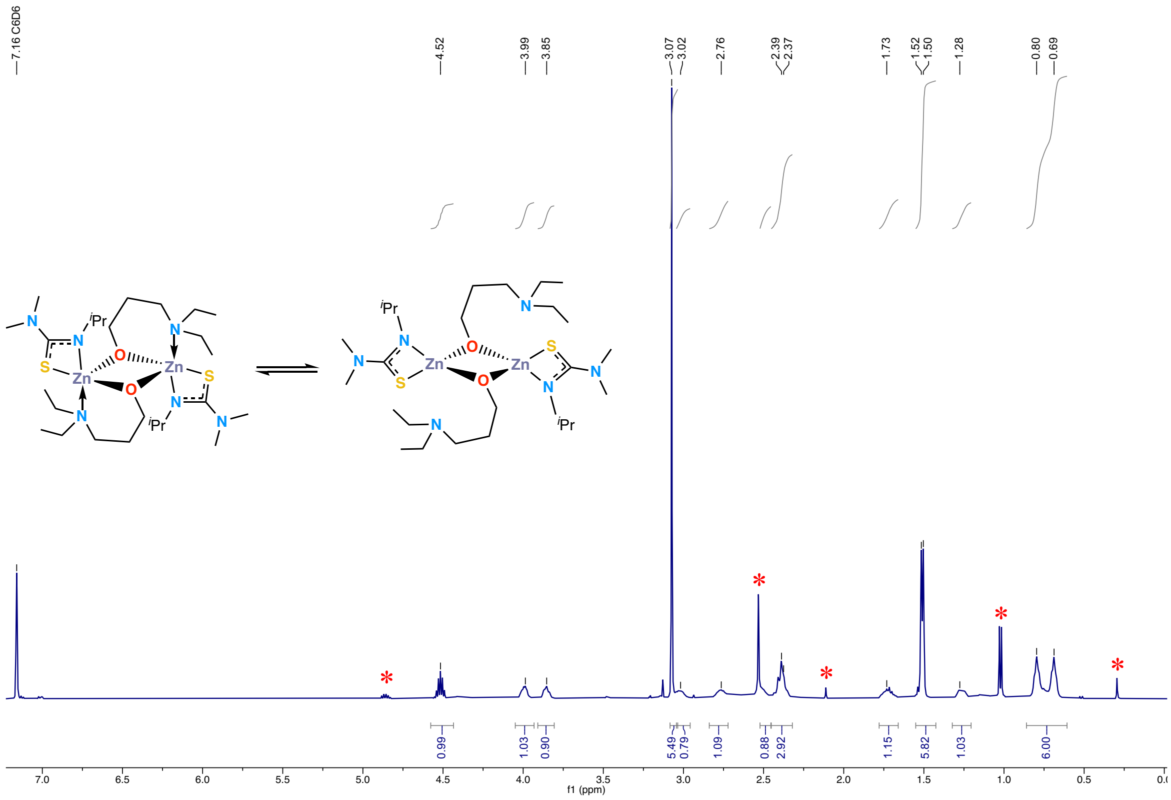

Figure S23: ^{1}H NMR spectrum of **6** in C_6D_6 at room temperature. Red asterisks indicate excess **1**, toluene and Si grease.

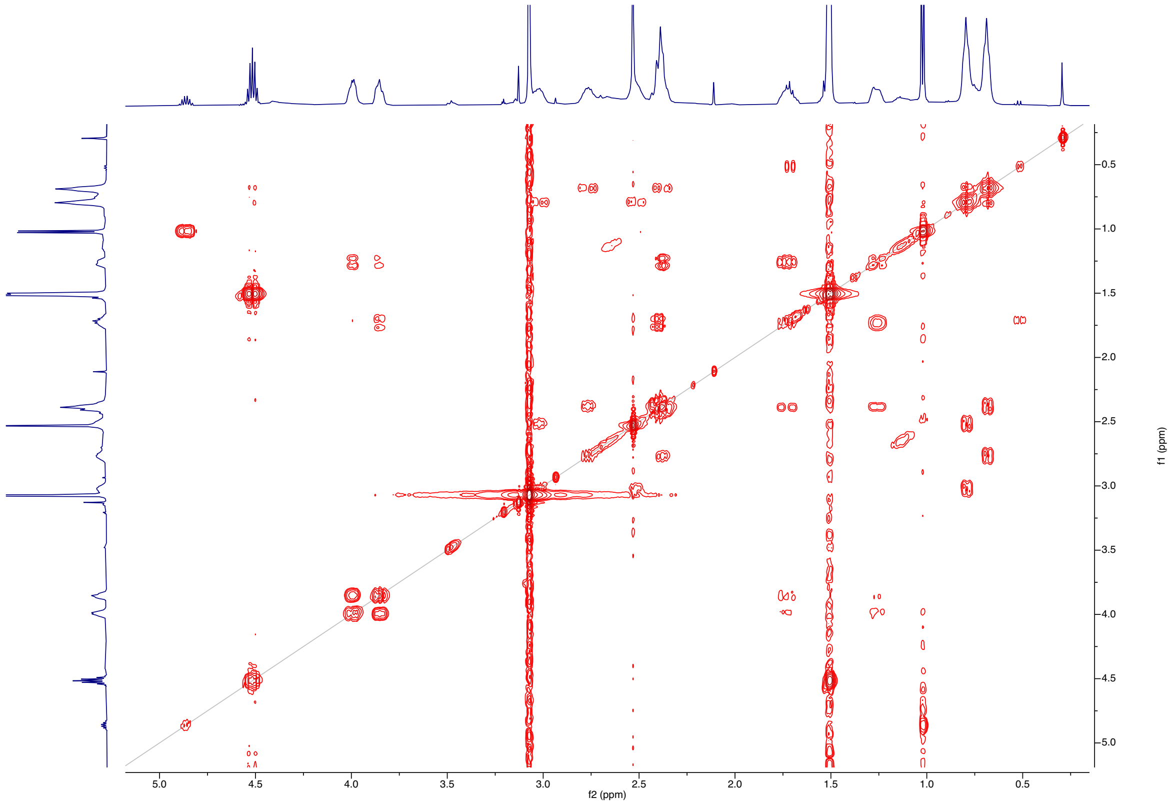

Figure S24: Room temperature COSY spectrum of **6** in C_6D_6.

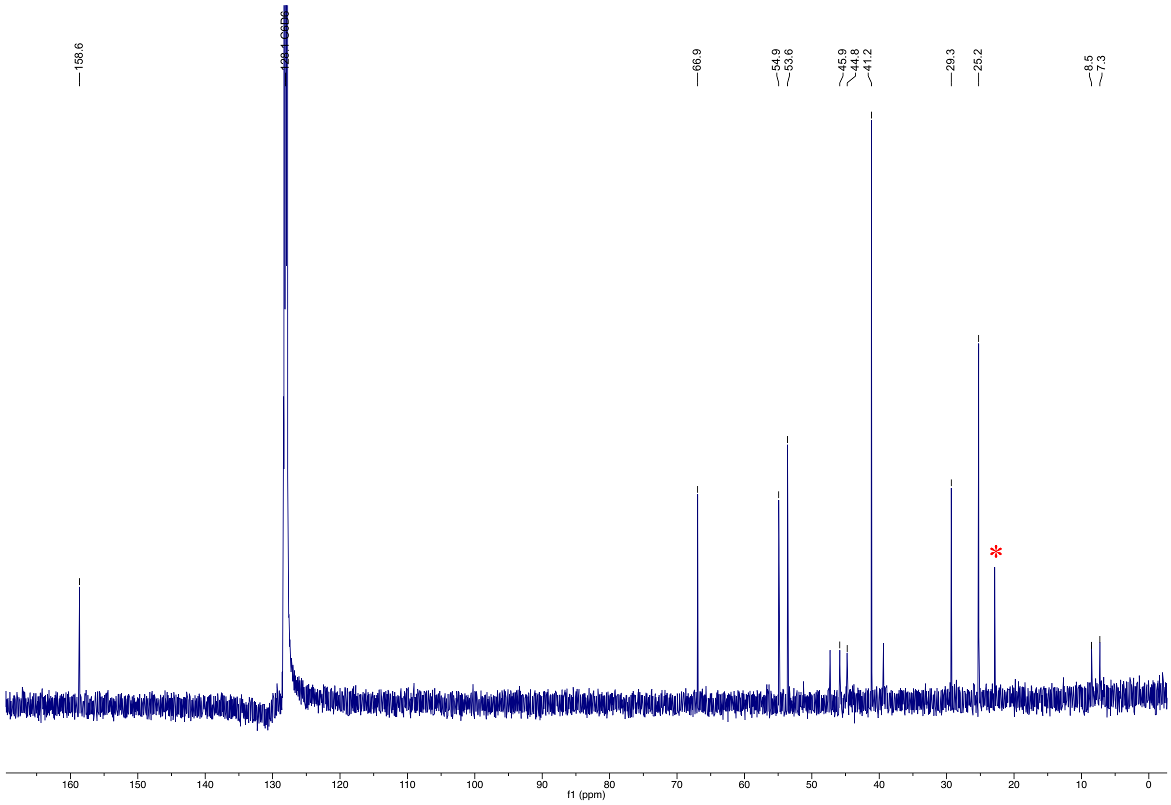

Figure S25: ^{13}C NMR spectrum of **6** in C_6D_6. The red asterisk denotes excess **1**.

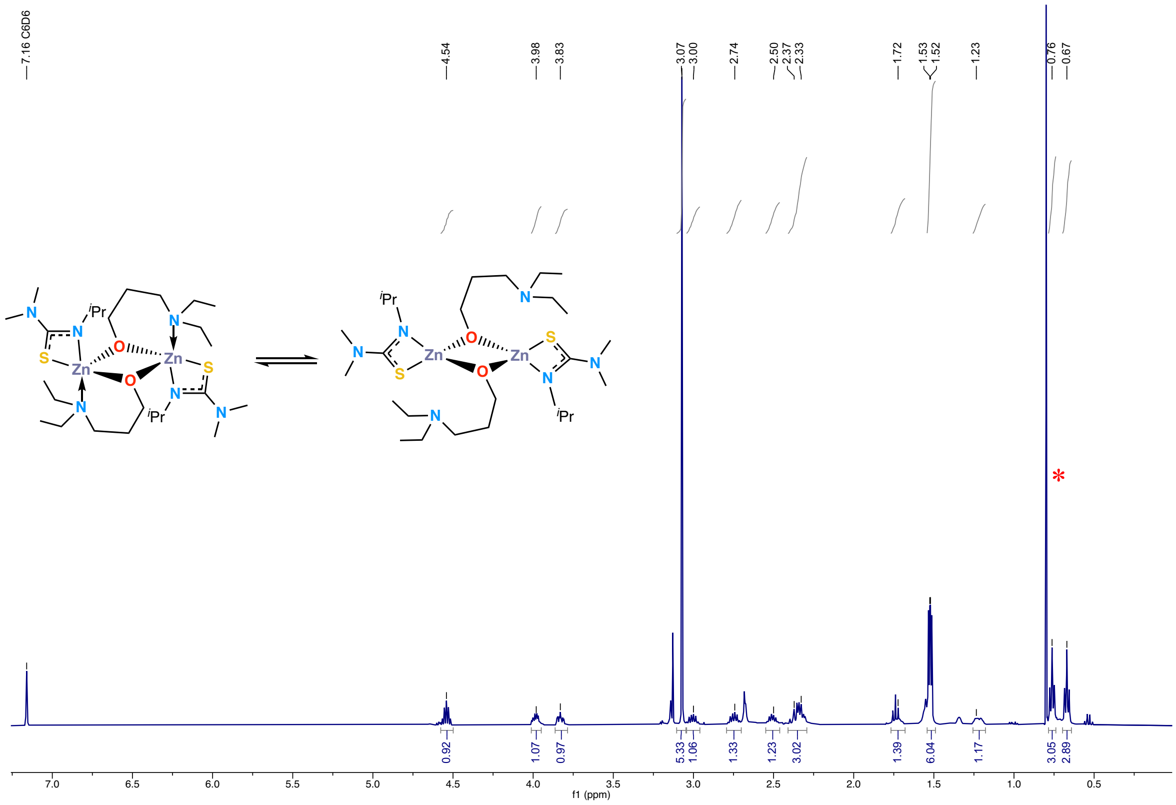

Figure S26: ^{1}H NMR spectrum of the analytical scale reaction of **1** with HL^4 in C_6D_6 at 277 K. The red asterisk indicates ethane.

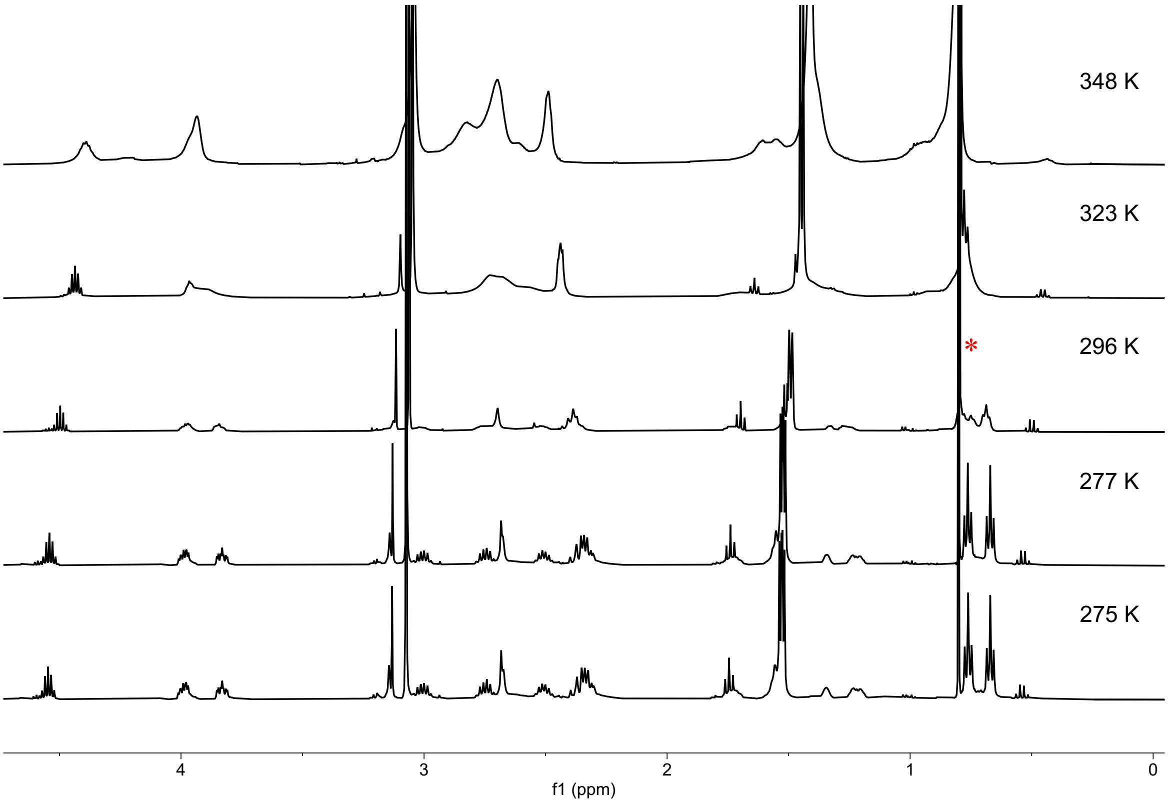

Figure S27: Stacked variable temperature ^{1}H NMR spectra of the analytical scale reaction of **1** with HL^4 in C_6D_6. The red asterisk indicates ethane.

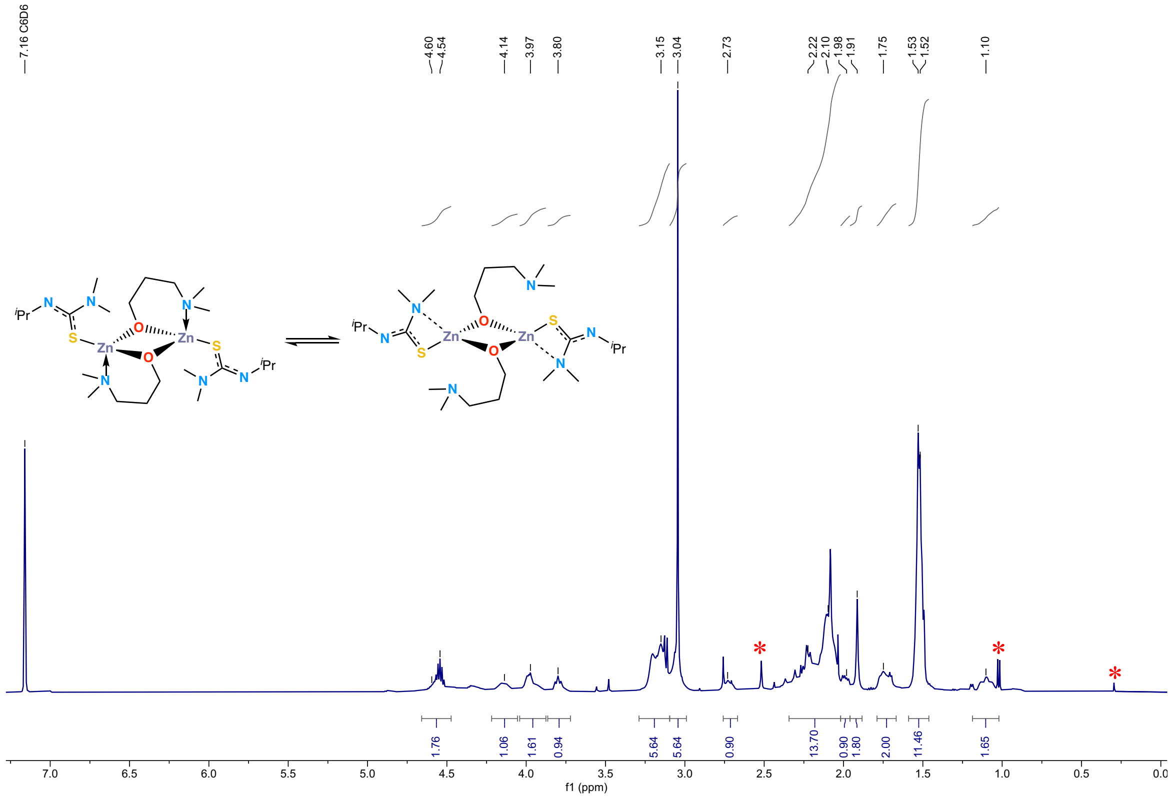

Figure S28: ^{1}H NMR spectrum of **7** in C_6D_6 at 283 K. Red asterisks indicate excess **1**, toluene and Si grease.

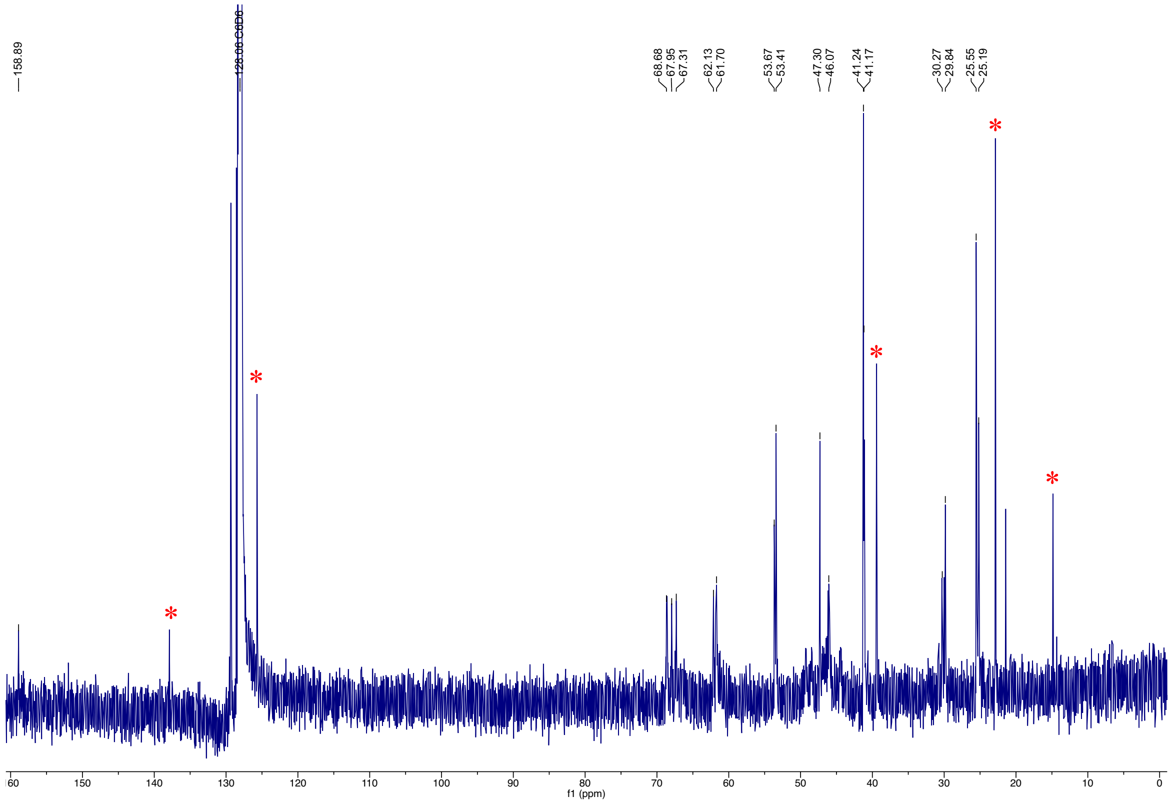

Figure S29: ^{13}C NMR spectrum of **7** in C_6D_6 at room temperature. Red asterisks indicate excess **1** and toluene.

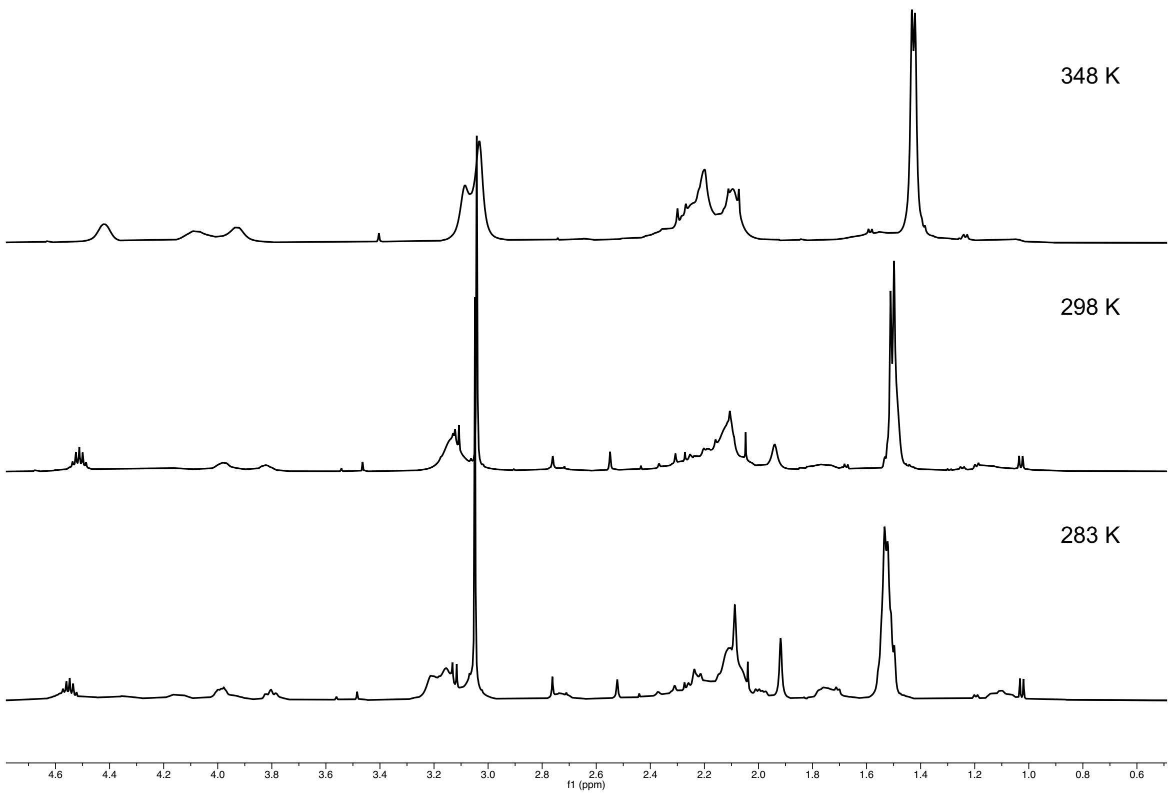

Figure S30: Stacked variable temperature ^{1}H NMR spectra of **7** C_6D_6.

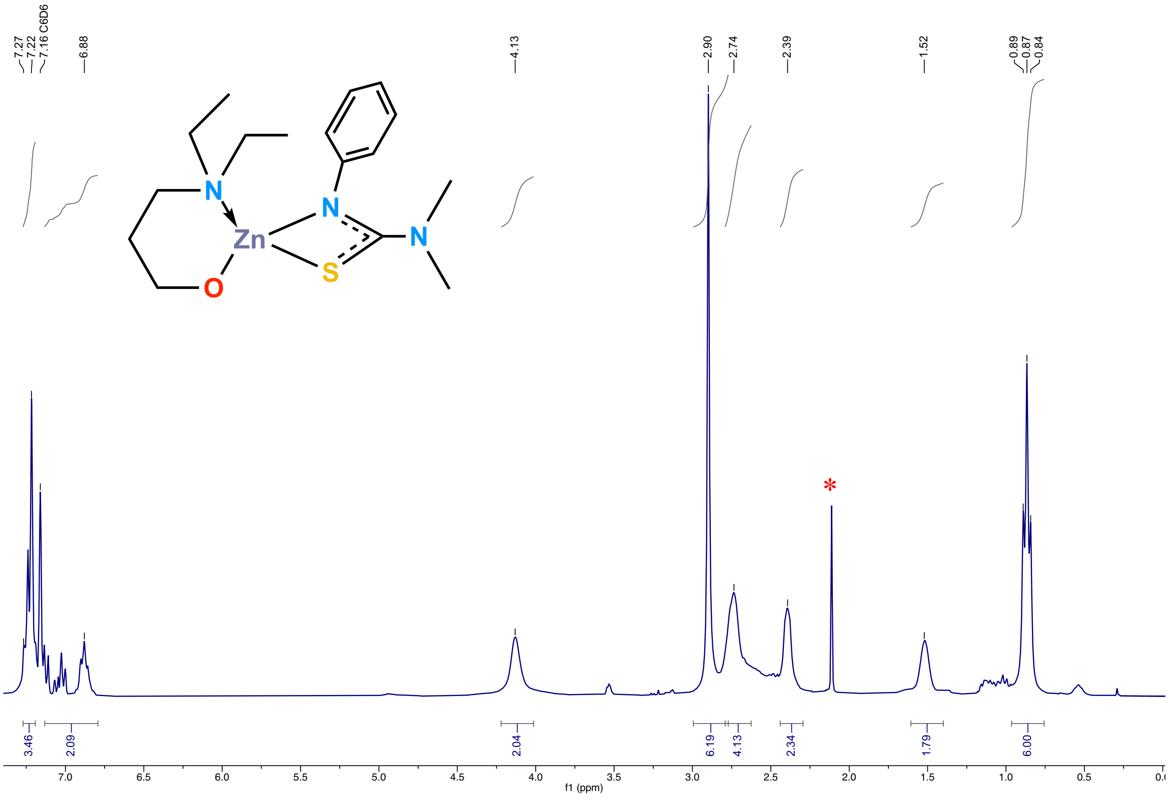

Figure S31: ^{1}H NMR spectrum of **8** in C_6D_6. The red asterisk indicates toluene.

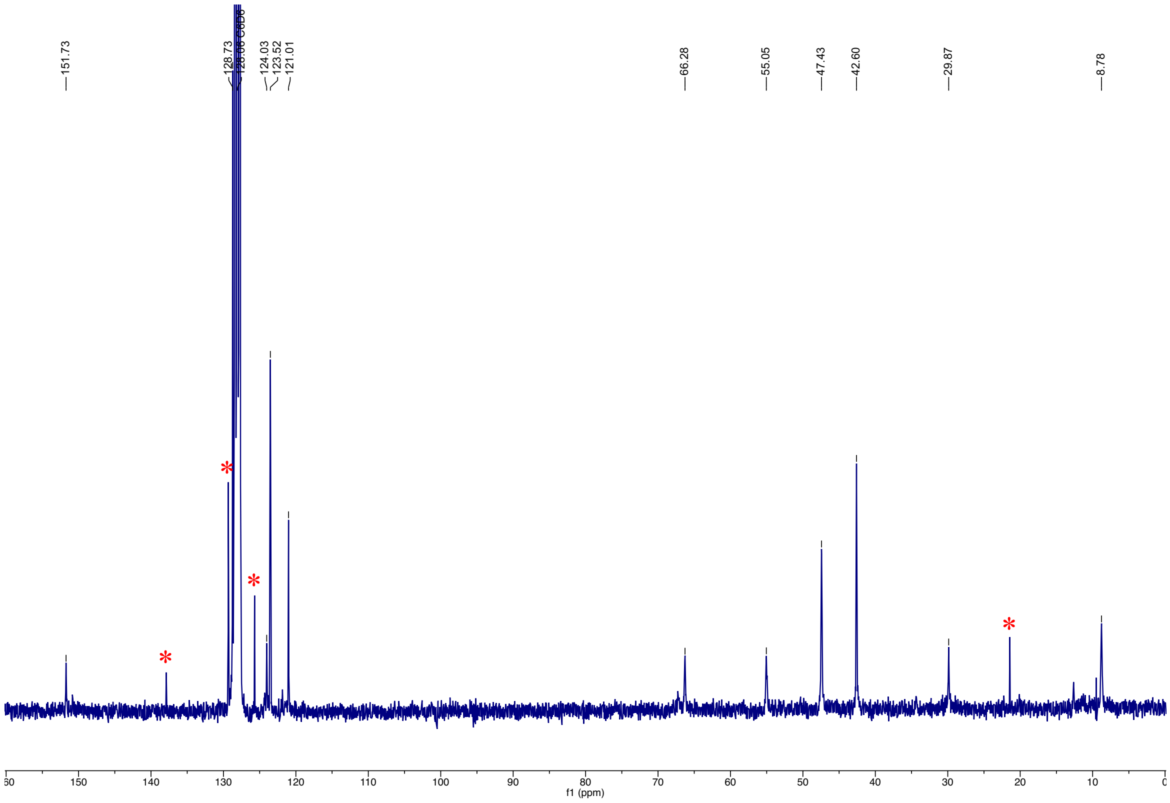

Figure S32: ^{13}C NMR spectrum of **8** in C_6D_6. Red asterisks indicate toluene.

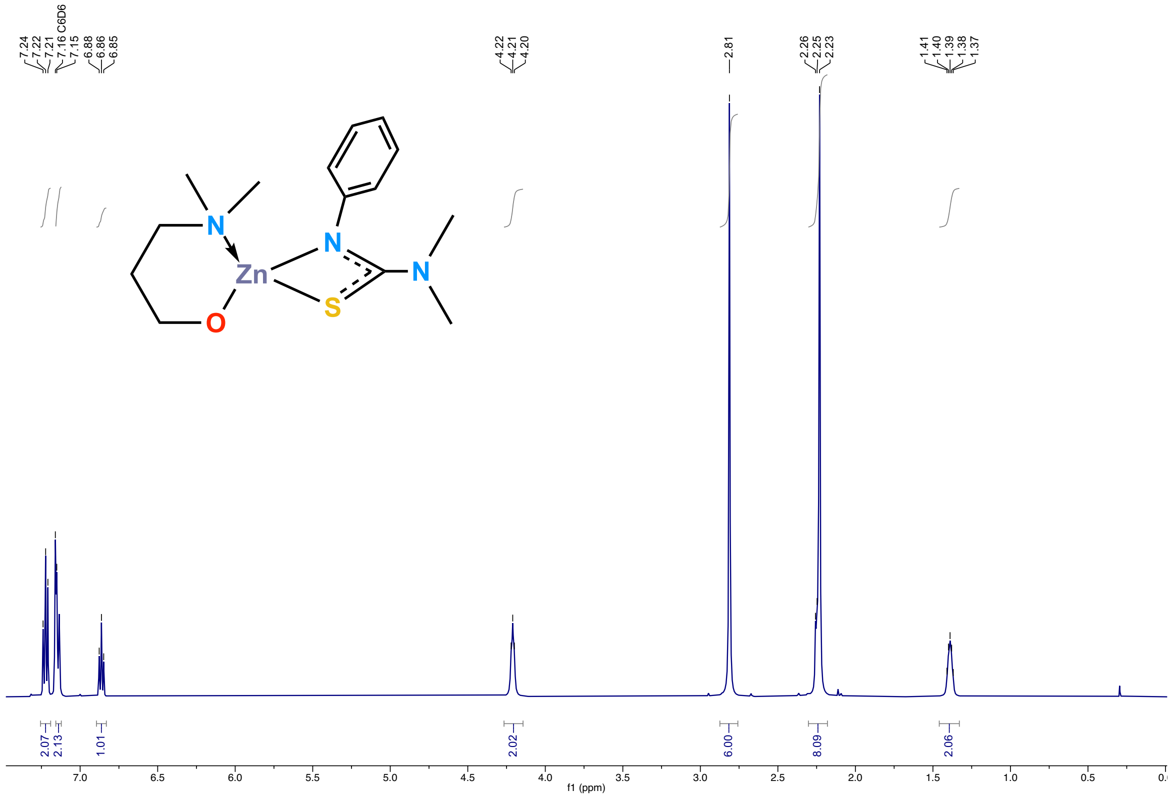

Figure S33: ^{1}H NMR spectrum of **9** in C_6D_6.

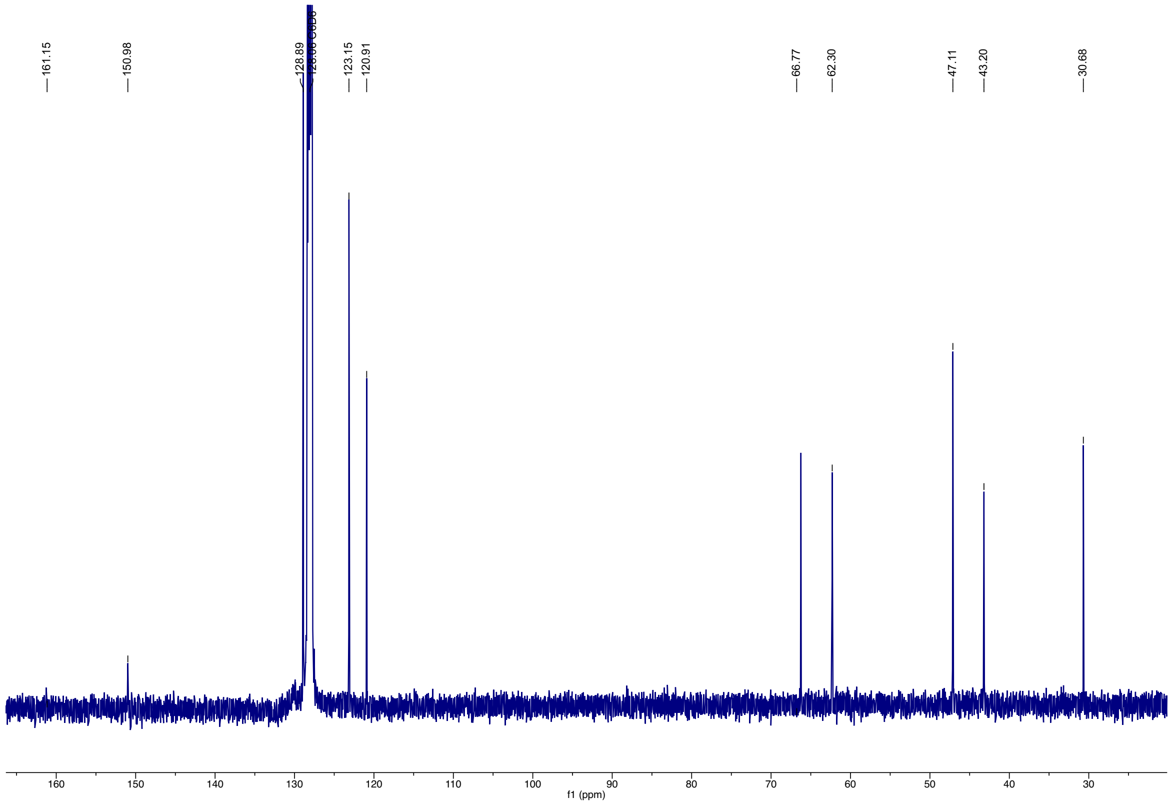

Figure S34: ^{13}C NMR spectrum of **9** in C_6D_6.

C. Crystallographic data

Crystallographic information for ***HL**^{1*}*

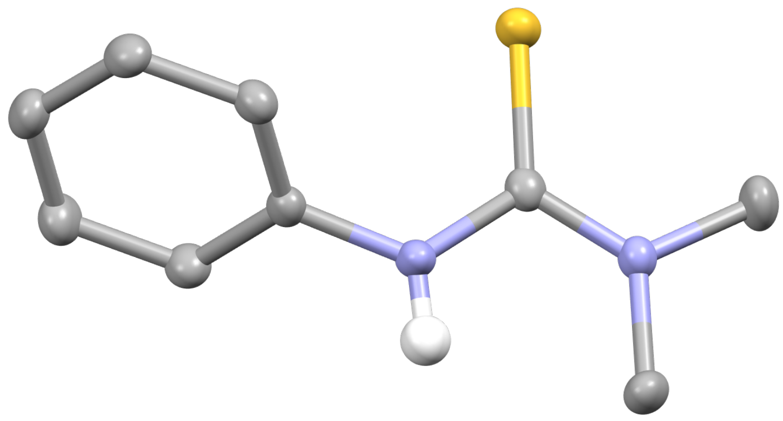

Figure S34: Solid state X-ray structure of **HL**^{1*} with thermal ellipsoids drawn at 50% probability and selected hydrogen atoms omitted for clarity.

Table S1: Crystal data and structure refinement for **HL**^{1*}

Empirical formula	$C_9H_{12}N_2S$
Formula weight	180.27
Temperature/K	150.00(10)
Crystal system	monoclinic
Space group	$P2_1/c$
a/Å	5.63230(10)
b/Å	13.4731(2)
c/Å	12.4161(3)
α/°	90
β/°	100.114(2)
γ/°	90
Volume/Å^3	927.55(3)
Z	4
ρ_{calc}g/cm^3	1.291
μ/mm^{-1}	2.645
F(000)	384.0
Crystal size/mm^3	0.2 × 0.1 × 0.05
Radiation	Cu Kα (λ = 1.54184)
2Θ range for data collection/°	9.77 to 145.388
Index ranges	$-6 \le h \le 6$, $-16 \le k \le 16$, $-14 \le l \le 15$
Reflections collected	12975
Independent reflections	1823 [R_{int} = 0.0315, R_{sigma} = 0.0143]
Data/restraints/parameters	1823/0/111
Goodness-of-fit on F^2	1.066
Final R indexes [I>=2σ (I)]	R_1 = 0.0329, wR_2 = 0.0906
Final R indexes [all data]	R_1 = 0.0341, wR_2 = 0.0918
Largest diff. peak/hole / e Å^{-3}	0.43/-0.39

Table S2: Bond lengths for **HL**[1*]

Atom	Atom	Length/Å	Atom	Atom	Length/Å
S1	C1	1.6852(15)	C4	C5	1.391(2)
N1	C1	1.3374(19)	C4	C9	1.391(2)
N1	C3	1.4617(19)	C5	C6	1.386(2)
N1	C2	1.4588(19)	C9	C8	1.387(2)
N2	C4	1.4219(18)	C6	C7	1.391(2)
N2	C1	1.3715(18)	C7	C8	1.384(2)

Table S3: Bond angles for **HL**[1*]

Atom	Atom	Atom	Angle/°	Atom	Atom	Atom	Angle/°
C1	N1	C3	122.59(12)	N1	C1	N2	115.29(13)
C1	N1	C2	121.69(12)	N2	C1	S1	121.92(11)
C2	N1	C3	115.71(12)	C6	C5	C4	119.58(13)
C1	N2	C4	124.93(12)	C8	C9	C4	119.99(14)
C5	C4	N2	121.55(13)	C5	C6	C7	120.76(14)
C5	C4	C9	119.87(13)	C8	C7	C6	119.31(14)
C9	C4	N2	118.51(13)	C7	C8	C9	120.47(14)
N1	C1	S1	122.79(11)				

*Crystallographic information for **1***

Table S4: Crystal data and structure refinement for **1**.

Empirical formula	$C_{92}H_{169.6}N_{16}S_8Zn_8$
Formula weight	2279.47
Temperature/K	150.0(4)
Crystal system	monoclinic
Space group	I2/a
a/Å	27.1708(3)
b/Å	8.98003(13)
c/Å	58.2262(7)
α/°	90
β/°	91.0722(12)
γ/°	90
Volume/Å^3	14204.4(3)
Z	5
ρ_{calc}g/cm^3	1.332
μ/mm^{-1}	3.563
F(000)	6008.0
Crystal size/mm^3	0.1 × 0.1 × 0.1
Radiation	Cu Kα (λ = 1.54184)
2Θ range for data collection/°	7.13 to 145.648
Index ranges	-33 ≤ h ≤ 33, -11 ≤ k ≤ 10, -64 ≤ l ≤ 72
Reflections collected	75291
Independent reflections	13660 [R_{int} = 0.0305, R_{sigma} = 0.0166]
Data/restraints/parameters	13660/0/710
Goodness-of-fit on F^2	1.079
Final R indexes [I>=2σ (I)]	R_1 = 0.0458, wR_2 = 0.1158
Final R indexes [all data]	R_1 = 0.0497, wR_2 = 0.1185
Largest diff. peak/hole / e Å^{-3}	1.49/-0.74

Table S4: Bond lengths for **1**.

Atom	Atom	Length/Å	Atom	Atom	Length/Å
Zn(01)	S(006)	2.5386(8)	N(00I)	C(010)	1.461(4)
Zn(01)	S(009)	2.4644(8)	N(00I)	C(013)	1.456(4)
Zn(01)	N(00B)	2.037(2)	N(00J)	C(00N)	1.352(4)
Zn(01)	C(00X)	1.977(3)	N(00J)	C(01I)	1.466(4)
Zn(02)	S(006)	2.4526(8)	N(00J)	C(01O)	1.453(4)
Zn(02)	S(009)	2.5562(8)	N(1)	C(00Q)	1.357(4)
Zn(02)	N(00E)	2.017(2)	N(1)	C(01R)	1.456(5)
Zn(02)	C(00R)	1.977(3)	N(1)	C(01X)	1.456(5)
Zn(03)	S(007)	2.5029(8)	C(00P)	C(011)	1.519(5)
Zn(03)	S(008)	2.4951(9)	C(00P)	C(018)	1.517(4)
Zn(03)	N(00D)	2.031(2)	C(00R)	C(019)	1.524(5)
Zn(03)	C(00V)	1.978(3)	C(00S)	C(00U)	1.524(4)
Zn(04)	S(007)	2.4893(8)	C(00T)	C(012)	1.396(5)
Zn(04)	S(008)	2.5052(8)	C(00T)	C(01C)	1.383(5)

Zn(04)	N(00C)	2.029(2)	C(00T)	C(01V)	1.505(5)
Zn(04)	C(00Y)	1.976(3)	C(00U)	C(01K)	1.524(5)
Zn(05)	S(00A)	2.5675(10)	C(00V)	C(015)	1.529(4)
Zn(05)	S(00A)[1]	2.4382(10)	C(00X)	C(01E)	1.512(5)
Zn(05)	N(00F)	2.028(3)	C(00Y)	C(01T)	1.525(5)
Zn(05)	C(01U)	1.981(4)	C(00Z)	C(01H)	1.514(5)
S(006)	C(27)	1.779(3)	C(00Z)	C(01W)	1.519(5)
S(007)	C(00N)	1.778(3)	C(012)	C(017)	1.383(5)
S(008)	C(00O)	1.773(3)	C(017)	C(01B)	1.382(6)
S(009)	C(00M)	1.786(3)	C(01B)	C(01N)	1.379(6)
S(00A)	Zn(05)[1]	2.4382(10)	C(01C)	C(01N)	1.373(6)
S(00A)	C(00Q)	1.777(3)	C(01D)	C(01J)	1.368(6)
N(00B)	C(27)	1.303(4)	C(01D)	C(01M)	1.376(6)
N(00B)	C(00P)	1.473(4)	C(01F)	C(020)	1.493(6)
N(00C)	C(00O)	1.310(4)	C(01F)	C(021)	1.468(6)
N(00C)	C(00Z)	1.478(4)	C(01G)	C(01Q)	1.519(5)
N(00D)	C(00N)	1.306(4)	C(01G)	C(01Y)	1.522(6)
N(00D)	C(01F)	1.478(4)	C(01J)	C(01L)	1.376(6)
N(00E)	C(00M)	1.301(4)	C(01L)	C(01P)	1.375(6)
N(00E)	C(00U)	1.478(4)	C(01M)	C(01S)	1.392(5)
N(00F)	C(00Q)	1.302(4)	C(01M)	C(01Z)	1.512(6)
N(00F)	C(01G)	1.476(4)	C(01P)	C(01S)	1.392(6)
N(00G)	C(00O)	1.355(4)	C(01U)	C(022)	1.446(7)
N(00G)	C(016)	1.462(4)	C(023)	C(023)[2]	1.551(13)
N(00G)	C(01A)	1.462(4)	C(023)	C(024)	1.263(9)
N(00H)	C(27)	1.352(4)	C(023)	C(26)	1.247(14)
N(00H)	C(00W)	1.462(4)	C(024)	C(025)	1.450(10)
N(00H)	C(014)	1.459(4)	C(025)	C(025)[2]	1.396(14)
N(00I)	C(00M)	1.355(4)			

[1]1-X,3-Y,1-Z; [2]3/2-X,+Y,1-Z

Table S6: Bond angles for **1**.

**Atom**	**Atom**	**Atom**	**Angle/°**	**Atom**	**Atom**	**Atom**	**Angle/°**
S(009)	Zn(01)	S(006)	95.99(3)	C(00M)	N(00I)	C(010)	121.9(3)
N(00B)	Zn(01)	S(006)	67.75(7)	C(00M)	N(00I)	C(013)	122.1(3)
N(00B)	Zn(01)	S(009)	102.79(7)	C(013)	N(00I)	C(010)	113.9(3)
C(00X)	Zn(01)	S(006)	131.93(11)	C(00N)	N(00J)	C(01I)	122.4(3)
C(00X)	Zn(01)	S(009)	112.38(11)	C(00N)	N(00J)	C(01O)	122.4(3)
C(00X)	Zn(01)	N(00B)	134.89(13)	C(01O)	N(00J)	C(01I)	114.8(3)
S(006)	Zn(02)	S(009)	95.82(3)	N(00B)	C(27)	S(006)	113.1(2)
N(00E)	Zn(02)	S(006)	99.78(7)	N(00B)	C(27)	N(00H)	127.8(3)
N(00E)	Zn(02)	S(009)	67.99(7)	N(00H)	C(27)	S(006)	119.0(2)
C(00R)	Zn(02)	S(006)	118.38(10)	C(00Q)	N(1)	C(01R)	121.8(3)
C(00R)	Zn(02)	S(009)	123.41(10)	C(00Q)	N(1)	C(01X)	121.5(3)
C(00R)	Zn(02)	N(00E)	136.42(12)	C(01X)	N(1)	C(01R)	114.8(3)
S(008)	Zn(03)	S(007)	96.79(3)	N(00E)	C(00M)	S(009)	113.5(2)
N(00D)	Zn(03)	S(007)	68.51(7)	N(00E)	C(00M)	N(00I)	126.9(3)
N(00D)	Zn(03)	S(008)	99.96(8)	N(00I)	C(00M)	S(009)	119.4(2)

C(00V)	Zn(03)	S(007)	127.41(10)	N(00D)	C(00N)	S(007)	112.8(2)
C(00V)	Zn(03)	S(008)	115.28(10)	N(00D)	C(00N)	N(00J)	128.2(3)
C(00V)	Zn(03)	N(00D)	136.37(13)	N(00J)	C(00N)	S(007)	118.9(2)
S(007)	Zn(04)	S(008)	96.88(3)	N(00C)	C(00O)	S(008)	113.4(2)
N(00C)	Zn(04)	S(007)	99.83(7)	N(00C)	C(00O)	N(00G)	127.6(3)
N(00C)	Zn(04)	S(008)	68.72(7)	N(00G)	C(00O)	S(008)	119.0(2)
C(00Y)	Zn(04)	S(007)	114.83(11)	N(00B)	C(00P)	C(011)	107.9(3)
C(00Y)	Zn(04)	S(008)	126.53(11)	N(00B)	C(00P)	C(018)	108.9(2)
C(00Y)	Zn(04)	N(00C)	137.47(13)	C(018)	C(00P)	C(011)	111.4(3)
S(00A)[1]	Zn(05)	S(00A)	96.82(3)	N(00F)	C(00Q)	S(00A)	114.0(2)
N(00F)	Zn(05)	S(00A)[1]	102.28(8)	N(00F)	C(00Q)	N(1)	126.0(3)
N(00F)	Zn(05)	S(00A)	67.60(8)	N(1)	C(00Q)	S(00A)	119.8(3)
C(01U)	Zn(05)	S(00A)	124.44(15)	C(019)	C(00R)	Zn(02)	112.6(2)
C(01U)	Zn(05)	S(00A)[1]	117.86(15)	C(012)	C(00T)	C(01V)	121.3(4)
C(01U)	Zn(05)	N(00F)	133.84(16)	C(01C)	C(00T)	C(012)	117.2(3)
Zn(02)	S(006)	Zn(01)	84.38(2)	C(01C)	C(00T)	C(01V)	121.5(3)
C(27)	S(006)	Zn(01)	74.44(9)	N(00E)	C(00U)	C(00S)	108.3(2)
C(27)	S(006)	Zn(02)	99.33(9)	N(00E)	C(00U)	C(01K)	109.3(3)
Zn(04)	S(007)	Zn(03)	83.25(2)	C(00S)	C(00U)	C(01K)	110.7(3)
C(00N)	S(007)	Zn(03)	74.83(10)	C(015)	C(00V)	Zn(03)	114.3(2)
C(00N)	S(007)	Zn(04)	96.47(10)	C(01E)	C(00X)	Zn(01)	119.2(2)
Zn(03)	S(008)	Zn(04)	83.08(2)	C(01T)	C(00Y)	Zn(04)	114.8(3)
C(00O)	S(008)	Zn(03)	96.01(10)	N(00C)	C(00Z)	C(01H)	110.0(3)
C(00O)	S(008)	Zn(04)	74.58(9)	N(00C)	C(00Z)	C(01W)	107.3(3)
Zn(01)	S(009)	Zn(02)	83.77(2)	C(01H)	C(00Z)	C(01W)	111.2(3)
C(00M)	S(009)	Zn(01)	99.30(9)	C(017)	C(012)	C(00T)	120.8(4)
C(00M)	S(009)	Zn(02)	73.40(9)	C(01B)	C(017)	C(012)	120.6(3)
Zn(05)[1]	S(00A)	Zn(05)	83.18(3)	C(01N)	C(01B)	C(017)	119.2(4)
C(00Q)	S(00A)	Zn(05)	73.46(11)	C(01N)	C(01C)	C(00T)	122.3(3)
C(00Q)	S(00A)	Zn(05)[1]	100.34(11)	C(01J)	C(01D)	C(01M)	121.8(3)
C(27)	N(00B)	Zn(01)	104.64(19)	N(00D)	C(01F)	C(020)	106.6(3)
C(27)	N(00B)	C(00P)	125.9(2)	C(021)	C(01F)	N(00D)	109.6(3)
C(00P)	N(00B)	Zn(01)	128.65(18)	C(021)	C(01F)	C(020)	112.7(5)
C(00O)	N(00C)	Zn(04)	103.32(19)	N(00F)	C(01G)	C(01Q)	108.3(3)
C(00O)	N(00C)	C(00Z)	126.1(3)	N(00F)	C(01G)	C(01Y)	110.0(3)
C(00Z)	N(00C)	Zn(04)	127.39(19)	C(01Q)	C(01G)	C(01Y)	110.9(4)
C(00N)	N(00D)	Zn(03)	103.8(2)	C(01D)	C(01J)	C(01L)	120.0(4)
C(00N)	N(00D)	C(01F)	126.2(3)	C(01P)	C(01L)	C(01J)	119.9(4)
C(01F)	N(00D)	Zn(03)	127.3(2)	C(01D)	C(01M)	C(01S)	118.1(4)
C(00M)	N(00E)	Zn(02)	104.94(19)	C(01D)	C(01M)	C(01Z)	120.0(4)
C(00M)	N(00E)	C(00U)	125.4(2)	C(01S)	C(01M)	C(01Z)	121.9(4)
C(00U)	N(00E)	Zn(02)	128.90(18)	C(01C)	C(01N)	C(01B)	119.9(4)
C(00Q)	N(00F)	Zn(05)	104.8(2)	C(01L)	C(01P)	C(01S)	119.8(3)
C(00Q)	N(00F)	C(01G)	125.1(3)	C(01P)	C(01S)	C(01M)	120.5(4)
C(01G)	N(00F)	Zn(05)	129.5(2)	C(022)	C(01U)	Zn(05)	113.7(4)
C(00O)	N(00G)	C(016)	122.5(3)	C(024)	C(023)	C(023)[2]	114.8(5)
C(00O)	N(00G)	C(01A)	121.6(3)	C(26)	C(023)	C(023)[2]	102.9(7)
C(016)	N(00G)	C(01A)	114.8(3)	C(26)	C(023)	C(024)	142.3(9)
C(27)	N(00H)	C(00W)	123.0(2)	C(023)	C(024)	C(025)	130.4(7)

C(27)	N(00H)	C(014)	121.6(3)	C(025)[2]	C(025)	C(024)	114.7(4)
C(014)	N(00H)	C(00W)	114.3(3)				

[1]1-X,3-Y,1-Z; [2]3/2-X,+Y,1-Z

Crystallographic information for ***2***

Table S4: Crystal data and structure refinement for **2**

Empirical formula	$C_{11}H_{16}N_2SZn$
Formula weight	273.69
Temperature/K	150.00(10)
Crystal system	monoclinic
Space group	$P2_1/n$
a/Å	8.5467(2)
b/Å	9.6250(2)
c/Å	15.3938(4)
α/°	90
β/°	105.349(3)
γ/°	90
Volume/Å^3	1221.16(5)
Z	4
ρ_{calc}g/cm^3	1.489
μ/mm^{-1}	4.129
F(000)	568.0
Crystal size/mm^3	0.2 × 0.1 × 0.1
Radiation	Cu Kα (λ = 1.54184)
2Θ range for data collection/°	10.812 to 145.156
Index ranges	$-10 \leq h \leq 10$, $-11 \leq k \leq 11$, $-19 \leq l \leq 19$
Reflections collected	17213
Independent reflections	2403 [R_{int} = 0.0352, R_{sigma} = 0.0145]
Data/restraints/parameters	2403/0/139
Goodness-of-fit on F^2	1.075
Final R indexes [I>=2σ (I)]	R_1 = 0.0258, wR_2 = 0.0687
Final R indexes [all data]	R_1 = 0.0264, wR_2 = 0.0693
Largest diff. peak/hole / e Å^{-3}	0.38/-0.36

Table S5: Bond lengths for **2**

**Atom**	**Atom**	**Length/Å**	**Atom**	**Atom**	**Length/Å**
Zn1	S1[1]	2.4876(4)	N2	C5	1.463(2)
Zn1	S1	2.5223(4)	N2	C4	1.463(2)
Zn1	N1	2.0368(14)	C6	C11	1.391(2)
Zn1	C1	1.9730(18)	C6	C7	1.390(2)
S1	Zn1[1]	2.4876(4)	C11	C10	1.389(2)
S1	C3	1.7678(16)	C7	C8	1.387(3)
N1	C3	1.323(2)	C1	C2	1.526(3)
N1	C6	1.422(2)	C10	C9	1.384(3)
N2	C3	1.341(2)	C8	C9	1.383(3)

[1]2-X,1-Y,1-Z

Table S6: Bond angles for **2**

Atom	Atom	Atom	Angle/°	Atom	Atom	Atom	Angle/°
S1[1]	Zn1	S1	99.474(14)	C5	N2	C4	113.85(14)
N1	Zn1	S1	68.46(4)	N1	C3	S1	113.45(12)
N1	Zn1	S1[1]	97.53(4)	N1	C3	N2	126.56(15)
C1	Zn1	S1[1]	113.78(5)	N2	C3	S1	119.87(12)
C1	Zn1	S1	120.08(5)	C11	C6	N1	119.44(14)
C1	Zn1	N1	143.97(6)	C7	C6	N1	121.42(14)
Zn1[1]	S1	Zn1	80.523(14)	C7	C6	C11	119.03(15)
C3	S1	Zn1	74.70(5)	C10	C11	C6	120.34(15)
C3	S1	Zn1[1]	93.58(5)	C8	C7	C6	120.24(16)
C3	N1	Zn1	103.25(10)	C2	C1	Zn1	114.32(12)
C3	N1	C6	124.05(14)	C9	C10	C11	120.44(16)
C6	N1	Zn1	129.75(10)	C9	C8	C7	120.69(17)
C3	N2	C5	123.16(14)	C8	C9	C10	119.23(16)
C3	N2	C4	122.59(14)				

[1]2-X,1-Y,1-Z

Crystallographic information for ***6***

Table S7: Crystal data and structure refinement for **6**

Identification code	xstr1294
Empirical formula	$C_{16.5}H_{29}N_3OSZn$
Formula weight	382.86
Temperature/K	151(1)
Crystal system	monoclinic
Space group	$P2_1/n$
a/Å	10.72190(10)
b/Å	10.99980(10)
c/Å	17.8205(2)
α/°	90
β/°	104.1930(10)
γ/°	90
Volume/Å^3	2037.57(4)
Z	4
ρ_{calc}g/cm^3	1.248
μ/mm^{-1}	2.667
F(000)	812.0
Crystal size/mm^3	0.1 × 0.1 × 0.05
Radiation	Cu Kα (λ = 1.54184)
2Θ range for data collection/°	11.92 to 146.906
Index ranges	-13 ≤ h ≤ 13, -13 ≤ k ≤ 13, -22 ≤ l ≤ 22
Reflections collected	32620
Independent reflections	4076 [R_{int} = 0.0307, R_{sigma} = 0.0125]
Data/restraints/parameters	4076/0/198
Goodness-of-fit on F^2	1.039
Final R indexes [I>=2σ (I)]	R_1 = 0.0432, wR_2 = 0.1209
Final R indexes [all data]	R_1 = 0.0442, wR_2 = 0.1219
Largest diff. peak/hole / e Å^{-3}	1.26/-0.73

Table S8: Bond lengths for **6**

**Atom**	**Atom**	**Length/Å**	**Atom**	**Atom**	**Length/Å**
Zn1	Zn1^1	2.8959(6)	C3	C2	1.518(4)
Zn1	S1	2.2532(7)	C6	C7	1.522(4)
Zn1	O1	1.9761(16)	C4	C5	1.510(4)
Zn1	O1^1	1.9776(16)	C9	C10	1.518(5)
Zn1	N1^1	2.0882(19)	C9	C11	1.523(5)
S1	C8	1.791(3)	C16	C18	1.383(9)
O1	Zn1^1	1.9776(16)	C16	C17	1.499(8)
O1	C1	1.411(3)	C16	C17^2	1.326(8)
N1	Zn1^1	2.0882(19)	C16	C15	1.289(9)
N1	C3	1.501(3)	C14	C18	1.455(10)
N1	C6	1.495(3)	C14	C17	1.370(9)
N1	C4	1.494(3)	C14	C15^2	1.395(10)
N2	C8	1.269(4)	C18	C17	1.509(11)
N2	C9	1.468(4)	C17	C16^2	1.326(8)

N3	C8	1.402(4)	C17	C17[2]	1.526(14)
N3	C13	1.456(4)	C17	C15[2]	1.131(11)
N3	C12	1.457(4)	C15	C14[2]	1.395(10)
C1	C2	1.519(3)	C15	C17[2]	1.131(11)

[1]1-X,1-Y,1-Z; [2]-X,2-Y,1-Z

Table S9: Bond angles for **6**

**Atom**	**Atom**	**Atom**	**Angle/°**	**Atom**	**Atom**	**Atom**	**Angle/°**
S1	Zn1	Zn1[1]	132.04(2)	N2	C9	C11	108.5(3)
O1[1]	Zn1	Zn1[1]	42.89(5)	C10	C9	C11	111.8(3)
O1	Zn1	Zn1[1]	42.93(5)	C18	C16	C17	63.0(5)
O1[1]	Zn1	S1	126.49(5)	C17[2]	C16	C18	128.0(6)
O1	Zn1	S1	112.72(5)	C17[2]	C16	C17	65.1(5)
O1	Zn1	O1[1]	85.82(7)	C15	C16	C18	179.2(6)
O1[1]	Zn1	N1[1]	96.97(7)	C15	C16	C17[2]	51.2(5)
O1	Zn1	N1[1]	106.55(7)	C15	C16	C17	116.2(5)
N1[1]	Zn1	Zn1[1]	106.10(6)	C17	C14	C18	64.5(5)
N1[1]	Zn1	S1	121.70(6)	C17	C14	C15[2]	48.3(5)
C8	S1	Zn1	98.69(9)	C15[2]	C14	C18	112.7(6)
Zn1	O1	Zn1[1]	94.18(7)	C16	C18	C14	117.3(6)
C1	O1	Zn1[1]	122.91(14)	C16	C18	C17	62.3(5)
C1	O1	Zn1	126.53(15)	C14	C18	C17	55.0(4)
C3	N1	Zn1[1]	107.39(14)	C16[2]	C17	C16	114.9(5)
C6	N1	Zn1[1]	108.62(14)	C16[2]	C17	C14	129.7(6)
C6	N1	C3	111.8(2)	C16[2]	C17	C18	169.4(7)
C4	N1	Zn1[1]	109.38(15)	C16	C17	C18	54.8(4)
C4	N1	C3	107.47(19)	C16[2]	C17	C17[2]	62.9(5)
C4	N1	C6	112.03(19)	C16	C17	C17[2]	52.0(5)
C8	N2	C9	120.4(2)	C14	C17	C16	115.3(6)
C8	N3	C13	118.8(2)	C14	C17	C18	60.5(5)
C8	N3	C12	115.3(3)	C14	C17	C17[2]	167.0(9)
C13	N3	C12	112.3(3)	C18	C17	C17[2]	106.7(8)
O1	C1	C2	114.09(19)	C15[2]	C17	C16	176.5(8)
N1	C3	C2	115.7(2)	C15[2]	C17	C16[2]	62.7(6)
C3	C2	C1	114.5(2)	C15[2]	C17	C14	67.0(6)
N2	C8	S1	125.1(2)	C15[2]	C17	C18	127.5(8)
N2	C8	N3	119.7(2)	C15[2]	C17	C17[2]	125.6(9)
N3	C8	S1	115.2(2)	C16	C15	C14[2]	130.8(7)
N1	C6	C7	115.8(2)	C17[2]	C15	C16	66.1(6)
N1	C4	C5	113.5(2)	C17[2]	C15	C14[2]	64.7(6)
N2	C9	C10	109.9(2)				

[1]1-X,1-Y,1-Z; [2]-X,2-Y,1-Z

Crystallographic information for 7

Table S10: Crystal data and structure refinement for **7**

Identification code	xstr1300
Empirical formula	$C_{14.5}H_{24}N_3OSZn$
Formula weight	353.80
Temperature/K	149.94(10)
Crystal system	monoclinic
Space group	$P2_1/c$
a/Å	11.86410(10)
b/Å	8.38880(10)
c/Å	18.4350(2)
α/°	90
β/°	95.9940(10)
γ/°	90
Volume/Å^3	1824.72(3)
Z	4
ρ_{calc}g/cm^3	1.288
μ/mm^{-1}	2.938
F(000)	744.0
Crystal size/mm^3	0.2 × 0.1 × 0.1
Radiation	Cu Kα (λ = 1.54184)
2Θ range for data collection/°	9.648 to 145.316
Index ranges	-14 ≤ h ≤ 14, -10 ≤ k ≤ 10, -22 ≤ l ≤ 22
Reflections collected	28487
Independent reflections	3604 [R_{int} = 0.0275, R_{sigma} = 0.0117]
Data/restraints/parameters	3604/0/231
Goodness-of-fit on F^2	1.047
Final R indexes [I>=2σ (I)]	R_1 = 0.0321, wR_2 = 0.0883
Final R indexes [all data]	R_1 = 0.0330, wR_2 = 0.0892
Largest diff. peak/hole / e Å^{-3}	0.48/-0.41

Table S11: Bond lengths for **7**

**Atom**	**Atom**	**Length/Å**	**Atom**	**Atom**	**Length/Å**
Zn1	Zn1^1	2.9086(4)	C1	C2	1.476(7)
Zn1	S1	2.2508(5)	C9	C11	1.501(3)
Zn1	O1	1.9760(13)	C9	C10	1.486(4)
Zn1	O1^1	1.9794(12)	C3A	C2A	1.515(7)
Zn1	N1	2.0791(17)	C13	C16	1.287(8)
S1	C6	1.7843(18)	C13	C12	1.422(11)
O1	Zn1^1	1.9795(12)	C13	C14	1.553(11)
O1	C1	1.420(2)	C16	C16^2	1.731(11)
N3	C6	1.273(2)	C16	C12	1.123(9)
N3	C9	1.461(2)	C16	C15^2	1.408(7)
N2	C6	1.408(2)	C16	C15	1.533(7)
N2	C8	1.466(3)	C16	C14	1.366(9)
N2	C7	1.465(3)	C12	C15^2	1.251(9)
N1	C5	1.455(3)	C15	C16^2	1.408(7)

N1	C4	1.475(3)	C15	C12[2]	1.251(9)
N1	C3A	1.523(5)	C15	C14	1.242(9)
N1	C3	1.549(6)	C2	C3	1.496(11)
C1	C2A	1.512(5)			

[1]1-X,1-Y,1-Z; [2]-X,-Y,1-Z

Table S12: Bond angles for **7**

Atom	Atom	Atom	Angle/°	Atom	Atom	Atom	Angle/°
S1	Zn1	Zn1[1]	130.262(17)	C2A	C3A	N1	109.1(4)
O1[1]	Zn1	Zn1[1]	42.62(4)	C16	C13	C12	48.7(4)
O1	Zn1	Zn1[1]	42.71(3)	C16	C13	C14	56.6(5)
O1[1]	Zn1	S1	111.36(4)	C12	C13	C14	105.0(5)
O1	Zn1	S1	125.91(4)	C13	C16	C16[2]	171.8(7)
O1	Zn1	O1[1]	85.33(5)	C13	C16	C15[2]	129.9(6)
O1[1]	Zn1	N1	106.27(7)	C13	C16	C15	121.9(5)
O1	Zn1	N1	98.85(7)	C13	C16	C14	71.6(6)
N1	Zn1	Zn1[1]	107.17(5)	C12	C16	C13	71.9(6)
N1	Zn1	S1	122.21(6)	C12	C16	C16[2]	115.1(8)
C6	S1	Zn1	102.43(6)	C12	C16	C15[2]	58.0(6)
Zn1	O1	Zn1[1]	94.67(5)	C12	C16	C15	165.1(7)
C1	O1	Zn1[1]	124.70(12)	C12	C16	C14	143.0(8)
C1	O1	Zn1	122.51(13)	C15[2]	C16	C16[2]	57.4(4)
C6	N3	C9	120.60(16)	C15	C16	C16[2]	50.7(3)
C6	N2	C8	116.51(15)	C15[2]	C16	C15	108.0(4)
C6	N2	C7	114.36(15)	C14	C16	C16[2]	100.9(6)
C7	N2	C8	110.89(16)	C14	C16	C15	50.4(4)
C5	N1	Zn1	111.97(16)	C14	C16	C15[2]	158.0(6)
C5	N1	C4	106.9(2)	C16	C12	C13	59.4(6)
C5	N1	C3A	121.2(3)	C16	C12	C15[2]	72.5(5)
C5	N1	C3	93.8(3)	C15[2]	C12	C13	131.9(6)
C4	N1	Zn1	109.39(15)	C1	C2A	C3A	112.5(4)
C4	N1	C3A	96.8(3)	C16[2]	C15	C16	72.0(4)
C4	N1	C3	127.8(3)	C12[2]	C15	C16	121.3(5)
C3A	N1	Zn1	109.12(18)	C12[2]	C15	C16[2]	49.5(4)
C3	N1	Zn1	105.9(2)	C14	C15	C16	57.8(5)
N3	C6	S1	125.12(14)	C14	C15	C16[2]	129.6(5)
N3	C6	N2	119.10(16)	C14	C15	C12[2]	179.0(6)
N2	C6	S1	115.73(13)	C16	C14	C13	51.9(4)
O1	C1	C2A	112.9(2)	C15	C14	C13	123.7(6)
O1	C1	C2	112.8(3)	C15	C14	C16	71.8(4)
N3	C9	C11	109.49(18)	C1	C2	C3	118.6(6)
N3	C9	C10	109.2(2)	C2	C3	N1	113.5(4)
C10	C9	C11	111.9(3)				

[1]1-X,1-Y,1-Z; [2]-X,-Y,1-Z

D. SEM images

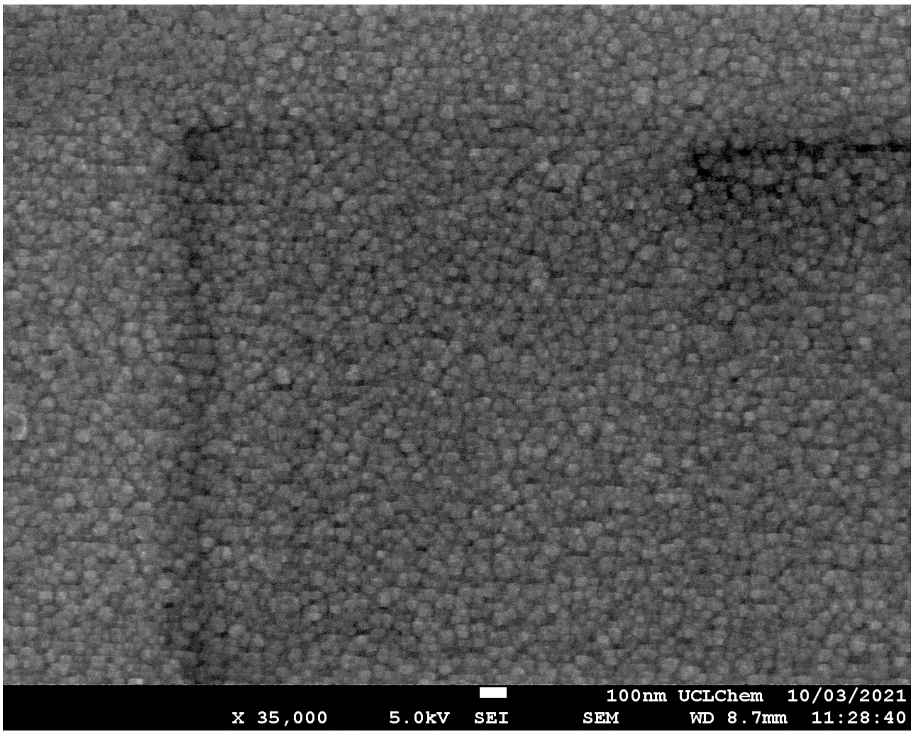

Figure S35: SEM image of film **B** showing particulates <100 nm in size.

E. XPS spectra

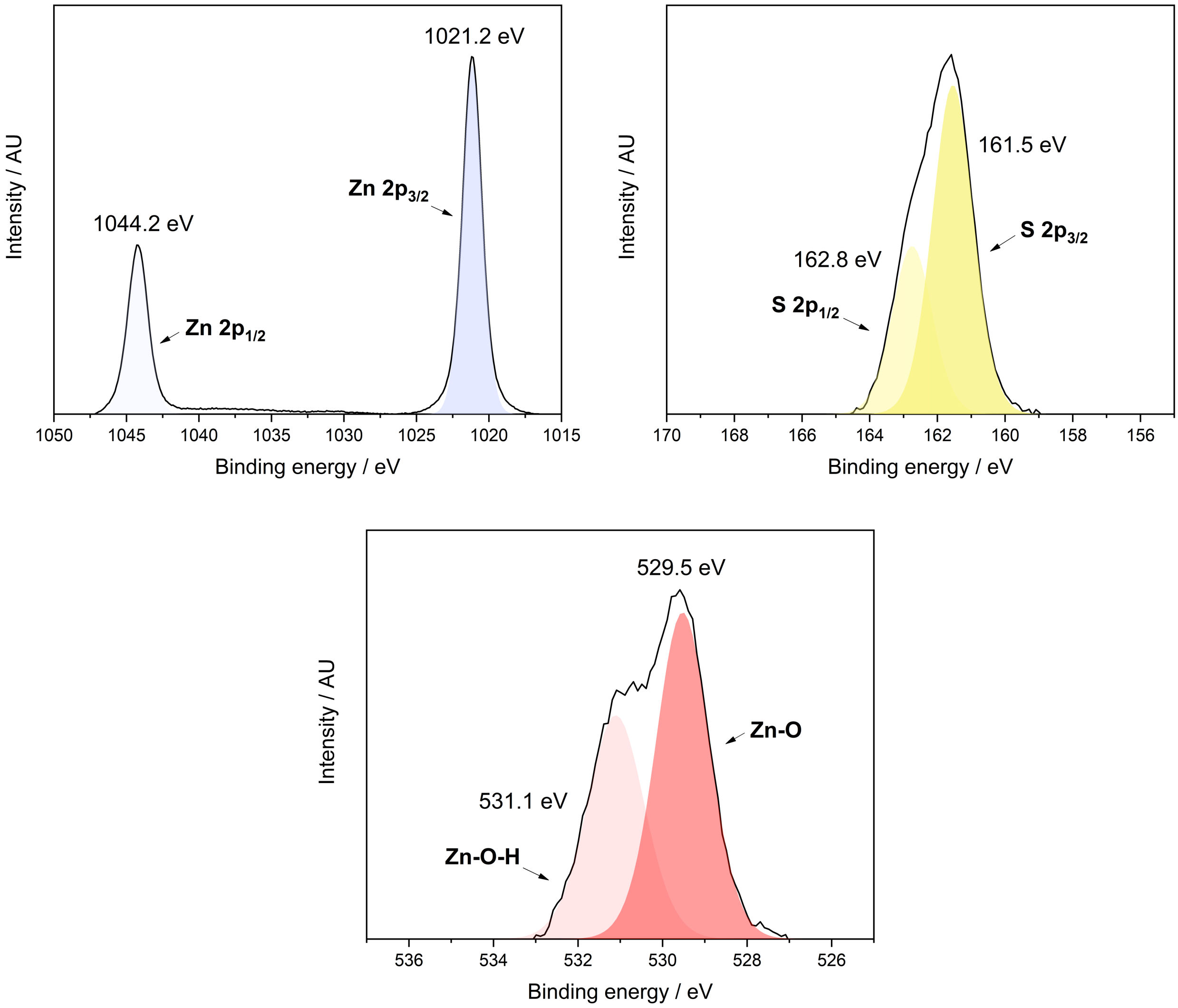

Figure S36: XPS spectra of film **C**. XPS of the Zn $2p_{1/2}$ and $2p_{3/2}$ states, top left; the S $2p_{1/2}$ and $2p_{3/2}$ states, top right; and the O 1s state, bottom, all at a 300 s etch.

F. Raman spectra

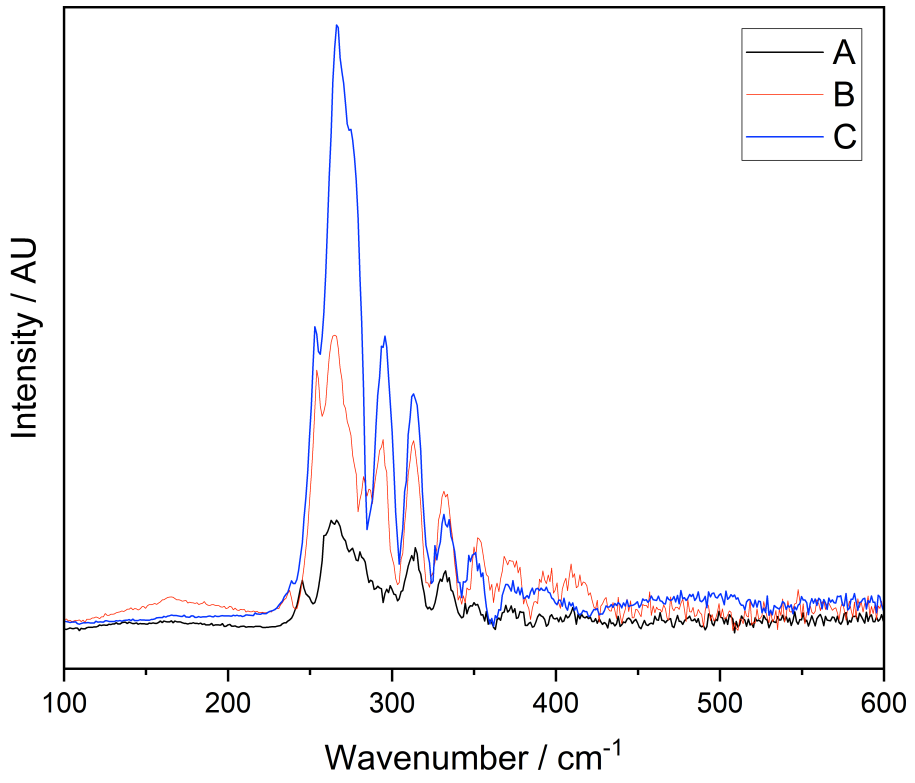

Figure S37: Raman spectra of Zn(O,S) films **A**, **B** and **C**.
